# Fluxome study of *Pseudomonas fluorescens* reveals major reorganisation of carbon flux through central metabolic pathways in response to inactivation of the anti-sigma factor MucA

**DOI:** 10.1186/s12918-015-0148-0

**Published:** 2015-02-18

**Authors:** Stina K Lien, Sebastian Niedenführ, Håvard Sletta, Katharina Nöh, Per Bruheim

**Affiliations:** Department of Biotechnology, Norwegian University of Science and Technology, Sem Sælands vei 6/8, N-7491, Trondheim, Norway; Institute of Bio- and Geosciences IBG-1: Biotechnology, Forschungszentrum Jülich, D-52425 Jülich, Germany; Department of Bioprocess technology, SINTEF Materials and Chemistry, Sem Sælands vei 2a, N-7465, Trondheim, Norway

**Keywords:** *Pseudomonas fluorescens*, Anti-sigma factor MucA, Fluxome and fluxomics, Carbon labelling experiments, GC-MS/MS, LC-MS/MS, ^13^C-metabolic flux analysis

## Abstract

**Background:**

The bacterium *Pseudomonas fluorescens* switches to an alginate-producing phenotype when the pleiotropic anti-sigma factor MucA is inactivated. The inactivation is accompanied by an increased biomass yield on carbon sources when grown under nitrogen-limited chemostat conditions. A previous metabolome study showed significant changes in the intracellular metabolite concentrations, especially of the nucleotides, in *mucA* deletion mutants compared to the wild-type. In this study, the *P. fluorescens* SBW25 wild-type and an alginate non-producing *mucA-* Δ*algC* double-knockout mutant are investigated through model-based ^13^C-metabolic flux analysis (^13^C-MFA) to explore the physiological consequences of MucA inactivation at the metabolic flux level. Intracellular metabolite extracts from three carbon labelling experiments using fructose as the sole carbon source are analysed for ^13^C-label incorporation in primary metabolites by gas and liquid chromatography tandem mass spectrometry.

**Results:**

From mass isotopomer distribution datasets, absolute intracellular metabolic reaction rates for the wild type and the mutant are determined, revealing extensive reorganisation of carbon flux through central metabolic pathways in response to MucA inactivation. The carbon flux through the Entner-Doudoroff pathway was reduced in the *mucA-* Δ*algC* mutant, while flux through the pentose phosphate pathway was increased. Our findings also indicated flexibility of the anaplerotic reactions through down-regulation of the pyruvate shunt in the *mucA*- Δ*algC* mutant and up-regulation of the glyoxylate shunt.

**Conclusions:**

Absolute metabolic fluxes and metabolite levels give detailed, integrated insight into the physiology of this industrially, medically and agriculturally important bacterial species and suggest that the most efficient way of using a *mucA-* mutant as a cell factory for alginate production would be to use non-growing conditions and nitrogen deprivation.

**Electronic supplementary material:**

The online version of this article (doi:10.1186/s12918-015-0148-0) contains supplementary material, which is available to authorized users.

## Background

*Pseudomonas* is a bacterial genus containing species of industrial, agricultural and medical relevance due to their metabolic diversity and ability to colonise a wide variety of ecological niches, including not only soil and water but also insects, plants, and animals [1,2]. The biosynthetic diversity of this genus includes production of the polysaccharide alginate, which has many commercial applications, especially in food and medicine [3], but alginate is a complicating factor to the human host during *Pseudomonas* infections [4].

Alginate is not produced by *Pseudomonas fluorescens* SBW25 wild type, but its production can be induced by inactivation of the anti-sigma factor MucA [5]. MucA inactivation is a frequent mechanism behind the conversion of *P. aeruginosa* to a mucoid phenotype infecting the lungs of cystic fibrosis patients [4]. In its active form, MucA prevents alginate biosynthesis by binding and sequestering AlgU, the sigma factor necessary for transcription of the *alg* operon. The *alg* operon codes for all of the structural genes needed for alginate synthesis except AlgC [6], the role of which is to convert mannose 6-phosphate (*M6P*) to mannose 1-phosphate (*M1P*). *M6P* is produced from fructose-6-phosphate (*F6P*), implying that alginate synthesis draws from the hexose-phosphate pool of central metabolism. In both *P. aeruginosa* and *P. fluorescens,* MucA inactivation has been shown to affect numerous genes other than those involved in alginate biosynthesis [5,7,8].

In our previous metabolome study [9], *P. fluorescens* SBW25 wild type and *mucA*-mutants were cultivated on fructose and glycerol as sole carbon sources. Intracellular concentrations of a wide range of metabolic intermediates, including phosphometabolites, amino acids, organic acids, and nucleotides, were measured. The study revealed significant carbon-source-dependent differences in primary metabolite and nucleotide levels for both the wild type and the *mucA* mutants. A dramatic decrease in energy charge (EC) was found in MucA mutant strains, which was accompanied by an increased growth yield (Y_X/S_). Interestingly, no counter response action of the energy-deprived mutants’ levels of central intermediates and metabolic genes was observed. This observation led to the hypothesis that mutants do not perceive the low EC as a critical energy limitation. To understand this puzzling robustness, the reorganisation of carbon flows within the biological network of *P. fluorescens* strains upon MucA inactivation needs to be elucidated. This is the aim of the present study.

In contrast to other “-omics” technologies, metabolic fluxes cannot be directly measured but must be inferred from carbon labelling experiments (CLEs) in combination with mathematical modelling [10]. In CLEs, ^13^C-enriched carbon substrates are fed to the cells and, driven by the cells’ metabolic and enzymatic activities, isotopically labelled carbon atoms are incorporated into the metabolite pools [11]. The incorporation of labelled material causes all central metabolic intermediates to exist as a set of different isotopomers, which are a collection of isomers that have different labelling patterns of ^12^C and ^13^C carbon atoms. Labelling data along with physiological data on observed fluxes (uptake and production fluxes) then enable intracellular fluxes to be quantified through ^13^C-metabolic flux analysis (^13^C-MFA) or, analogous to other “omics”-type analyses, fluxomics [12]. During the last few decades, ^13^C-MFA has become a mature tool to quantify the actual *in vivo* physiological state of microorganisms, as proven by numerous application studies from the fields of metabolic engineering and systems biology [13-15].

Published fluxome studies for *Pseudomonas* species are scarce. A flux ratio analysis comparing seven bacterial species (*P. fluorescens* wild type, *P. putida, Agrobacterium tumefaciens*, *Sinorhizobium meliloti*, *Rhodobacter sphaeroides*, *Zymomonas mobilis*, and *Paracoccus versutus*) with the two model organisms *E. coli* and *Bacillus subtilis* has been performed with glucose as the carbon source [16]. CLEs were carried out in shake flasks, and samples were taken during exponential growth to derive the relative net flux contribution of converging pathways in selected branch points of the metabolic network from the labelling patterns of protein-bound amino acids. The authors showed that compared to the model organisms, the seven investigated strains, including *P. fluorescens*, behaved significantly different, as they all mainly relied on the Entner-Doudoroff pathway (EDP) and used the pentose phosphate pathway (PPP) only for biosynthetic functions. In addition, the aerobic bacteria had no by-product formation and to a larger extent used the pyruvate shunt (malate (*MAL*) to pyruvate (*PYR*) to oxaloacetate (*OAA*)) for conversion of *MAL* to *OAA* instead of direct conversion by malate dehydrogenase [16]. In another study, intracellular fluxes for *P. aeruginosa* elucidated alginate production using glucose as the carbon source [17-19]. Significant involvement of the EDP in alginate biosynthesis was shown; glucose was shuttled via triose phosphate glyceraldehyde 3-phosphate (*GAP*) and dihydroxyacetone phosphate (*DHAP*) to alginate.

The objective of the current study is to quantify the effect of MucA inactivation on the primary metabolism of *P. fluorescens* through determination of the levels of absolute fluxes. For comparability with experiments promoting alginate production [5] on the one hand and the conditions of our previous metabolomics study [9] on the other hand, nitrogen limitation was imposed and fructose was used as the carbon source. To investigate the effect of a *mucA* mutant, we focus on the flux reorganisation of the primary metabolism. However, alginate production starts upon MucA inactivation [4], which affects the metabolic fluxes due to the removal of the alginate precursor metabolite *M6P*, concurrently with the effects of MucA inactivation itself. To separate these two effects, we use a *mucA-* Δ*algC* double-knockout strain in which the direct effect of MucA inactivation can be observed without indirect “distortion” by alginate production. It was previously shown that a single deletion of *algC* does not alter gene expression [5] or metabolite profiles [9] compared to the wild-type strain. The determined absolute flux distributions are compared with data from the previous metabolome study conducted with the same strains. The comparison of both flux maps and metabolite levels generates complementary views of the primary metabolism of *P. fluorescens*. In particular, flux maps reveal new insights into the mechanisms by which primary metabolism is controlled by MucA, and how MucA may influence alginate production through the re-routing of carbon flow. In summary, this study provides the first integrative perspective on the metabolism of *P. fluorescens* and lays the groundwork for the future metabolic engineering of this organism.

## Results

### Chemostat cultivations and carbon labelling experiments (CLE)

To quantify the changes in carbon flows in *P. fluorescens* upon MucA inactivation, the wild-type strain and an alginate non-producing *mucA-* Δ*algC* double-knockout mutant were grown with fructose as the sole carbon source under nitrogen limitation. For ^13^C-MFA, it is essential to ensure that cell physiology and metabolism are held in a pseudo-steady state throughout the experiment. Therefore, CLEs are conducted in chemostats with growth rates of 0.04 h^-1^ under defined environmental conditions. CLEs were performed in two rounds: results derived from one wild-type cultivation (a “pre-CLE”) were used for an experimental design study to determine a statistically maximally informative fructose isotopomer composition, as detailed in the Materials and Methods section. In turn, the calculated mixture was administered in the main CLEs to the two strains.

The specific rates for fructose uptake and biomass production in nitrogen-limited chemostats are shown in Table 1 (carbon source in excess). The wild-type and the *mucA- ΔalgC* strains show similar specific biomass formation rates. Interestingly, the specific fructose consumption rate is almost 40% lower in the *mucA- ΔalgC* strain, causing a higher biomass yield on carbon source (Y_X/S_) for the mutant (19% for the *mucA-ΔalgC* strain compared to 12% for the wild-type).

**Table 1:** **Measured and calculated cultivation data for** ***P. fluorescens*** **CLEs**

**Strain**	**OD**_**660**_	**Specific fructose uptake [mmolC/gDW h]**	**Specific alginate production [mmolC/ gDW h]**	**Specific biomass production [mmolC/gDW h]** ^**3)**^	**Specific CO**_**2**_ **excretion [mmol/gDW h]** ^**4)**^	**Y**_**xs**_ **[%]**
wild-type^1)^	8.0±0.1	11.0±0.6	0.0	1.4±0.13	9.6±0.61	12
wild-type^2)^	7.0±0.1	11.8±0.6	0.0	1.4±0.13	10.4±0.61	12
Estimated^5)^		11.76±0.54		1.34±0.13	10.41±0.55	
*mucA-*Δ*algC* ^2)^	8.9±0.1	7.34±0.4	0.0	1.4±0.13	6.0±0.42	19
Estimated^5)^		7.39±0.32		1.30±0.13	6.08±0.34	

^1)^Experimental design cultivation on wild type. ^2)^Main experiment on both strains. ^3)^Calculated from culture growth rate and biomass composition based on biomass equation. ^4)^Difference between fructose uptake and biomass production. No additional carbon-containing products (i.e., organic acids) were detected in the culture supernatant. ^5)^ Estimated rates from simulation. Standard deviations are estimated from the process data and used for the ^13^C modelling.

### Mass spectrometry measurements

Metabolite extracts from the CLEs were analysed by LC-MS/MS and GC-MS/MS to generate mass isotopomer datasets. Both chromatographic techniques were used to increase the information content and the coverage of labelling patterns for flux estimation. GC-MS was mainly used to analyse soluble free amino acids, while LC-MS was used for central carbon metabolites (organic acids and sugar phosphates). Based on the MS/MS datasets, intracellular fluxes were estimated for both strains.

### *Quantification of metabolic fluxes for the wild-type and mucA-* Δ*algC strains*

A model of *P. fluorescens* central carbon metabolism was formulated that incorporated reactions for fructose uptake (Carbon Uptake), glycolysis/Embden – Meyerhof – Parnas pathway (EMP), the pentose phosphate pathway (PPP), the Entner-Doudoroff pathway (EDP), the tricarboxylic acid cycle (TCA) and glyoxylate shunt, the anaplerotic reactions (ANA), as well as amino acid biosynthesis pathways (BM/BS), covering the measured metabolite pools (cf. Additional file 1 for specification of the metabolic network model including carbon atom transitions). Details of the ^13^C-MFA modelling workflow are given in the Materials and Methods section.

**Figure 1:**
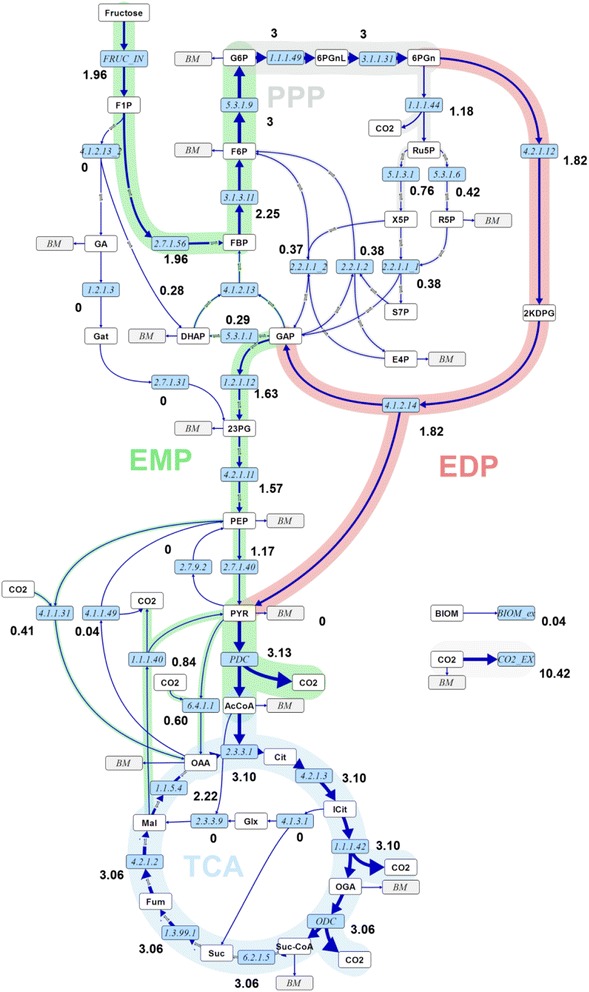
**Flux map for** ***P. fluorescens*** **SBW25 wild type.** Absolute net fluxes of central carbon metabolism in mmol/(gDW h) and BIOM_ex given as gDW/(gDW h) for *P. fluorescens* SBW25 wild type. Net fluxes are visualised by the thickness of the reaction arrows (logarithmic scale). Numerical values for the complete flux distribution, consisting of net and exchange fluxes along with their standard deviations, can be found in Additional file 2. For abbreviations of metabolite names, see Additional file 1.

**Figure 2:**
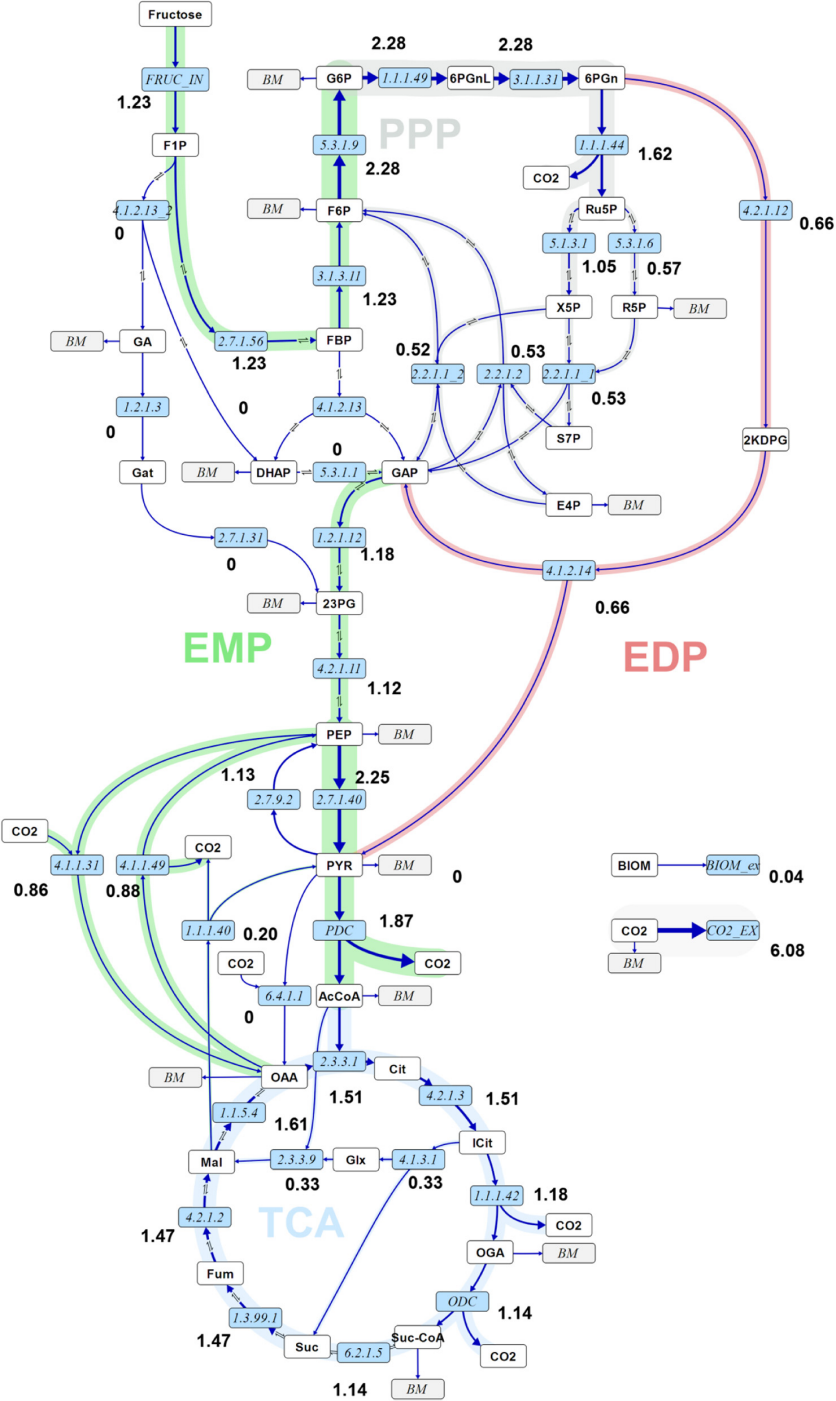
**Flux map for** ***P. fluorescens*** **SBW25** ***mucA-*** **Δ** ***algC*** **strain.** Absolute net fluxes of central carbon metabolism in mmol/(gDW h) and BIOM_ex given as gDW/(gDW h) for the *P. fluorescens mucA-* Δ*algC* strain. Net fluxes are visualised by the thickness of the reaction arrows (logarithmic scale). Numerical values for the complete flux distribution, consisting of net and exchange fluxes along with their standard deviations, can be found in Additional file 2. For abbreviations of metabolite names, see Additional file 1.

Calculated absolute intra- and extracellular fluxes for the wild-type and *mucA-* Δ*algC* strains based on the two main CLEs, are visualised in Figures 1 and 2, respectively, along with the values for net (n) fluxes (difference of forward flux and backward flux rates – see [20] for an in-depth explanation). The flux distributions with bidirectional resolution (i.e., net and exchange fluxes), including the drain from metabolic intermediates to biomass and confidence intervals of the flux estimates, are found in Additional file 2, along with a presentation of all net fluxes for the two strains relative to each other. Net fluxes proceed in the same direction for the two strains (the opposite directions of aldolase (EC 4.1.2.13) and triose phosphate isomerase (EC 5.3.1.1) in glycolysis are not statistically significant). The comparison between the real and the in silico rates (estimated) in Table 1 demonstrate that measurements and model predictions match fairly well.

**Figure 3:**
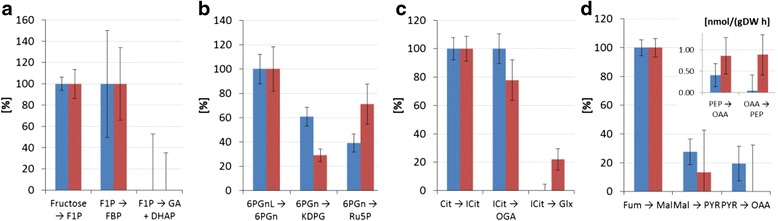
**Detailed inspection of four important branch points in central carbon metabolism.** Relative net fluxes for *P. fluorescens* SBW25 (blue) and the *mucA-* Δ*algC* strain (red) at **(a)** Fructose uptake branch point: effluxes as percentage of influx to *F1P*; **(b)** EDP versus PPP branch point: effluxes as percentage of influx to *6PGN*; **(c)** TCA versus *GLX* shunt branch point: effluxes as percentage of influx to *ICIT*; **(d**) ANA branch points: net fluxes from *MAL* to *PYR* and from *PYR* to *OAA* as percentage of influx to *MAL* (main chart) and absolute values for *PEP* to *OAA* fluxes (inset). List of abbreviations given in Additional file 1.

The first branch point in the fructose uptake pathway is fructose 1-phosphate (*F1P*), which can be converted either to fructose 1,6-bisphosphate (*FBP*) or to *DHAP* and glyceraldehyde (*GA*). *GA* in turn enters central carbon metabolism either as 3-phosphoglycerate or as 2-phosphoglycerate (symbolised by the pooled entry *23PG*) via conversion to glycerate (*GAT*). The flux maps reveal that fructose uptake via *F1P* to *FBP* is the main route for both strains (see EC 2.7.1.56 versus EC 4.1.2.13 in Figures 1 and 2 and the relative comparisons of fructose uptake fluxes in Figure 3A). Furthermore, the main efflux from *FBP* for both strains is directed to glucose 6-phosphate (*G6P*) via *F6P*. The EDP is thus preferred over the glycolytic route for both strains when growing on fructose, in spite of the enzymatic capacity for the direct conversion of *FBP* to *DHAP* and *GAP* (conversion of fructose to these trioses does not rely on the missing phosphofructokinase activity, which is required for conversion of glucose via the glycolytic route).

Although the wild-type and the *mucA-* Δ*algC* strains both utilise the same main route for initial fructose catabolism, there are differences between the two strains at important branch points. One of the most striking distinctions occurs at the 6-phosphogluconate (*6PGN*) node, where the EDP and the PPP split. In the wild-type strain, the major carbon flux from the 6PGN node is directed to the EDP (61 ± 8%, EC 4.2.1.12), whereas the minor proportion goes to the PPP (39 ± 7%, EC 1.1.1.44) (Figures 1 and 3B). However, the situation is reversed for the *mucA-* Δ*algC* strain. As shown in Figures 2 and 3B, the major carbon flux goes to the PPP (71 ± 17%) and the minor efflux for this strain is directed to the EDP (29 ± 5%). Notably, even with an almost 40% decrease in the fructose uptake rate, the *mucA-* Δ*algC* strain still has a higher absolute flux through the PPP (1.6 ± 0.3 mmol/(gDW h) versus 1.18 ± 0.20 mmol/(gDW h) for the wild-type strain). It is thus clear that inactivating the anti-sigma factor MucA leads to a reduced flux through the EDP and an increased flux through the PPP.

The second important branch point that is found to be distinct for the two strains is isocitrate (*ICIT*) in the TCA. The wild-type strain does not utilise the glyoxylate (*GLX*) shunt to any significant extent (flux value of 0% from *ICIT* to *GLX* with an asymmetric confidence interval [0, 4]% corresponding to an absolute confidence interval of [0.00,0.10] mmol/(gDW h)), while the *mucA-* Δ*algC* strain shuttles 22 ± 7% (0.33 ± 0.16 mmol//(gDW h)) of the *ICIT* influx to *GLX* (cf. Figures 1, 2 and 3C). The fructose uptake rate and EDP flux are lower for the *mucA-* Δ*algC* strain than for the wild-type, leading to a significantly lower influx to *ICIT* compared to the wild type (1.51 ± 0.09 mmol/(gDW h) vs. 3.10 ± 0.17 mmol/(gDW h)). Thus, the utilisation of the *GLX* shunt in response to MucA inactivation in turn leads to an even further reduction in flux through the remainder of the TCA.

Finally, comparison of the flux maps reveals differences between the two strains, although the current dataset does not allow for the precise estimation of ANA flux values (see broad confidence intervals in Additional file 2). Nevertheless, the results indicate that the *mucA-* Δ*algC* strain might cycle phosphoenolpyruvate (*PEP*) to *OAA* and back, with the two net fluxes almost cancelling each other out (to replace the drain of TCA intermediates to biomass, a small net flux to TCA remains). In contrast, for the wild-type strain, the flux from *PEP* to *OAA* might be significantly higher than the reverse flux (cf. Figures 1, 2 and inset of Figure 3D portraying the magnitude of the net fluxes). The results also show that although the *PYR* shunt (*MAL* → *PYR* → *OAA*) is utilised to a significant extent by the wild-type strain, shuttling 28 ± 9% (0.84 ± 0.27 mmol/(gDW h)) of the *MAL* influx to *OAA* via *PYR*, this shunt is significantly less active in the *mucA-* Δ*algC* strain (a flux of 13% or 0.20 mmol/(gDW h) within the confidence range of [0, 0.45] mmol/(gDW h) of the *MAL* influx goes to *PYR*) (Figures 1, 2 and main chart of Figure 3D). Thus, down-regulation of the pyruvate shunt must be a consequence of MucA inactivation.

### NADH and NADPH production is significantly higher in wild type

**Figure 4:**
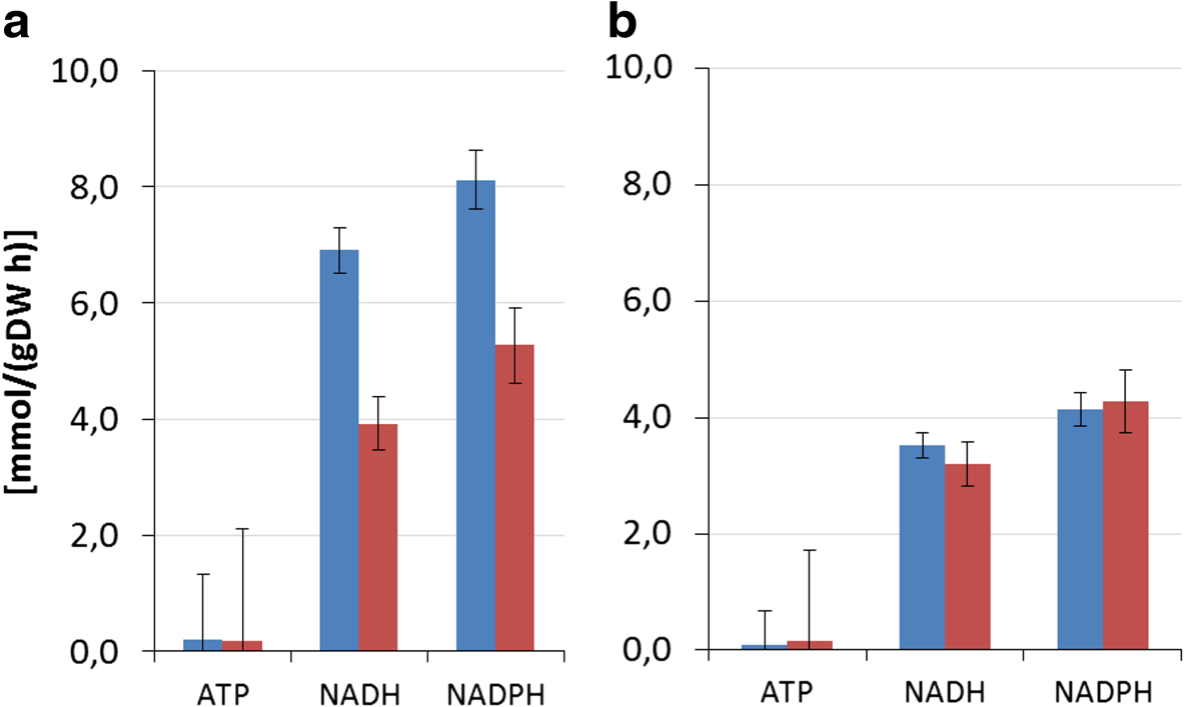
**ATP, NADH and NADPH production.** Reactions involving *ATP* and producing reducing power by central carbon metabolism of *P. fluorescens* SBW25 (blue) and the *mucA-* Δ*algC* strain (red); **(a)** based on absolute fructose rate, and **(b)** normalised to the respective strain’s uptake rate. See Additional file 2 for the contributing fluxes.

Figure 4 displays the net production of *ATP*, *NADH* and *NADPH* in the primary metabolic network based on the estimated absolute fluxes (A) and normalised to fructose uptake for relative (B) comparison between the mutant and the wild type (detailed rates are given in Additional file 2). For both strains, the *ATP*-consuming reactions almost cancel out *ATP* production by substrate-level phosphorylation. This leads to a similarly low net *ATP* production for both strains. Thus, both strains use oxidative phosphorylation for the majority of *ATP* synthesis, as expected.

In contrast, the net production of *NADH* and *NADPH* differs for the two strains, both being higher for the wild type (Figure 4A). However, the relative proportion of NADH to NADPH production is approximately the same, even though there are large differences in major intracellular carbon flow between the two strains (Figure 4B, with data normalised to the fructose uptake rate). In the wild-type strain, the production of *NADH* is significantly higher because of higher uptake of fructose, more of which is shuttled through the EDP and to the TCA cycle than in the *mucA-* Δ*algC* strain. Likewise, *NADPH* production is higher for the wildtype, again predominantly because of the wildtype’s higher TCA flux (isocitrate dehydrogenase is *NADPH*-dependent, EC. 1.1.1.42). On a relative scale, compared to the influx of the network (Table 1), *NADPH* production is similar in both strains. Because biomass production compared to substrate uptake is higher for the *mucA-* Δ*algC* strain, the wild-type strain generates a surplus of *NADPH* that is not needed for anabolic purposes.

## Discussion

*Pseudomonas fluorescens* is an attractive organism in industrial, medical and agricultural biotechnology. The first step towards optimising alginate production with this microorganism is to accurately characterise the influencing factors and underlying metabolic regulation mechanisms that reorganise the carbon flow of alginate-producing mutant strains in comparison to the wildtype. This study investigated how the effect of MucA modulates the carbon flow under conditions favouring alginate production. To this end, information-optimised CLEs were designed and, in turn, conducted in well-controlled chemostats under steady-state conditions. Mass isotopomer data were collected from LC- as well as GC-coupled MS devices, generating comprehensive labelling datasets with high coverage. Labelling data, in combination with measured uptake and biomass formation rates, enable the precise determination of absolute intracellular flux rates by ^13^C-MFA. Finally, the fluxome of *P. fluorescens* is related to previously determined transcriptome and metabolome data.

Our fluxome study revealed significant reorganisation of major intracellular metabolic fluxes due to inactivation of the pleiotropic anti-sigma factor MucA. The most significant findings from the *mucA-* Δ*algC* double-knockout mutant are as follows: 1. a reduction in EDP flux, 2. an increase in PPP flux, and 3. an activation of the *GLX* shunt. The data for the wildtype pre-experiment and the two cultivations of the main experiment (Table 1) coincide well with previously published data for *P. fluorescens* cultivations under similar conditions [5,9], thus confirming that the results from these down-scaled CLE cultivations (120 mL cultivations) can be correlated with transcriptome and metabolome results generated at standard lab-scale operating conditions (750 mL). The experimental set-up with nitrogen-limited chemostats provides the sole carbon source, fructose, in excess. The cells can therefore adjust their carbon source consumption to their metabolic requirements.

### Reduced fructose uptake rate and EP pathway flux in the mucA- ΔalgC mutant with lowest energy charge

Interestingly, the wild-type strain has a 60% higher fructose uptake rate than the *mucA-* Δ*algC* strain (Figures 1, 2 and Table 1). Because the growth rate of both strains is the same, the biomass yield on substrate (Y_XS_) is 60% higher in the *mucA-* Δ*algC* strain. Thus, the wildtype either uses more of the fructose for maintenance processes, has a less energy-efficient metabolism (i.e., lower P/O ratio), and/or disposes of surplus energy through futile cycling, which was frequently described for other organisms such as *Escherichia coli*, *Bacillus subtilis*, and *Corynebacterium glutamicum* [16,21-24].

The reduction in fructose uptake in the *mucA-* Δ*algC* strain cannot be a direct effect of MucA inactivation alone, as an alginate-producing *mucA*- single-deletion mutant had a two-fold increased fructose uptake rate in the metabolome study (18.3 mmol C/(gDW h) in the *mucA-* mutant vs. 9.4 mmol C/(gDW h) in the wild type) [9]. MucA inactivation thus seems to exert its effect on the EDP, the PPP and the TCA rather than on the uptake system and the initial reactions of *F6P* and *G6P* alone.

The previous metabolome study [9] showed metabolite pool changes by MucA inactivation in the presence and absence of alginate production. The most striking result was a significantly decreased EC ((*ATP* + 0.5*ADP*)/(*ATP* + *ADP* + *AMP*)) in both the alginate-non-producing *mucA-* Δ*algC* mutant used in this fluxome study and an alginate-producing *mucA-* single-knockout mutant [9]. The reduction in EC was caused both by a decrease in *ATP* concentration and an increase in *ADP* and *AMP* concentrations, the latter almost 10 times higher. The higher EC of the wild type correlates with its larger *NADH* production (Figure 4). However, the *mucA-* Δ*algC* strain does not exploit the full capacity to increase its EC in the way the wild type does by, for example, increasing fructose uptake. The likely reason for this is that *mucA-* mutants do not perceive their relatively low EC as a stress situation, and therefore, no cellular adaptation response is stimulated.

In theory, *P. fluorescens* could utilise glycolysis alone for fructose assimilation, converting *FBP* to *PYR* and generating two net *ATP*. By converting *FBP* to *PYR* via the EDP, no net *ATP* is generated, as no *ATP* is formed via fructose-1,6-bisphosphatase (EC 3.1.3.11). From this perspective, both *P. fluorescens* strains preferably use the less energy-efficient EDP route. Flamholz and co-workers analysed glycolysis and the EDP in terms of thermodynamic and kinetic constraints and concluded that glycolysis incurs a higher protein cost (i.e., more enzymatic proteins are needed to sustain the same flux) than the EDP [25]. The lower protein cost of the EDP might especially favour this pathway under the studied conditions because the chemostats were nitrogen limited, raising the pressure on protein synthesis. Thus, nitrogen limitation seems advantageous for alginate production on fructose.

Concerning the preferred route for fructose uptake, it is also interesting to note that FBPase in *P. fluorescens* is negatively regulated by *AMP,* as it is in other bacteria, e.g., *E. coli* [26]. Because alginate synthesis starts with the conversion of *F6P* to *M6P*, the induction of alginate synthesis in the *mucA-* mutant must alleviate the possible rate-limitation of the FBPase. The eight-fold increase in the *mucA-* mutants’ *AMP* concentration could have led to the direct conversion of *FBP* to *DHAP* and *GAP* by FBP aldolase (EC 4.1.2.13) in the *mucA-* Δ*algC* strain, but that was not observed in the current fluxome study.

### Disposal of surplus energy in the wild-type strain

According to our fluxome study, a large proportion of the excess fructose uptake of the wild type is shuttled via EDP to TCA and complete oxidation to CO_2_, with energy conservation taking place through oxidative phosphorylation. This occurs with no obvious use of this extra energy compared to the *mucA-* Δ*algC* strain. Based on current knowledge of co-factor dependencies [5], the *NADPH* as well as *NADH* production are higher in the wild type (Figure 4). Strikingly, the *NADPH* production is higher than needed for anabolic processes in the wild type because the biomass production rate is the same for both strains. The cyclic operation of anaplerotic reactions in the wild type points to futile cycling dissipating energy by hydrolysing energy equivalents, as is also hypothesised for *Corynebacterium* species [27-29]. The wild type uses the *PYR* shunt, which generates one *NADPH* and consumes one *ATP*, which is a less energy-conserving solution than the direct oxidation of *MAL* to *OAA* with the *NADH*-producing *MAL* dehydrogenase reaction. It has been previously demonstrated that in batch cultivations growing on glucose, *P. fluorescens* primarily relies on the *PYR* shunt for conversion of *MAL* to *OAA* in contrast to other Gram-negative (*E. coli*, *P. putida*, *A. tumefaciens*, *R. sphaeroides* and *Z. mobilis*) and Gram-positive (*B. subtilis* and *P. versutus*) bacteria [16]. Thus, the pyruvate shunt might be active in situations where *NADH* production is not required.

Yet there remain open questions: how does the cell regenerate or allocate *NADP* if needed? Activation of a transhydrogenase (EC 1.6.1.1) may provide an explanation to this question [30]. Due to the lower fructose uptake rate of the *mucA-* Δ*algC* strain, it is unlikely that this mutant is energy limited. It is puzzling that the mutant activates the *GLX* shunt, which is mostly associated with growth on two-carbon substrates, and generates one net *OAA* from acetyl-CoA without any energy conservation. Additionally, and similarly to the wild type, the ANA cycling of the *mucA-* Δ*algC* strain through *PEP* carboxylase (EC 4.1.1.31) and *PEP* carboxykinase (EC 4.1.1.49) could be regarded as a futile cycle because one net *ATP* is consumed. However, the extent of futile cycling could not be quantified because, as mentioned, ANA reactions were not resolved in the forward and backward directions with statistical confidence. To this end, further CLEs with specially designed ^13^C-label mixtures are needed [28,31].

### Comparison with other -omics data

The comparative investigation of changes in the transcriptome [5] and the fluxome of the investigated strains reveals that the strong reorganisation of central metabolic carbon flows is not tightly coupled to expression levels of the corresponding metabolic genes, which remained remarkably constant.

**Figure 5:**
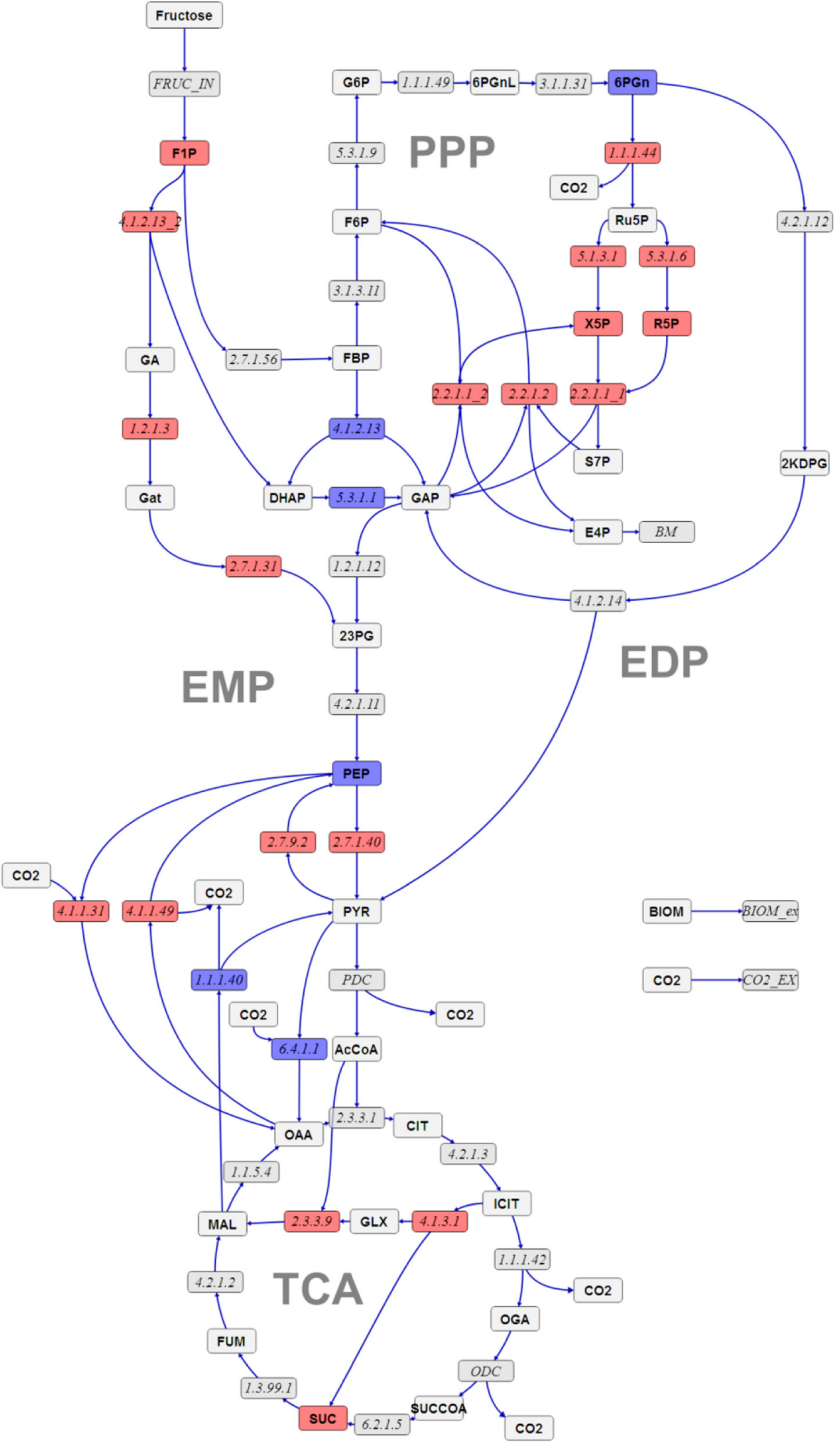
**Changes in flux values and metabolite concentrations.** Red fluxes/metabolite pools indicate a more than 150% increase in the mutant *mucA-* Δ*algC* strain compared to the wild type. Blue fluxes edges/metabolite pools indicate a decrease to less than 50% in the mutant *mucA-* Δ*algC* strain compared to the wild type.

Additionally, and not surprisingly, direct comparison of changes in the metabolome and the fluxome do not show any clear correlation: while the net flux through TCA was reduced by 50% in the *mucA*- Δ*algC* strain and the glyoxylate shunt was activated, the pool sizes of the TCA metabolites were not altered, except for the pool size of succinate, which increased by a factor of 2.4 (cf. Figure 5). Arguably, there was a weak positive correlation between the minor increase in both PPP metabolite pools and PPP flux in the *mucA*- Δ*algC* strain compared to the wild type. The concentration of the *F1P* pool increased eight-fold in the *mucA-* Δ*algC* strain, with a 40% reduced fructose uptake rate compared to wild type, but the same *F1P* pool increase was also observed in an alginate-producing *mucA-* mutant with a doubled fructose uptake rate compared to wild type. This change in the *F1P* pool could have global metabolic effects through modulating the actions of the catabolite repressor/activator protein Cra (also known as FruR). Cra is a global sensor and regulator of primary metabolic fluxes in Gram-negative bacteria, with a dual role as both a transcriptional repressor and an activator [32,33]. For example, both PPP and EDP genes were up-regulated in an *E. coli cra-* mutant, indicating that Cra represses these pathways [34]. *F1P* is the preferred effector of Cra in *P. putida*, and FBP is probably a minor effector as well [35]. No strong effect on the gene expression levels of metabolic genes, either through *F1P* mediated by Cra, or directly through MucA inactivation, was observed in *P. fluorescens* [5], but this work indicates a link, at the metabolic level, between *F1P* pool changes and metabolic flux reorganisation. Hence, metabolome and fluxome data provide a “true” orthogonal view of the metabolism of *P. fluorescens*, in the sense that understanding how cells modulate metabolic fluxes in response to interventions requires integrative multi-omics studies.

## Conclusion

In this work, a *mucA-* Δ*algC* double-knockout *P. fluorescens* mutant was compared to the wild-type strain at the metabolic flux level to illuminate the global metabolic response to MucA inactivation on central carbon metabolism without a biasing carbon re-routing by alginate production. Corroborating transcriptome and metabolome results from former studies, a strong influence of the *mucA-* Δ*algC* double-knockout mutant was shown, involving fine-tuned and significant reorganisation of carbon utilisation. Salient alterations lead to a decrease of the overall fructose uptake and an activation of the PPP and the GLX shunt that is accompanied by a deactivation of the EDP. Although both strains produce a similar amount of energy (NADH and ATP) per mole of substrate, the mutant seems to exhibit more energy-efficient behaviour than the wildtype.

Clearly, to mechanistically elucidate the metabolic control mechanisms of alginate production on fluxes, *mucA*- mutants need to be further investigated. These experiments bear the potential to elucidate the complex control mechanisms of MucA on alginate production and central carbon metabolism. These control points may become important targets for drug development to prevent the formation of the mucoid phenotype, which leads to pulmonary infection in cystic fibrosis patients, or to increase industrial alginate production. This requires extending the multi-omics characterisation to further mutants, including catabolite repressor/activator *cra* mutants, and experimental conditions, i.e., growth rates and carbon sources, that are clinically and industrially relevant. From the modelling point of view, the application of non-stationary ^13^C-MFA in combination with advanced experimental design strategies would be advantageous to advance the integrative analysis of metabolite levels and fluxes and increase the statistical significance of the results, particularly with regard to anaplerosis.

## Methods

### Strains

The strains used in this study were *P. fluorescens* SBW25 and *P. fluorescens* SBW25 *mucA-*Δ*algC*. Construction of the mutant strain was previously presented in detail [5]. Flux distribution results for these strains can be compared to metabolome results [9] for the same strains grown under identical conditions. A confounding factor when using the *mucA-*Δ*algC* strain to look for effects of MucA inactivation other than alginate production is that it produces all biosynthetic enzymes required for alginate production other than AlgC.

### Cultivations and sample preparation

Chemostat cultivations were performed as described earlier [5], except that the working volume was scaled down from 750 mL to 120 mL in a custom-made 0.5-L reactor vessel and the carbon source concentration was scaled down from 40 g/L to be less in excess, using the equivalent of 25 g/L and 20 g/L of naturally labelled fructose for the wild type and the *mucA-* Δ*algC* strain, respectively. These carbon source concentrations lead to a fructose concentration of 2.5 g/L or above in the fermenters at steady state. In brief, the chemostat cultivations were nitrogen limited using fructose as a carbon source with a dilution rate of 0.04 h^-1^. Temperature was maintained at 25°C, pH was maintained at 7.8 by automated sodium hydroxide addition, air flow was 1.5 VVM, and oxygen saturation was maintained above 20% by adjustment of the stirring speed. Cultivations were operated as batches for 24 h before initiating continuous operation with a feed containing naturally labelled fructose. After 1.5-2.0 volume exchanges, the feed stock was changed. Afterwards, the feedstock contained ^13^C-labelled fructose (two different lots of 1-^13^C-fructose with chemical purities of 99.1 and 98.0% (w/w) and isotope enrichments of 99% (mole/mole); and one lot of U-^13^C-fructose with chemical purity of 100% (w/w) and isotopic enrichment of 99.5% (mole/mole), Cambridge Isotope Laboratories, Inc, Andover). Sampling was performed after an additional 3 volume exchanges. Process data of double knock out mutant is given in Additional file 3.

Three cultivations were performed; the only difference between the first and the two last was the fructose isotopomer composition used in the ^13^C-labelled feed. In the first cultivation, the wild-type strain was grown using a 60.04% 1-^13^C-, 20.31% U-^13^C- and 19.65% ^12^C-fructose isotopomer composition (values based on experimental weight of dry substances adjusted for chemical impurities). In the two main cultivations, the wild type and the *mucA-*Δ*algC* strain were grown with the designed fructose isotopomer mixture of 60.08% 1-^13^C-, 39.92% U-^13^C-fructose.

Immediately prior to sampling for metabolite extracts, an aliquot of 5 mL was taken from the culture for OD_660_ and residual fructose measurement. The CO_2_ concentration in the off gas was measured with a Rosemount Binos 100 CO_2_ analyser. These data were used to calculate the carbon balance as described previously [5]. For preparation of metabolite extracts, the cultivations were sampled two (wild-type experimental design cultivation samples for LC-MS/MS analysis) or three (for all other conditions) times for each of the two MS-methods (biological replicates) to be employed, using fast vacuum filtration and quenching of metabolism in 37.5% cold methanol (-20°C). Details of the sampling method are as described in our previous metabolome study [9], except that only one filter with pore size 0.8 μm (Millipore cat#AAWP04700) was used instead of a stack of three filters and the culture volume filtered was 6 mL instead of 2 mL, leading to metabolites from 3 mL of culture being present in the final freeze-dried metabolite extracts.

### Sample and evaluation of mass isotopomer datasets

Metabolite extracts were analysed by an LC-MS/MS method and a GC-MS/MS method to generate mass isotopomer distribution datasets for the *P. fluorescens* SBW25 wild-type and the *mucA-*Δ*algC* strains. Prior to LC-MS/MS analysis, samples were reconstituted in 500 μL 60% (v/v) methanol. Analysis was performed on an Agilent 1200 series LC connected via an electrospray ion source to an Agilent 6410 triple-quadrupole MS using an adaptation of the reverse phase tributylamine ion exchange method introduced by Luo et al. [36]. The same LC-MS/MS method was used in the metabolome study of *P. fluorescens* [9]. A total of eight central carbon metabolism metabolites were analysed for mass isotopomer distributions by LC-MS/MS and used in the study. For each compound, the number of precursor-to-fragment transitions (multiple reaction monitoring (MRM)-transitions) was expanded to account for all mass isotopomers of the compound, i.e., (*n*-*m* + 1)(*m* + 1) MRM-transitions for each compound, where *n* is the number of carbons in the precursor ion and *m* is the number of carbons in the product ion, respectively [37]. A list of all MRM-transitions and accompanying MS-settings for the LC-MS/MS method can be found in Additional file 4.

Samples for GC-MS/MS analysis were derivatised prior to analysis as described previously [9] using an adaptation of the methyl chloroformate derivatisation protocol [38,39]. Analysis was performed on an Agilent 7890A GC – 7000 triple-quadrupole MS interfaced with a chemical ionisation (CI) ion source based on a previously described MS/MS method [40]. The GC method was as described previously [9], with the exception that the injected volume was increased from 1 μL to 3 μL. The MS method used positive CI with methane as the reagent gas and nitrogen as the collision gas. MRM-transitions to account for all mass isotopomers of the 14 detected compounds (central carbon metabolism metabolites and amino acids) were included in the fluxomics models. A list of all MRM-transitions and accompanying GC-MS/MS-settings can be found in Additional file 4.

The resulting mass isotopomer fractions are listed in Additional file 5. Average relative standard deviations (STD) for the mass isotopomers are calculated from the biological replicates for the estimation of intracellular fluxes. The obtained LC-MS/MS STD values were 13% for the wild-type experimental design cultivation, and 10% and 8% for the main experiment cultivations on wild type and *mucA-*Δ*algC*, respectively. For mass isotopomers detected by the GC-MS/MS method, these STD values were 10%, 12% and 15%, respectively.

### Computational ^13^C-MFA: modelling, flux estimation and design of informative CLEs

A biochemical reaction network of central carbon metabolism of *P. fluorescens* SBW25 was derived from the genome-scale metabolic model *iSB1139* reconstructed by Borgos et al. [5] as well as from the KEGG database (http://www.genome.jp/kegg). Construction of our model was guided by 1) representing degradation pathways by effluxes from the system and excluding low-abundance biomass components (see Additional file 1: Tables S.1.2 and S.1.4); 2) pooling metabolites due to limited measurement information, e.g., 1,3-bisphosphoglycerate, 2-phosphoglycerate and 3-phosphoglycerate, which are represented by one lumped pool 23PG while preserving the carbon atom transitions; and 3) aggregating parallel fluxes, such as isoenzymes. Like other *Pseudomonas* strains, *P. fluorescens* does not possess a phosphofructokinase enzyme for conversion of *F6P* to *FBP*; thus, only the gluconeogenetic conversion of *FBP* to *F6P* is included in the model (EC 3.1.3.11).

The model of *P. fluorescens* central carbon metabolism consists of routes for fructose uptake (Carbon Uptake), EMP, EDP, PPP, TCA cycle including the *GLX* shunt, the ANA section, as well as the biomass equation, which defines the net fluxes of metabolites into amino acid and fatty acid synthesis. To formulate the latter effluxes, the biomass composition for *P. putida* devised by Nogales et al. [41] was adopted and integrated as a set of constraints for the fluxes into building block synthesis (Additional file 1). In total, the model contains 66 metabolites and 116 reactions (18 thereof reversible), each supplemented with carbon atom transitions (Additional file 1). The model has 29 degrees of freedom (13 net and 15 exchange fluxes) to be estimated from the measured datasets generated for the *P. fluorescens* wild type and the *mucA-*Δ*algC* mutant strain.

The generation of isotopomer balances, the simulation of measurements, the experimental mixture design, the flux estimation (minimisation of a weighted least squares objective repeated 100 times with randomly sampled starting values combined with a globalised optimisation strategy to detect multiple equally good but essentially different flux solutions), and the statistical assessment of resulting flux confidence intervals were performed using the software tool 13CFLUX2 [12]. For further details of the computational ^13^C-MFA protocols, we refer to classical review papers, e.g., [42]. Finally, the resulting flux estimations are represented in the context of the metabolic network model using the visualisation software Omix [43].

An optimal experimental design (OED) study to determine an information-optimised fructose label mixture was performed based on the fluxes estimated from the pre-CLE with *P. fluorescens* SBW25 wild type. The main CLEs for the wild type and the *mucA-*Δ*algC* strain were performed using the calculated OED mixture consisting of 60.08% 1-^13^C-, 39.92% U-^13^C-fructose isotopomers. When inspecting the data thoroughly with the isotopomer network at hand, some peak measurements have to be excluded due to low peak quality. Specifically, we used the following procedure to achieve an acceptable fit: 1) we assumed a minimum STD of 1% to account for the noise of measurements close to the detection limit of the MS instruments, 2) we obtained the best fit by minimising the difference between simulated and measured data, and 3) we excluded those measurements with low abundance that have extraordinary large discrepancies (values for experimental and simulated measurements are found in Additional file 6). Optimisation runs provided a unique optimum for each dataset and flux estimation. For the best-fitting flux maps, the statistical analyses were performed with constraining several main exchange fluxes (as indicated by the absence of uncertainties for fluxes in Additional file 2.

## Additional files

Additional file 1:
**Metabolic network model of central carbon conversion routes of** ***Pseudomonas fluorescens*** **SBW25** **(Figure S.1.1).** Metabolic network model of the central carbon metabolism of *P. fluorescens* SBW25 used for ^13^C metabolic flux analysis **(Table S.1.2)**. Biomass equation for P. putida from Nogales, Palsson et al. [41] used in the model of P. fluorescens SBW25 central carbon metabolism **(Table S1.3)**. Constraint from biomass equation for *P. putida* from Nogales, Palsson et al. [41] used in the model of *P. fluorescens* SBW25 central carbon metabolism based on Additional file 1: Table S1.3 **(Table S.1.4)**. List of main central carbon metabolism metabolites of the *P. fluorescens* SBW25 **(Table S.1.5)**. Additional file 2:
**The complete flux distributions (net and exchange fluxes) and confidence intervals for** ***P. fluorescens*** **SBW25 wild type strain** **(Table S.2.1)** **and** ***mucA- ΔalgC*** **strain (Table S.2.2).** Relative net fluxes for the *mucA-* Δ*algC* strain to the wild-type strains on a logarithmic scale (**Figure S.2.3** (a)) and scaled by fructose uptake (**Figure S.2.3** (b)) on a logarithmic scale. Reactions involving ATP and producing reducing power (NADH/NADPH) by central carbon metabolism of P. fluorescens SBW25 wild type (blue) and the mucA-ΔalgC strain (red) are presented in **Figure S.2.4**. Additional file 3:
**Figure S.3 shows the time profile of the** ^**13**^ **C-labelled fructose** ***P. fluorescens algC*** **-**Δ***mucA*** **double knock out cultivation.**
Additional file 4:
**Mass spectrometry settings for the LC-MS/MS** **(Table S.4.1)** **and GC-MS/MS** **(Table S.4.2)** **MRM acquisition methods and the model format of the measurements (last column).**
Additional file 5:
**Mass isotopomer distributions detected by LC-MS/MS (** **Table S.5.1** **) and GS-MS/MS (** **Table S.5.2** **) for three** ***P. fluorescens*** **cultivations with labeled fructose isotopomer mixtures.**
Additional file 6:
**Table S.6.1 presents values of simulated measurements for the main CLEs.**
