# Supplementary material for: Fluxome study of Pseudomonas fluorescens reveals major reorganisation of carbon flux through central metabolic pathways in response to inactivation of the anti-sigma factor MucA
Source: BMC Syst Biol. 2015 Feb 18;9:6. doi: 10.1186/s12918-015-0148-0 (PMC4351692; doi:10.1186/s12918-015-0148-0)
Supplement: Additional file 1: — Metabolic network model of central carbon conversion routes of Pseudomonas fluorescens SBW25 (Figure S.1.1). Metabolic network model of the central carbon metabolism of P. fluorescens SBW25 used for 13C metabolic flux analysis (Table S.1.2). Biomass equation for P. putida from Nogales, Palsson et al. [41] used in the model of P. fluorescens SBW25 central carbon metabolism (Table S1.3). Constraint from biomass equation for P. putida from Nogales, Palsson et al. [41] used in the model of P. fluorescens SBW25 central carbon metabolism based on Additional file 1: Table S1.3 (Table S.1.4). List of main central carbon metabolism metabolites of the P. fluorescens SBW25 (Table S.1.5). [file 12918_2015_148_MOESM1_ESM.docx]

**Supplementary Figure S.1.1.** Metabolic network model of central carbon conversion routes of *Pseudomonas fluorescens* SBW25. Colors indicate pathway classification: EMP – Embden-Meyerhof-Parnas  pathway – green, PPP – Pentose Phosphate pathway – grey, EDP – Entner-Doudoroff pathway – red, ANA – anaplerotic section - plum, TCA – tricarboxylic acid cycle – blue, C – carbon uptake – white, BS/BM – amino acid biosynthesis and biomass production – brown. The reactions and metabolites are listed in Supplementary Tables S1.2. and S1.5.


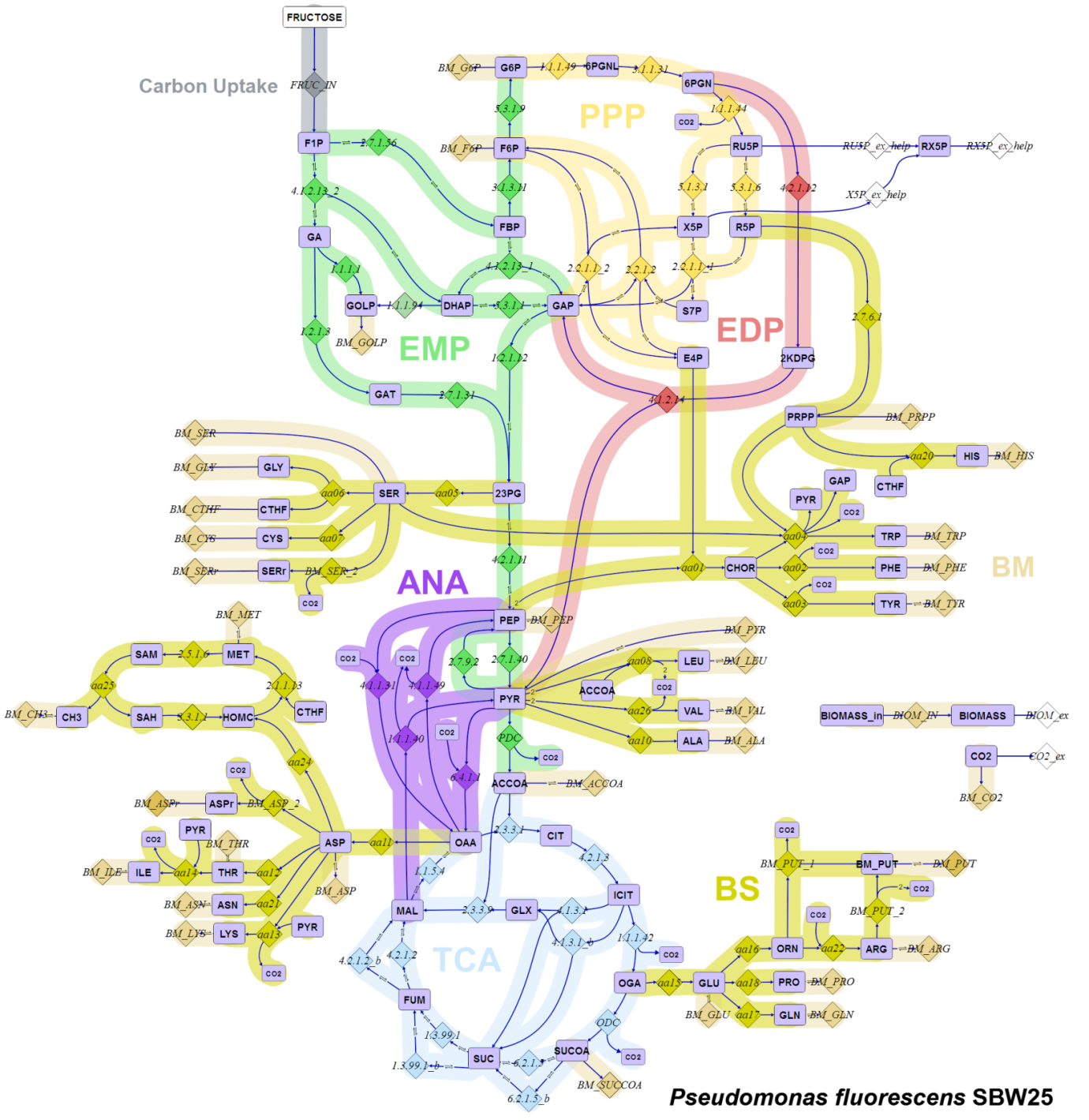


**Supplementary Table S.1.2.** Metabolic network model of the central carbon metabolism of *P. fluorescens* SBW25 used for ^13^C metabolic flux analysis (s.a. Supplementary Figure S.1.1). Linear reaction sequences and biomass synthesis are condensed for simplification. Irreversibility assumptions are derived from thermodynamic considerations and represented by corresponding arrow directions (→ – unidirectional, ↔ – bidirectional). Carbon atom transitions indicating the fate of each carbon atom of each reaction are given in brackets using the letter notation established in Wiechert, W. et al. (1999) Bidirectional reaction steps in metabolic networks: III. Explicit solution and analysis of isotopomer labeling systems. Biotechnol Bioeng, 66, 69–85. EC numbers are used as far as available. For long names of metabolites see Supplementary Table S.1.5. Reactions are assigned to sub-systems: EMP – Embden-Meyerhof-Parnas  pathway, PPP – Pentose Phosphate pathway, EDP – Entner-Doudoroff pathway, ANA – anaplerotic section, TCA – tricarboxylic acid cycle, BS – amino acid biosynthesis, and BM – biomass formation.

| **Reaction** | **Stoichiometry and C-atom transitions** | **Sub-system** |
| --- | --- | --- |
| 1.1.1.1 | GA(#ABC) → GOLP(#BAC) | EDP |
| 1.1.1.40 | MAL(#ABCD) → PYR(#ABD) + CO2(#C) | ANA |
| 1.1.1.42 | ICIT(#ABCDEF) → OGA(#BADCF) + CO2(#E) | TCA |
| 1.1.1.44 | 6PGN(#ABCDEF) → RU5P(#EADBC) + CO2(#F) | PPP |
| 1.1.1.49 | G6P(#ABCDEF) → 6PGNL(#ABCDEF) | PPP |
| 1.1.1.94 | DHAP(#ABC) → GOLP(#ABC) | EMP |
| 1.1.5.4 | MAL(#ABCD) ↔ OAA(#ABCD) | TCA |
| 1.2.1.12 | GAP(#ABC) ↔ 23PG(#BCA) | EMP |
| 1.2.1.3 | GA(#ABC) → GAT(#BCA) | EMP |
| 1.3.99.1 | SUC(#ABCD) ↔ FUM(#ABCD) | TCA |
| 1.3.99.1_b | SUC(#ABCD) ↔ FUM(#BADC) | TCA |
| 2.2.1.1_1 | X5P(#ABCDE) + R5P(#FGHIJ) ↔ S7P(#AFCGJHI) + GAP(#EBD) | PPP |
| 2.2.1.1_2 | F6P(#ABCDEF) + GAP(#GHI) ↔ E4P(#EADC) + X5P(#BHFIG) | PPP |
| 2.2.1.2 | GAP(#ABC) + S7P(#DEFGHIJ) ↔ F6P(#BDCAHF) + E4P(#JEIG) | PPP |
| 2.3.3.1 | OAA(#ABCD) + ACCOA(#EF) → CIT(#AECFDB) | TCA |
| 2.3.3.9 | ACCOA(#AB) + GLX(#CD) → MAL(#ACBD) | TCA |
| 2.7.1.31 | GAT(#ABC) → 23PG(#ABC) | EMP |
| 2.7.1.40 | PEP(#ABC) → PYR(#ABC) | EMP |
| 2.7.1.56 | F1P(#ABCDEF) ↔ FBP(#ABCDEF) | EMP |
| 2.7.9.2 | PYR(#ABC) → PEP(#ABC) | EMP |
| 3.1.1.31 | 6PGNL(#ABCDEF) → 6PGN(#ABCDEF) | PPP |
| 3.1.3.11 | FBP(#ABCDEF) → F6P(#ABCDEF) | EMP |
| 4.1.1.31 | PEP(#ABC) + CO2(#D) → OAA(#ABDC) | ANA |
| 4.1.1.49 | OAA(#ABCD) → CO2(#C) + PEP(#ABD) | ANA |
| 4.1.2.13_1 | FBP(#ABCDEF) ↔ DHAP(#EBF) + GAP(#DAC) | EMP |
| 4.1.2.13_2 | F1P(#ABCDEF) ↔ DHAP(#EBF) + GA(#DAC) | EMP |
| 4.1.2.14 | 2KDPG(#ABCDEF) → GAP(#CBE) + PYR(#ADF) | EDP |
| 4.1.3.1 | ICIT(#ABCDEF) → SUC(#ABCE) + GLX(#DF) | TCA |
| 4.1.3.1_b | ICIT(#ABCDEF) → SUC(#BAEC) + GLX(#DF) | TCA |
| 4.2.1.11 | 23PG(#ABC) ↔ PEP(#ABC) | EMP |
| 4.2.1.12 | 6PGN(#ABCDEF) → 2KDPG(#DACEBF) | EDP |
| 4.2.1.2 | FUM(#ABCD) ↔ MAL(#BADC) | TCA |
| 4.2.1.2_b | FUM(#ABCD) ↔ MAL(#ABCD) | TCA |
| 4.2.1.3 | CIT(#ABCDEF) → ICIT(#BFDAEC) | TCA |
| 5.1.3.1 | RU5P(#ABCDE) ↔ X5P(#ABCDE) | PPP |
| 5.3.1.1 | DHAP(#ABC) ↔ GAP(#ABC) | EMP |
| 5.3.1.6 | RU5P(#ABCDE) ↔ R5P(#BDECA) | PPP |
| 5.3.1.9 | F6P(#ABCDEF) → G6P(#ACDEFB) | EMP |
| 6.2.1.5 | SUCCOA(#ABCD) ↔ SUC(#BADC) | TCA |
| 6.2.1.5_b | SUCCOA(#ABCD) ↔ SUC(#ABCD) | TCA |
| 6.4.1.1 | PYR(#ABC) + CO2(#D) → OAA(#ABDC) | ANA |
| ODC | OGA(#ABCDE) → SUCCOA(#BADC) + CO2(#E) | TCA |
| PDC | PYR(#ABC) → ACCOA(#AB) + CO2(#C) | EMP |
| // Amino acid biosynthesis (simplified) | |  |
| 2.1.1.13 | CTHF(#A) + HOMC(#BCDE) → MET(#ABCDE) | BS |
| 2.5.1.6 | MET(#ABCDE) → SAM(#ABCDE) | BS |
| 2.7.6.1 | R5P(#ABCDE) → PRPP(#ABCDE) | BS |
| 3.3.1.1 | SAH(#ABCD) → HOMC(#ABCD) | BS |
| aa01 | E4P(#ABCD) + PEP(#EFG) + PEP(#HIJ) → CHOR(#HBDEIFCAJG) | BS |
| aa02 | CHOR(#ABCDEFGHIJ) → PHE(#GHCDBAFEI) + CO2(#J) | BS |
| aa03 | CHOR(#ABCDEFGHIJ) → TYR(#DBHCAFGEI) + CO2(#J) | BS |
| aa04 | CHOR(#ABCDEFGHIJ) + PRPP(#KLMNO) + SER(#PQR) → PYR(#AEI) + TRP(#HGDCPONFQBR) + CO2(#J) + GAP(#MKL) | BS |
| aa05 | 23PG(#ABC) → SER(#ABC) | BS |
| aa06 | SER(#ABC) → GLY(#BC) + CTHF(#A) | BS |
| aa07 | SER(#ABC) → CYS(#ABC) | BS |
| aa08 | PYR(#ABC) + PYR(#DEF) + ACCOA(#GH) → LEU(#DAEBGH) + CO2(#F) + CO2(#C) | BS |
| aa10 | PYR(#ABC) → ALA(#ABC) | BS |
| aa11 | OAA(#ABCD) → ASP(#ABCD) | BS |
| aa12 | ASP(#ABCD) → THR(#CABD) | BS |
| aa13 | ASP(#ABCD) + PYR(#EFG) → LYS(#CEAFBD) + CO2(#G) | BS |
| aa14 | THR(#ABCD) + PYR(#EFG) → ILE(#AEBFCD) + CO2(#G) | BS |
| aa15 | OGA(#ABCDE) → GLU(#ABCDE) | BS |
| aa16 | GLU(#ABCDE) → ORN(#BADCE) | BS |
| aa17 | GLU(#ABCDE) → GLN(#ABCDE) | BS |
| aa18 | GLU(#ABCDE) → PRO(#BADCE) | BS |
| aa20 | PRPP(#ABCDE) + CTHF(#F) → HIS(#CEFDBA) | BS |
| aa21 | ASP(#ABCD) → ASN(#ABCD) | BS |
| aa22 | CO2(#A) + ORN(#BCDEF) → ARG(#BCDEFA) | BS |
| aa24 | ASP(#ABCD) → HOMC(#ACBD) | BS |
| aa25 | SAM(#ABCDE) → SAH(#BCDE) + CH3(#A) | BS |
| aa26 | PYR(#ABC) + PYR(#DEF) → VAL(#DAEBC) + CO2(#F) | BS |
| BM_ASP_2 | ASP(#ABCD) → ASPr(#ABC) + CO2(#D) | BS |
| BM_PUT_1 | ORN(#ABCDE) → PUT(#BADC) + CO2(#E) | BS |
| BM_PUT_2 | ARG(#ABCDEF) → CO2(#F) + PUT(#ABCD) + CO2(#E) | BS |
| BM_SER_2 | SER(#ABC) → SERr(#AB) + CO2(#C) | BS |
| // Biomass forming reactions | |  |
| BM_ACCOA | ACCOA(#AB) → | BM |
| BM_ALA | ALA(#ABC) → | v |
| BM_ARG | ARG(#ABCDEF) → | BM |
| BM_ASN | ASN(#ABCD) → | BM |
| BM_ASP | ASP(#ABCD) → | BM |
| BM_ASPr | ASPr(#ABC) → | BM |
| BM_CH3 | CH3(#A) → | BM |
| BM_CO2 | CO2(#A) → | BM |
| BM_CTHF | CTHF(#A) → | BM |
| BM_CYS | CYS(#ABC) → | BM |
| BM_F6P | F6P(#ABCDEF) → | BM |
| BM_G6P | G6P(#ABCDEF) → | BM |
| BM_GLN | GLN(#ABCDE) → | BM |
| BM_GLU | GLU(#ABCDE) → | BM |
| BM_GLY | GLY(#AB) → | BM |
| BM_GOLP | GOLP(#ABC) → | BM |
| BM_HIS | HIS(#ABCDEF) → | BM |
| BM_ILE | ILE(#ABCDEF) → | BM |
| BM_LEU | LEU(#ABCDEF) → | BM |
| BM_LYS | LYS(#ABCDEF) → | BM |
| BM_MET | MET(#ABCDE) → | BM |
| BM_PEP | PEP(#ABC) → | BM |
| BM_PHE | PHE(#ABCDEFGHI) → | BM |
| BM_PRO | PRO(#ABCDE) → | BM |
| BM_PRPP | PRPP(#ABCDE) → | BM |
| BM_PUT | PUT(#ABCD) → | BM |
| BM_PYR | PYR(#ABC) → | BM |
| BM_SER | SER(#ABC) → | BM |
| BM_SERr_2 | SERr(#AB) → | BM |
| BM_SUCCOA | SUCCOA(#ABCD) → | BM |
| BM_THR | THR(#ABCD) → | BM |
| BM_TRP | TRP(#ABCDEFGHIJK) → | BM |
| BM_TYR | TYR(#ABCDEFGHI) → | BM |
| BM_VAL | VAL(#ABCDE) → | BM |
| BM_TRP | TRP(#ABCDEFGHIJK) → | BM |
| // Carbon exchange reactions | |  |
| FRUC_IN | FRUCTOSE(#ABCDEF) → F1P(#ABCDEF) | Carbon uptake |
| BIOMASS_IN | BIOMASS in(#) → BIOMASS(#) | Carbon Exchange |
| BIOM_ex | BIOMASS(#) → | Carbon Exchange |
| CO2_ex | CO2(#A) → | Carbon Exchange |
| RX5P_ex_help | RX5P(#ABCDE) → | Auxiliary reaction  (#overlapping measured spectra) |
| RU5P_ex_help | RU5P(#ABCDE) → RX5P(#BDECA) | Auxiliary reaction  (#overlapping measured spectra) |
| X5P_ex_help | X5P(#ABCDE) → RX5P(#BDECA) | Auxiliary reaction  (#overlapping measured spectra) |
| Gly_ex | GLY(#AB) → | Carbon exchange to balance glycine formation |

**Supplementary Table S1.3.** Biomass equation for *P. putida* from Nogales, Palsson et al. (2008) used in the model of *P. fluorescens* SBW25 central carbon metabolism. For compounds present in the model the biomass equation terms were incorporated directly as a drain from the metabolite. For the remaining biomass equation terms the educts of the summary equation were added as a drain. The resulting constraints that were used in the model can be found in Supplementary Table S.1.4. Coenzyme A (0.000006 mmol/gDW), FAD (0.00001 mmol/gDW), NAD^+^ (0.00125 mmol/gDW), NADH (0.00005 mmol/gDW), NADP^+^ (0.00013 mmol/gDW), NADPH (0.0004 mmol/gDW) Heme O (0.0005 mmol/gDW), and Siroheme (0.0005 mmol/gDW) present in the biomass equation for *P. putida* are omitted because of their low contribution and/or to reduce complexity of the model. pg: phosphatidylglycerol; clpn: cardiolipin; cpg: cyclopropane phosphatidylglycerol; pe: phosphatidylethanolamine; cpe: cyclopropane phosphatidylethanolamine.

| Compound | Amount [mmol/gDW] | Summary of equations for compound synthesis  (carbon transferring species only) |
| --- | --- | --- |
| SUCCOA | 0.000003 |  |
| ACCOA | 0.00005 |  |
| TRP | 0.054 |  |
| CYS | 0.087 |  |
| HIS | 0.09 |  |
| TYR | 0.131 |  |
| MET | 0.146 |  |
| PHE | 0.176 |  |
| SER | 0.205 |  |
| PRO | 0.21 |  |
| ASN | 0.229 |  |
| ASP | 0.229 |  |
| THR | 0.241 |  |
| GLN | 0.25 |  |
| GLU | 0.25 |  |
| ILE | 0.276 |  |
| ARG | 0.281 |  |
| LYS | 0.326 |  |
| VAL | 0.402 |  |
| LEU | 0.428 |  |
| ALA | 0.488 |  |
| GLY | 0.582 |  |
| 5-methyl-THF | 0.05 |  |
| UDP-D-glucose | 0.003 | G6P + CO2 +ASP +PRPP → CO2 + UDP-D-glucose |
| peptidoglycan | 0.028 | 2 F6P + 2 ACCOA + PEP + 3 ALA + GLU + ASP + PYR → peptidoglycan |
| putrescine | 0.035 | ARG → 2 CO2 + PUT  ORN → CO2 + PUT |
| pg120 | 0.0005 | 12 ACCOA + 2 GOLP → pg120 |
| pg160 | 0.0005 | 16 ACCOA + 2 GOLP → pg160 |
| pg180 | 0.0005 | 18 ACCOA + 2 GOLP → pg180 |
| clpn120 | 0.0005 | 24 ACCOA + 3 GOLP → clpn120 |
| clpn160 | 0.0005 | 32 ACCOA + 3 GOLP → clpn160 |
| clpn161 | 0.0005 | 32 ACCOA + 3 GOLP → clpn161 |
| clpn180 | 0.0005 | 36 ACCOA + 3 GOLP → clpn180 |
| clpn181 | 0.0005 | 36 ACCOA + 3 GOLP → clpn181 |
| cpg160 | 0.0005 | 2 SAM + 16 ACCOA + 2 GOLP → 2 SAH + cpg160 |
| cpg180 | 0.0005 | 2 SAM + 18 ACCOA + 2 GOLP → 2 SAH + cpg160 |
| pe120 | 0.0005 | 12 ACCOA + GOLP + SER → CO2 + pe120 |
| pe160 | 0.0005 | 16 ACCOA + GOLP + SER → CO2 + pe160 |
| pe161 | 0.0005 | 16 ACCOA + GOLP + SER → CO2 + pe161 |
| pe180 | 0.0005 | 18 ACCOA + GOLP + SER → CO2 + pe180 |
| pe181 | 0.0005 | 18 ACCOA + GOLP + SER → CO2 + pe181 |
| cpe160 | 0.0005 | 2 SAM + pe161 → 2 SAH + cpe160 |
| cpe180 | 0.0005 | 2 SAM + pe181 → 2 SAH + cpe180 |
| dATP | 0.0247 | PRPP + GLY + 2 THF-COH + HCO3- → dATP + 2 THF |
| dTTP | 0.0247 | CO2 + ASP + PRPP + THF-COH → CO2 + dTTP + THF |
| dCTP | 0.0254 | CO2 + ASP + PRPP → CO2 + dCTP |
| dGTP | 0.0254 | PRPP + GLY + 2 THF-COH + HCO_3_^-^- → dGTP + 2 THF |
| CTP | 0.126 | CO2 + ASP + PRPP → CO2 + CTP |
| UTP | 0.136 | CO2 + ASP + PRPP → CO2 + UTP |
| GTP | 0.203 | PRPP + GLY + 2 THF-COH + HCO_3_^-^ → GTP + 2 THF |
| ATP | 0.171 | PRPP + GLY + 2 THF-COH + HCO_3_^-^ → ATP + 2 THF |
| AMP | 0.001 | PRPP + GLY + 2 THF-COH + HCO_3_^-^→ AMP + 2 THF |

**Supplementary Table S1.4.** Constraint from biomass equation for *P. putida* from Nogales, Palsson et al. (2008) used in the model of *P. fluorescens* SBW25 central carbon metabolism based on Supplementary Table S1.3.

| Flux | Constraint [mmol/gDW] |
| --- | --- |
| BM_SUCCOA | 0.000003 |
| BM_ACCOA | 0.23305 |
| BM_PUT_1+BM_PUT_2 | 0.035 |
| BM_TRP | 0.054 |
| BM_CYS | 0.087 |
| BM_HIS | 0.09 |
| BM_TYR | 0.131 |
| BM_MET | 0.146 |
| BM_PHE | 0.176 |
| BM_SER | 0.205 |
| BM_PRO | 0.21 |
| BM_ASN | 0.229 |
| BM_ASP | 0.257 |
| BM_THR | 0.241 |
| BM_GLN | 0.25 |
| BM_GLU | 0.278 |
| BM_ILE | 0.276 |
| BM_ARG | 0.281 |
| BM_LYS | 0.326 |
| BM_VAL | 0.402 |
| BM_LEU | 0.428 |
| BM_ALA | 0.572 |
| BM_GLY | 1.0071 |
| BM_GOLP | 0.016 |
| BM_CH3 | 0.004 |
| BM_SERr_2 | 0.0035 |
| BM_F6P | 0.056 |
| BM_PEP | 0.028 |
| BM_PYR | 0.028 |
| BM_PRPP | 0.7402 |
| BM_CTHF | 0.9249 |
| BM_CO2 | 0.7402 |
| BM_ASPr | 0.3151 |
| BM_G6P | 0.003 |

**TABLE S.1.5.** List of main central carbon metabolism metabolites of the *P. fluorescens* SBW25.

| **Acronym** | **Long Name** |
| --- | --- |
| 23PG | 2-phosphoglycerate + 3-phosphoglycerate |
| 2KDPG | 2-keto-3-deoxy-6-phospho-gluconate |
| 6PGN | 6-phosphogluconate |
| 6PGNL | 6-phosphogluconolactone |
| ACCOA | acetyl-CoA |
| ADP | adenosine diphosphate |
| ALA | L-alanine |
| ARG | L-arginine |
| AMP | adenosine monophosphate |
| ASN | L-asparagine |
| ASP | L-aspartate |
| ASPr | L-aspartate used for biosynthesis |
| ATP | adenosine triphosphate |
| BIOMASS | biomass |
| BIOMASS_in | biomass input |
| CH3 | methyl/C1 group |
| CHOR | chorismate |
| CIT | citrate |
| CO2 | carbon dioxide |
| CTHF | 5-methyltetrahydrofolate |
| CYS | L-cysteine |
| dATP | deoxyadenosine triphosphate |
| dCTP | deoxycytidine triphosphate |
| dGTP | deoxyguanosine triphosphate |
| DHAP | dihydroxy-acetone-phosphate |
| dTTP | deoxythymidine triphosphate |
| E4P | erythrose-4-phosphate |
| F1P | fructose-1-phosphate |
| F6P | fructose-6-diphosphate |
| FBP | fructose-1,6- bisphosphate |
| FRUCTOSE | fructose (extracellular) |
| FUM | fumarate |
| G6P | glucose 6-phosphate |
| GA | glyceraldehyde |
| GAP | D-glyceraldehyde-3-phosphate |
| GAT | glycerate |
| GLN | L-glutamine |
| GLU | L-glutamate |
| GLX | glyoxylate |
| GLY | glycine |
| GOLP | glycerol-3-phosphate |
| GTP | guanosine triphiphosphate |
| HIS | L-histidine |
| HOMC | L-homocysteine |
| ICIT | isocitrate |
| ILE | L-isoleucine |
| LEU | L-leucine |
| LYS | L-lysine |
| MAL | malate |
| MET | L-methionine |
| NADH | nicotinamide adenine dinucleotide |
| NADPH | nicotinamide adenine dinucleotide phosphate |
| OAA | oxaloacetate |
| OGA | oxoglutarate |
| ORN | L-ornithine |
| PEP | phosphoenolpyruvate |
| PHE | L-phenylalanine |
| PRO | L-proline |
| PRPP | phosphoribosylpyrophosphate |
| PUT | putrescine |
| PYR | pyruvate |
| R5P | D-ribose-5-phosphate |
| RU5P | D-ribulose-5-phosphate |
| RX5P | D-ribulose-5-phosphate + D-xylulose-5-phosphate |
| S7P | D-sedoheptulose-7-phosphate |
| SAH | S-adenosyl-homocysteine |
| SAM | S-adenosyl-L-methionine |
| SER | L-serine |
| SERr | L-serine residue used for biosynthesis |
| SUC | succinate |
| SUCCOA | succinyl-CoA |
| THF | tetrahydrofolate |
| THR | L-threonine |
| TRP | L-tryptophan |
| TTP | thymidine triphosphate |
| TYR | L-tyrosine |
| UTP | uridine triphosphate |
| VAL | L-valine |
| X5P | xylulose-5-phosphate |
