# Supplementary material for: Fluxome study of Pseudomonas fluorescens reveals major reorganisation of carbon flux through central metabolic pathways in response to inactivation of the anti-sigma factor MucA
Source: BMC Syst Biol. 2015 Feb 18;9:6. doi: 10.1186/s12918-015-0148-0 (PMC4351692; doi:10.1186/s12918-015-0148-0)
Supplement: Additional file 2: — The complete flux distributions (net and exchange fluxes) and confidence intervals for P. fluorescens SBW25 wild type strain (Table S.2.1) and mucA- ΔalgC strain (Table S.2.2). Relative net fluxes for the mucA- ΔalgC strain to the wild-type strains on a logarithmic scale (Figure S.2.3 (a)) and scaled by fructose uptake (Figure S.2.3 (b)) on a logarithmic scale. Reactions involving ATP and producing reducing power (NADH/NADPH) by central carbon metabolism of P. fluorescens SBW25 wild type (blue) and the mucA-ΔalgC strain (red) are presented in Figure S.2.4. [file 12918_2015_148_MOESM2_ESM.docx]

**Supplementary Table S.2.1.** The complete flux distributions (net and exchange fluxes) and confidence intervals for *P. fluorescens* SBW25 wild type strain. For reaction specification see Supplementary Table S.1.2.

|  | | **Net** | | **Xch** | **Net STD** | **Xch STD** |
| --- | --- | --- | --- | --- | --- | --- |
|  | [mmol g^-1^_DW_ h^-1^] | | [mmol g^-1^_DW_ h^-1^] | | [mmol g^-1^_DW_ h^-1^] | [mmol g^-1^_DW_ h^-1^] |
| 1.1.1.1 | | 0.0000 | 0.0000 | | 1.0331 | 0.0000 |
| 1.1.1.40 | | 0.8445 | 0.0000 | | 0.2735 | 0.0000 |
| 1.1.1.42 | | 3.1039 | 0.0000 | | 0.2750 | 0.0000 |
| 1.1.1.44 | | 1.1758 | 0.0000 | | 0.1951 | 0.0000 |
| 1.1.1.49 | | 3.0001 | 0.0000 | | 0.2568 | 0.0000 |
| 1.1.1.94 | | 0.0006 | 0.0000 | | 1.0331 | 0.0000 |
| 1.1.5.4 | | 2.2168 | 89.0627 | | 0.2834 | 0.0000 |
| 1.2.1.12 | | 1.6266 | 7254.0542 | | 0.0000 | 0.0000 |
| 1.2.1.3 | | 0.0000 | 0.0000 | | 0.0000 | 0.0000 |
| 1.3.99.1 | | 1.5306 | 49.9996 | | 0.0862 | 0.0000 |
| 1.3.99.1_b | | 1.5306 | 49.9996 | | 0.0862 | 0.0000 |
| 2.1.1.13 | | 0.0061 | 0.0000 | | 0.0006 | 0.0000 |
| 2.2.1.1_1 | | 0.3849 | 15.6321 | | 0.0650 | 0.0000 |
| 2.2.1.1_2 | | -0.3703 | 0.8210 | | 0.0651 | 0.1390 |
| 2.2.1.2 | | 0.3849 | 0.1240 | | 0.0650 | 0.0779 |
| 2.3.3.1 | | 3.1039 | 0.0000 | | 0.1714 | 0.0000 |
| 2.3.3.9 | | 0.0000 | 0.0000 | | 0.1916 | 0.0000 |
| 2.5.1.6 | | 0.0002 | 0.0000 | | 0.0000 | 0.0000 |
| 2.7.1.31 | | 0.0000 | 0.0000 | | 0.0000 | 0.0000 |
| 2.7.1.40 | | 1.1711 | 0.0000 | | 0.2394 | 0.0000 |
| 2.7.1.56 | | 1.9626 | 0.0000 | | 0.9815 | 8.4799 |
| 2.7.6.1 | | 0.0358 | 0.0000 | | 0.0035 | 0.0000 |
| 2.7.9.2 | | 0.0000 | 0.0000 | | 0.2856 | 0.0000 |
| 3.1.1.31 | | 3.0001 | 0.0000 | | 0.2568 | 0.0000 |
| 3.1.3.11 | | 2.2473 | 0.0000 | | 0.1787 | 0.0000 |
| 3.3.1.1 | | 0.0002 | 0.0000 | | 0.0000 | 0.0000 |
| 4.1.1.31 | | 0.4070 | 0.0000 | | 0.2122 | 0.0000 |
| 4.1.1.49 | | 0.0432 | 0.0000 | | 0.1580 | 0.0000 |
| 4.1.2.13 | | -0.2848 | 0.0086 | | 1.0898 | 0.0000 |
| 4.1.2.13_2 | | 0.0000 | 0.0148 | | 1.0331 | 1.0898 |
| 4.1.2.14 | | 1.8243 | 0.0000 | | 0.1663 | 0.0000 |
| 4.1.3.1 | | 0.0000 | 0.0000 | | 0.0958 | 0.0000 |
| 4.1.3.1.b | | 0.0000 | 0.0000 | | 0.0958 | 0.0000 |
| 4.2.1.11 | | 1.5653 | 5766.8728 | | 0.0061 | 0.0000 |
| 4.2.1.12 | | 1.8243 | 0.0000 | | 0.1663 | 0.0000 |
| 4.2.1.2 | | 1.5306 | 0.3482 | | 0.0862 | 0.7817 |
| 4.2.1.2_b | | 1.5306 | 0.3482 | | 0.0862 | 0.7817 |
| 4.2.1.3 | | 3.1039 | 0.0000 | | 0.1714 | 0.0000 |
| 5.1.3.1 | | 0.7552 | 11.5187 | | 0.1301 | 0.0000 |
| 5.3.1.1 | | -0.2854 | 0.0000 | | 0.9812 | 0.6873 |
|  | | **Net** | | **Xch** | **Net STD** | **Xch STD** |
|  | [mmol g^-1^_DW_ h^-1^] | | [mmol g^-1^_DW_ h^-1^] | | [mmol g^-1^_DW_ h^-1^] | [mmol g^-1^_DW_ h^-1^] |
| 5.3.1.6 | | 0.4207 | 0.0000 | | 0.0651 | 0.3149 |
| 5.3.1.9 | | 3.0002 | 0.0000 | | 0.2568 | 0.0000 |
| 6.2.1.5 | | 1.5306 | 49.9996 | | 0.1380 | 0.0000 |
| 6.2.1.5_b | | 1.5306 | 49.9996 | | 0.1380 | 0.0000 |
| 6.4.1.1 | | 0.5957 | 0.0000 | | 0.3727 | 0.0000 |
| aa01b | | 0.0146 | 0.0000 | | 0.0014 | 0.0000 |
| aa02 | | 0.0071 | 0.0000 | | 0.0007 | 0.0000 |
| aa03 | | 0.0053 | 0.0000 | | 0.0005 | 0.0000 |
| aa04 | | 0.0022 | 0.0000 | | 0.0002 | 0.0000 |
| aa05 | | 0.0613 | 0.0000 | | 0.0061 | 0.0000 |
| aa06 | | 0.0471 | 0.0000 | | 0.0047 | 0.0000 |
| aa07 | | 0.0035 | 0.0000 | | 0.0003 | 0.0000 |
| aa08 | | 0.0173 | 0.0000 | | 0.0017 | 0.0000 |
| aa10 | | 0.0231 | 0.0000 | | 0.0023 | 0.0000 |
| aa11 | | 0.0724 | 0.0000 | | 0.0072 | 0.0000 |
| aa12 | | 0.0209 | 0.0000 | | 0.0021 | 0.0000 |
| aa13 | | 0.0132 | 0.0000 | | 0.0013 | 0.0000 |
| aa14 | | 0.0112 | 0.0000 | | 0.0011 | 0.0000 |
| aa15 | | 0.0426 | 0.0000 | | 0.0042 | 0.0000 |
| aa16 | | 0.0128 | 0.0000 | | 0.0013 | 0.0000 |
| aa17 | | 0.0101 | 0.0000 | | 0.0010 | 0.0000 |
| aa18 | | 0.0085 | 0.0000 | | 0.0008 | 0.0000 |
| aa20 | | 0.0036 | 0.0000 | | 0.0004 | 0.0000 |
| aa21 | | 0.0093 | 0.0000 | | 0.0009 | 0.0000 |
| aa22 | | 0.0114 | 0.0000 | | 0.0011 | 0.0000 |
| aa24 | | 0.0059 | 0.0000 | | 0.0006 | 0.0000 |
| aa25 | | 0.0002 | 0.0000 | | 0.0000 | 0.0000 |
| aa26 | | 0.0163 | 0.0000 | | 0.0016 | 0.0000 |
| Asp_res_ex | | 0.0127 | 0.0000 | | 0.0013 | 0.0000 |
| BIOM_ex | | 0.0404 | 0.0000 | | 0.0040 | 0.0000 |
| BIOM_IN | | 0.0404 | 0.0000 | | 0.0040 | 0.0000 |
| BM_2_Asp | | 0.0127 | 0.0000 | | 0.0013 | 0.0000 |
| BM_2_Ser | | 0.0001 | 0.0000 | | 0.0000 | 0.0000 |
| BM_2_Ser_res | | 0.0001 | 0.0000 | | 0.0000 | 0.0000 |
| BM_Acetyl_CoA | | 0.0094 | 0.0000 | | 0.0009 | 0.0000 |
| BM_Ala | | 0.0231 | 0.0000 | | 0.0023 | 0.0000 |
| BM_Arg | | 0.0114 | 0.0000 | | 0.0011 | 0.0000 |
| BM_Asn | | 0.0093 | 0.0000 | | 0.0009 | 0.0000 |
| BM_Asp | | 0.0104 | 0.0000 | | 0.0010 | 0.0000 |
| BM_C_THF | | 0.0374 | 0.0000 | | 0.0037 | 0.0000 |
| BM_CH3 | | 0.0002 | 0.0000 | | 0.0000 | 0.0000 |
| BM_CO2 | | 0.0299 | 0.0000 | | 0.0030 | 0.0000 |
| BM_Cys | | 0.0035 | 0.0000 | | 0.0003 | 0.0000 |
|  | | **Net** | | **Xch** | **Net STD** | **Xch STD** |
|  | [mmol g^-1^_DW_ h^-1^] | | [mmol g^-1^_DW_ h^-1^] | | [mmol g^-1^_DW_ h^-1^] | [mmol g^-1^_DW_ h^-1^] |
| BM_F6P | | 0.0023 | 0.0000 | | 0.0002 | 0.0000 |
| BM_G6P | | 0.0001 | 0.0000 | | 0.0000 | 0.0000 |
| BM_Gln | | 0.0101 | 0.0000 | | 0.0010 | 0.0000 |
| BM_Glu | | 0.0112 | 0.0000 | | 0.0011 | 0.0000 |
| BM_Gly | | 0.0407 | 0.0000 | | 0.0040 | 0.0000 |
| BM_Gol3P | | 0.0006 | 0.0000 | | 0.0001 | 0.0000 |
| BM_His | | 0.0036 | 0.0000 | | 0.0004 | 0.0000 |
| BM_Ile | | 0.0112 | 0.0000 | | 0.0011 | 0.0000 |
| BM_Leu | | 0.0173 | 0.0000 | | 0.0017 | 0.0000 |
| BM_Lys | | 0.0132 | 0.0000 | | 0.0013 | 0.0000 |
| BM_Met | | 0.0059 | 0.0000 | | 0.0006 | 0.0000 |
| BM_PEP | | 0.0157 | 0.0000 | | 0.0001 | 0.0000 |
| BM_Phe | | 0.0071 | 0.0000 | | 0.0007 | 0.0000 |
| BM_Pro | | 0.0085 | 0.0000 | | 0.0008 | 0.0000 |
| BM_PRPP | | 0.0299 | 0.0000 | | 0.0030 | 0.0000 |
| BM_Put_1 | | 0.0014 | 0.0000 | | 0.0001 | 0.0000 |
| BM_Put_2 | | 0.0000 | 0.0000 | | 0.0000 | 0.0000 |
| BM_Put_out | | 0.0014 | 0.0000 | | 0.0001 | 0.0000 |
| BM_Pyr | | 0.0011 | 0.0000 | | 0.0001 | 0.0000 |
| BM_Ser | | 0.0083 | 0.0000 | | 0.0008 | 0.0000 |
| BM_Suc_CoA | | 0.0000 | 0.0000 | | 0.0000 | 0.0000 |
| BM_Thr | | 0.0097 | 0.0000 | | 0.0010 | 0.0000 |
| BM_Trp | | 0.0022 | 0.0000 | | 0.0002 | 0.0000 |
| BM_Tyr | | 0.0053 | 0.0000 | | 0.0005 | 0.0000 |
| BM_Val | | 0.0163 | 0.0000 | | 0.0016 | 0.0000 |
| CO2_EX | | 10.4195 | 0.0000 | | 0.5518 | 0.0000 |
| FRUC_IN | | 1.9626 | 0.0000 | | 0.0893 | 0.0000 |
| Gly_ex | | 0.0064 | 0.0000 | | 0.0006 | 0.0000 |
| ODC | | 3.0613 | 0.0000 | | 0.2760 | 0.0000 |
| PDC | | 3.1307 | 0.0000 | | 0.2376 | 0.0000 |
| Ru5P_ex_help | | 0.0000 | 0.0000 | | 0.0000 | 0.0000 |
| Ru5P_X5P_ex_help | | 0.0000 | 0.0000 | | 0.0000 | 0.0000 |
| X5P_ex_help | | 0.0000 | 0.0000 | | 0.0000 | 0.0000 |

**Supplementary Table S.2.2.** The complete flux distributions (net and exchange fluxes) and confidence intervals for *P. fluorescens mucA- ΔalgC* strain. For reaction specification see Supplementary Table S.1.2.

|  | **Net** | **Xch** | **Net STD** | **Xch STD** |
| --- | --- | --- | --- | --- |
|  | [mmol g^-1^_DW_ h^-1^] | [mmol g^-1^_DW_ h^-1^] | [mmol g^-1^_DW_ h^-1^] | [mmol g^-1^_DW_ h^-1^] |
| 1.1.1.1 | 0.0000 | 0.0000 | 0.4292 | 0.0000 |
| 1.1.1.40 | 0.1972 | 0.0000 | 0.4291 | 0.0000 |
| 1.1.1.42 | 1.1768 | 0.0000 | 0.2044 | 0.0000 |
| 1.1.1.44 | 1.6172 | 0.0000 | 0.3178 | 0.0000 |
| 1.1.1.49 | 2.2779 | 0.0000 | 0.2942 | 0.0000 |
| 1.1.1.94 | 0.0006 | 0.0000 | 0.4292 | 0.0000 |
| 1.1.5.4 | 1.6056 | 16.0596 | 0.4092 | 0.0000 |
| 1.2.1.12 | 1.1820 | 113.9805 | 0.0000 | 0.0000 |
| 1.2.1.3 | 0.0016 | 0.0000 | 0.0000 | 0.0000 |
| 1.3.99.1 | 0.7346 | 50.0982 | 0.0469 | 0.0000 |
| 1.3.99.1_b | 0.7346 | 50.0982 | 0.0469 | 0.0000 |
| 2.1.1.13 | 0.0058 | 0.0000 | 0.0006 | 0.0000 |
| 2.2.1.1_1 | 0.5323 | 0.0000 | 0.1060 | 0.2381 |
| 2.2.1.1_2 | -0.5182 | 1.6145 | 0.1060 | 0.4917 |
| 2.2.1.2 | 0.5323 | 27.8688 | 0.1060 | 0.0000 |
| 2.3.3.1 | 1.5103 | 0.0000 | 0.0921 | 0.0000 |
| 2.3.3.9 | 0.3335 | 0.0000 | 0.1567 | 0.0000 |
| 2.5.1.6 | 0.0002 | 0.0000 | 0.0000 | 0.0000 |
| 2.7.1.31 | 0.0016 | 0.0000 | 0.0000 | 0.0000 |
| 2.7.1.40 | 2.2491 | 0.0000 | 1.0039 | 0.0000 |
| 2.7.1.56 | 1.2297 | 55.6353 | 0.3900 | 0.0000 |
| 2.7.6.1 | 0.0344 | 0.0000 | 0.0035 | 0.0000 |
| 2.7.9.2 | 1.1282 | 0.0000 | 1.4301 | 0.0000 |
| 3.1.1.31 | 2.2779 | 0.0000 | 0.2942 | 0.0000 |
| 3.1.3.11 | 1.2297 | 0.0000 | 0.1056 | 0.0000 |
| 3.3.1.1 | 0.0002 | 0.0000 | 0.0000 | 0.0000 |
| 4.1.1.31 | 0.8583 | 0.0000 | 0.3828 | 0.0000 |
| 4.1.1.49 | 0.8839 | 0.0000 | 0.5000 | 0.0000 |
| 4.1.2.13 | 0.0000 | 0.0000 | 0.4713 | 0.3350 |
| 4.1.2.13_2 | 0.0016 | 0.0534 | 0.4292 | 0.3040 |
| 4.1.2.14 | 0.6607 | 0.0000 | 0.0845 | 0.0000 |
| 4.1.3.1 | 0.1668 | 0.0000 | 0.0783 | 0.0000 |
| 4.1.3.1.b | 0.1668 | 0.0000 | 0.0783 | 0.0000 |
| 4.2.1.11 | 1.1246 | 0.4401 | 0.0060 | 0.1777 |
| 4.2.1.12 | 0.6607 | 0.0000 | 0.0845 | 0.0000 |
| 4.2.1.2 | 0.7346 | 2.1807 | 0.0469 | 0.0000 |
| 4.2.1.2_b | 0.7346 | 2.1807 | 0.0469 | 0.0000 |
| 4.2.1.3 | 1.5103 | 0.0000 | 0.0921 | 0.0000 |
| 5.1.3.1 | 1.0505 | 1.3300 | 0.2120 | 0.9068 |
| 5.3.1.1 | 0.0010 | 0.4630 | 0.3898 | 0.5513 |
|  | **Net** | **Xch** | **Net STD** | **Xch STD** |
|  | [mmol g^-1^_DW_ h^-1^] | [mmol g^-1^_DW_ h^-1^] | [mmol g^-1^_DW_ h^-1^] | [mmol g^-1^_DW_ h^-1^] |
| 5.3.1.6 | 0.5667 | 205.9212 | 0.1059 | 0.0000 |
| 5.3.1.9 | 2.2780 | 0.0000 | 0.2942 | 0.0000 |
| 6.2.1.5 | 0.5679 | 50.3902 | 0.1029 | 0.0000 |
| 6.2.1.5_b | 0.5679 | 50.3902 | 0.1029 | 0.0000 |
| 6.4.1.1 | 0.0000 | 0.0000 | 0.4761 | 0.0000 |
| aa01b | 0.0141 | 0.0000 | 0.0014 | 0.0000 |
| aa02 | 0.0069 | 0.0000 | 0.0007 | 0.0000 |
| aa03 | 0.0051 | 0.0000 | 0.0005 | 0.0000 |
| aa04 | 0.0021 | 0.0000 | 0.0002 | 0.0000 |
| aa05 | 0.0590 | 0.0000 | 0.0060 | 0.0000 |
| aa06 | 0.0454 | 0.0000 | 0.0046 | 0.0000 |
| aa07 | 0.0034 | 0.0000 | 0.0003 | 0.0000 |
| aa08 | 0.0167 | 0.0000 | 0.0017 | 0.0000 |
| aa10 | 0.0223 | 0.0000 | 0.0023 | 0.0000 |
| aa11 | 0.0697 | 0.0000 | 0.0071 | 0.0000 |
| aa12 | 0.0201 | 0.0000 | 0.0021 | 0.0000 |
| aa13 | 0.0127 | 0.0000 | 0.0013 | 0.0000 |
| aa14 | 0.0107 | 0.0000 | 0.0011 | 0.0000 |
| aa15 | 0.0410 | 0.0000 | 0.0042 | 0.0000 |
| aa16 | 0.0123 | 0.0000 | 0.0013 | 0.0000 |
| aa17 | 0.0097 | 0.0000 | 0.0010 | 0.0000 |
| aa18 | 0.0082 | 0.0000 | 0.0008 | 0.0000 |
| aa20 | 0.0035 | 0.0000 | 0.0004 | 0.0000 |
| aa21 | 0.0089 | 0.0000 | 0.0009 | 0.0000 |
| aa22 | 0.0109 | 0.0000 | 0.0011 | 0.0000 |
| aa24 | 0.0057 | 0.0000 | 0.0006 | 0.0000 |
| aa25 | 0.0002 | 0.0000 | 0.0000 | 0.0000 |
| aa26 | 0.0157 | 0.0000 | 0.0016 | 0.0000 |
| Asp_res_ex | 0.0123 | 0.0000 | 0.0013 | 0.0000 |
| BIOM_ex | 0.0389 | 0.0000 | 0.0040 | 0.0000 |
| BIOM_IN | 0.0389 | 0.0000 | 0.0040 | 0.0000 |
| BM_2_Asp | 0.0123 | 0.0000 | 0.0013 | 0.0000 |
| BM_2_Ser | 0.0001 | 0.0000 | 0.0000 | 0.0000 |
| BM_2_Ser_res | 0.0001 | 0.0000 | 0.0000 | 0.0000 |
| BM_Acetyl_CoA | 0.0091 | 0.0000 | 0.0009 | 0.0000 |
| BM_Ala | 0.0223 | 0.0000 | 0.0023 | 0.0000 |
| BM_Arg | 0.0109 | 0.0000 | 0.0011 | 0.0000 |
| BM_Asn | 0.0089 | 0.0000 | 0.0009 | 0.0000 |
| BM_Asp | 0.0100 | 0.0000 | 0.0010 | 0.0000 |
| BM_C_THF | 0.0360 | 0.0000 | 0.0037 | 0.0000 |
| BM_CH3 | 0.0002 | 0.0000 | 0.0000 | 0.0000 |
| BM_CO2 | 0.0288 | 0.0000 | 0.0029 | 0.0000 |
| BM_Cys | 0.0034 | 0.0000 | 0.0003 | 0.0000 |
|  | **Net** | **Xch** | **Net STD** | **Xch STD** |
|  | [mmol g^-1^_DW_ h^-1^] | [mmol g^-1^_DW_ h^-1^] | [mmol g^-1^_DW_ h^-1^] | [mmol g^-1^_DW_ h^-1^] |
| BM_F6P | 0.0022 | 0.0000 | 0.0002 | 0.0000 |
| BM_G6P | 0.0001 | 0.0000 | 0.0000 | 0.0000 |
| BM_Gln | 0.0097 | 0.0000 | 0.0010 | 0.0000 |
| BM_Glu | 0.0108 | 0.0000 | 0.0011 | 0.0000 |
| BM_Gly | 0.0392 | 0.0000 | 0.0040 | 0.0000 |
| BM_Gol3P | 0.0006 | 0.0000 | 0.0001 | 0.0000 |
| BM_His | 0.0035 | 0.0000 | 0.0004 | 0.0000 |
| BM_Ile | 0.0107 | 0.0000 | 0.0011 | 0.0000 |
| BM_Leu | 0.0167 | 0.0000 | 0.0017 | 0.0000 |
| BM_Lys | 0.0127 | 0.0000 | 0.0013 | 0.0000 |
| BM_Met | 0.0057 | 0.0000 | 0.0006 | 0.0000 |
| BM_PEP | 0.0011 | 0.0000 | 0.0001 | 0.0000 |
| BM_Phe | 0.0069 | 0.0000 | 0.0007 | 0.0000 |
| BM_Pro | 0.0082 | 0.0000 | 0.0008 | 0.0000 |
| BM_PRPP | 0.0288 | 0.0000 | 0.0029 | 0.0000 |
| BM_Put_1 | 0.0014 | 0.0000 | 0.0001 | 0.0000 |
| BM_Put_2 | 0.0000 | 0.0000 | 0.0000 | 0.0000 |
| BM_Put_out | 0.0014 | 0.0000 | 0.0001 | 0.0000 |
| BM_Pyr | 0.0011 | 0.0000 | 0.0001 | 0.0000 |
| BM_Ser | 0.0080 | 0.0000 | 0.0008 | 0.0000 |
| BM_Suc_CoA | 0.0000 | 0.0000 | 0.0000 | 0.0000 |
| BM_Thr | 0.0094 | 0.0000 | 0.0010 | 0.0000 |
| BM_Trp | 0.0021 | 0.0000 | 0.0002 | 0.0000 |
| BM_Tyr | 0.0051 | 0.0000 | 0.0005 | 0.0000 |
| BM_Val | 0.0157 | 0.0000 | 0.0016 | 0.0000 |
| CO2_EX | 6.0825 | 0.0000 | 0.3397 | 0.0000 |
| FRUC_IN | 1.2313 | 0.0000 | 0.0528 | 0.0000 |
| Gly_ex | 0.0061 | 0.0000 | 0.0006 | 0.0000 |
| ODC | 1.1357 | 0.0000 | 0.2058 | 0.0000 |
| PDC | 1.8695 | 0.0000 | 0.1556 | 0.0000 |
| Ru5P_ex_help | 0.0000 | 0.0000 | 0.0000 | 0.0000 |
| Ru5P_X5P_ex_help | 0.0000 | 0.0000 | 0.0000 | 0.0000 |
| X5P_ex_help | 0.0000 | 0.0000 | 0.0000 | 0.0000 |
|  |  |  |  |  |

**Supplementary Figure S.2.3. (a)** Relative net fluxes for the *mucA-* Δ*algC* strain to the wild-type strains on a logarithmic scale. **(b)** Relative net fluxes for the *mucA-* Δ*algC* strain to the wild-type strain scaled by fructose uptake on a logarithmic scale. Colors indicating pathways are as follows: purple – anaplerotic section (ANA); red – Entner-Doudoroff pathway (EDP); green – Embden-Meyerhof-Parnas pathway (EMP); grey – pentose phosphate pathway (PPP); blue – tricarboxylic acid cycle and glyoxylate shunt (TCA); orange – fructose uptake (carbon uptake). The reactions aldolase (*FBP* ↔ *DHAP* + *GAP*) and triose phosphatase (*DHAP* ↔ *GAP*) are not included because the corresponding net fluxes proceed in opposite direction. For abbreviations of metabolites see Supplementary Table S.1.5.


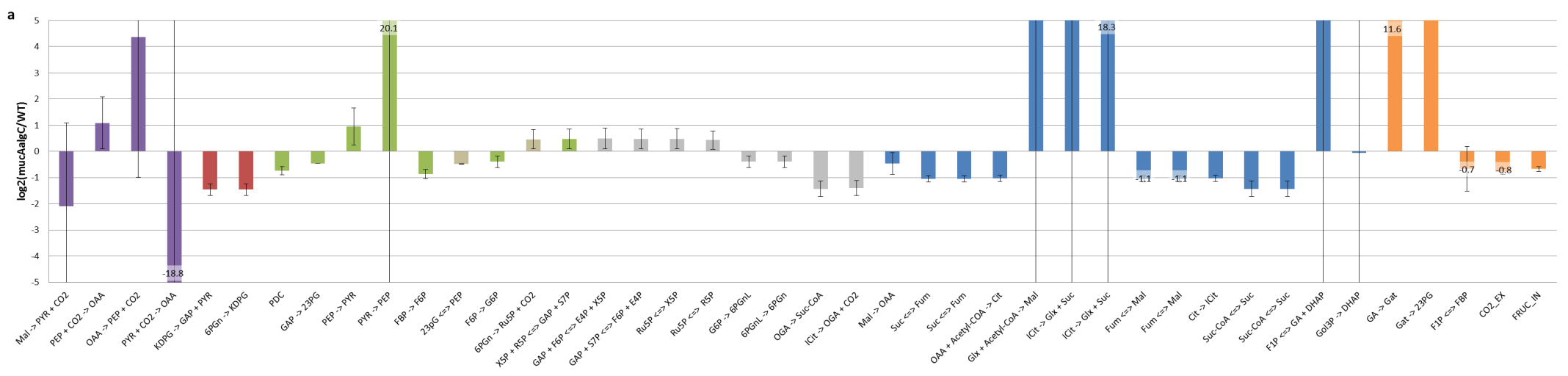


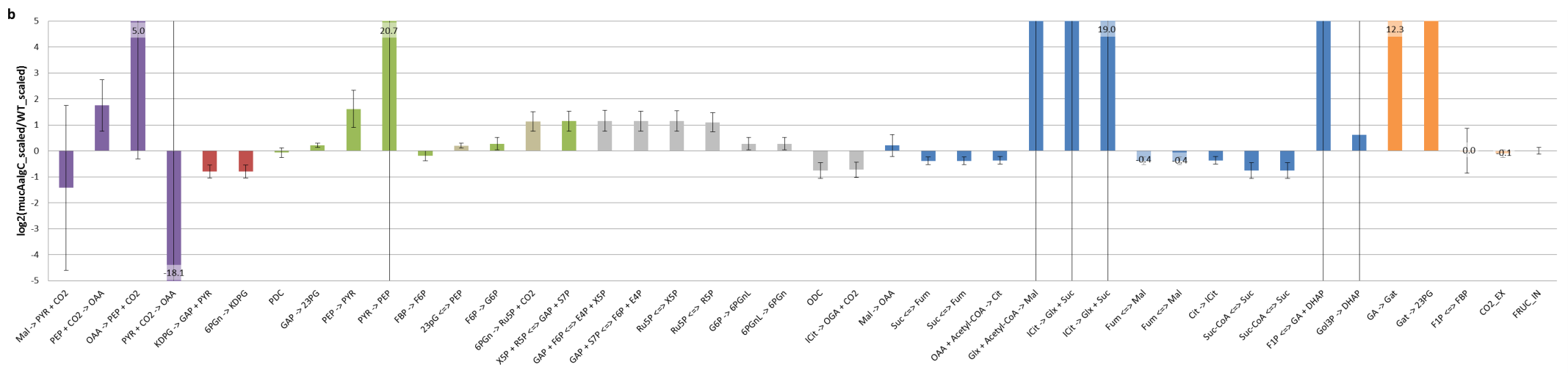


Supplementary Figure S.2.4. Reactions involving *ATP* and producing reducing power (NADH/NADPH) by central carbon metabolism of *P. fluorescens* SBW25 wild type (blue) and the *mucA-* Δ*algC* strain (red). (a) Individual reactions involving *ATP*. (b) Individual reactions producing *NADH*. (c) Individual reactions producing *NADPH*. List of metabolite abbreviations given in Supplementary Table S.1.5.
