# Supplementary material for: Fluxome study of Pseudomonas fluorescens reveals major reorganisation of carbon flux through central metabolic pathways in response to inactivation of the anti-sigma factor MucA
Source: BMC Syst Biol. 2015 Feb 18;9:6. doi: 10.1186/s12918-015-0148-0 (PMC4351692; doi:10.1186/s12918-015-0148-0)

**Supplementary Figure S.6.** The Figure shows the time profile of the ^13^C-labelled fructose *P. fluorescens algC*-Δ*mucA* double knock out cultivation. Color coding: weight reactor (turquoise), pH*10 (blue), dissolved oxygen (red), stirrer rate (green), CO_2_ off gas*50 (light blue), weight medium reservoir (violet), gas flow rate*100 (orange). As can be seen from both the dissolved oxygen and CO_2_ off gas concentration there is an initial batch growth phase followed by a switch to continuously mode by starting the pumping of fresh medium in and culture liquid out. The medium reservoir and the reactor are kept on weights and the In and Out pumps are weight-controlled. Samples were withdrawn at the end of the cultivation. The reactor is shown on next page.


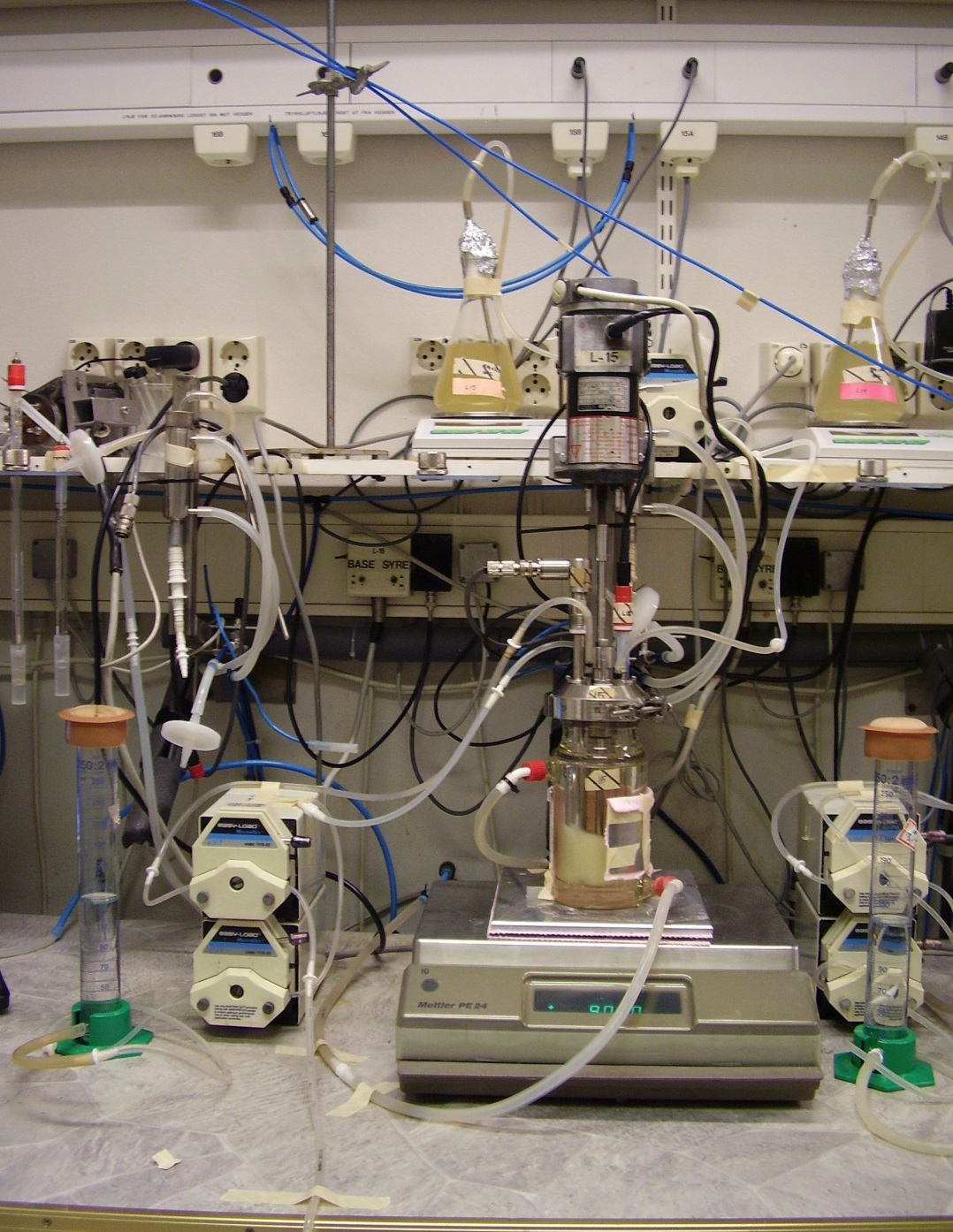

Supplement: Additional file 3: — Figure S.3 shows the time profile of the 13 C-labelled fructose P. fluorescens algC -ΔmucA double knock out cultivation. [file 12918_2015_148_MOESM3_ESM.docx]
