# Supplementary material for: Fluxome study of Pseudomonas fluorescens reveals major reorganisation of carbon flux through central metabolic pathways in response to inactivation of the anti-sigma factor MucA
Source: BMC Syst Biol. 2015 Feb 18;9:6. doi: 10.1186/s12918-015-0148-0 (PMC4351692; doi:10.1186/s12918-015-0148-0)
Supplement: Additional file 4: — Mass spectrometry settings for the LC-MS/MS (Table S.4.1) and GC-MS/MS (Table S.4.2) MRM acquisition methods and the model format of the measurements (last column). [file 12918_2015_148_MOESM4_ESM.docx]

**Supplementary Table S.3.1.** Mass spectrometry settings for the LC-MS/MS dynamic MRM acquisition method and the model format of the measurements (last column). For details about measurement specification and 13CFLUX2 model-files see Weitzel et al. 2012 (Weitzel M, Nöh K, Dalman T, Niedenfuhr S, Stute B, Wiechert W: 13CFLUX2 - high-performance software suite for 13C-metabolic flux analysis. Bioinformatics (Oxford, England) 2013, 29(1):143-145). The measurements are formulated in terms of MS-measurements because MRM-transitions have no product ions containing carbon atoms. HXP (hexose-x-phosphates): F1P and the sum G6P+M6P+F6P+G1P; P5P (pentose 5-phosphates): R5P and the sum RU5P+X5P=RX5P; TXP (triose x-phosphates): DHAP and GAP; n.d.: not detected in metabolite extracts. Remaining abbreviations are found in Supplementary Table S.1.5.

| **Measurement** | **MRM-transition (unit resolution)** | | | | **Fragmentor [V]** | **Collision energy [V]** | **RT [min]** | **Delta RT [min]** | **Model format** | |  |
| --- | --- | --- | --- | --- | --- | --- | --- | --- | --- | --- | --- |
|  | **Main precursor ion**  **(m/z)** | | **Main product ion (m/z)** | |  |  |  |  |  |  |  |
| 6PGN (m+0:m+0) | C6H12O10P- | 275,0 | PO3- | 79,1 | 100 | 45 | 60,9 | 20 | 6PGN  #M0,1,2,3,4,5,6 | |  |
| 6PGN (m+1:m+0) |  | 276,0 |  | 79,1 | 100 | 45 | 60,9 | 20 |  |  |  |
| 6PGN (m+2:m+0) |  | 277,0 |  | 79,1 | 100 | 45 | 60,9 | 20 |  |  |  |
| 6PGN (m+3:m+0) |  | 278,0 |  | 79,1 | 100 | 45 | 60,9 | 20 |  |  |  |
| 6PGN (m+4:m+0) |  | 279,0 |  | 79,1 | 100 | 45 | 60,9 | 20 |  |  |  |
| 6PGN (m+5:m+0) |  | 280,0 |  | 79,1 | 100 | 45 | 60,9 | 20 |  |  |  |
| 6PGN (m+6:m+0) |  | 281,0 |  | 79,1 | 100 | 45 | 60,9 | 20 |  |  |  |
| HXP (m+0:m+0) | C6H12O9P- | 259,0 | H2PO4- | 97,0 | 100 | 13 | 30,5 | 30 | F1P#M0,1,2,3,4,5,6  F6P#M0,1,2,3,4,5,6 | |  |
| HXP (m+1:m+0) |  | 260,0 |  | 97,0 | 100 | 13 | 30,5 | 30 |  |  |  |
| HXP (m+2:m+0) |  | 261,0 |  | 97,0 | 100 | 13 | 30,5 | 30 |  |  |  |
| HXP (m+3:m+0) |  | 262,0 |  | 97,0 | 100 | 13 | 30,5 | 30 |  |  |  |
| HXP (m+4:m+0) |  | 263,0 |  | 97,0 | 100 | 13 | 30,5 | 30 |  |  |  |
| HXP (m+5:m+0) |  | 264,0 |  | 97,0 | 100 | 13 | 30,5 | 30 |  |  |  |
| HXP (m+6:m+0) |  | 265,0 |  | 97,0 | 100 | 13 | 30,5 | 30 |  |  |  |
| P5P (m+0:m+0) | C5H10O8P- | 228,9 | H2PO4- | 97,0 | 80 | 5 | 30,0 | 30 | R5P#M0,1,2,3,4,5 | |  |
| P5P (m+1:m+0) |  | 229,9 |  | 97,0 | 80 | 5 | 30,0 | 30 |  |  |  |
| P5P (m+2:m+0) |  | 230,9 |  | 97,0 | 80 | 5 | 30,0 | 30 |  |  |  |
| P5P (m+3:m+0) |  | 231,9 |  | 97,0 | 80 | 5 | 30,0 | 30 |  |  |  |
| P5P (m+4:m+0) |  | 232,9 |  | 97,0 | 80 | 5 | 30,0 | 30 |  |  |  |
| P5P (m+5:m+0) |  | 233,9 |  | 97,0 | 80 | 5 | 30,0 | 30 |  |  |  |
| PEP (m+0:m+0) | C3H4O6P- | 166,8 | PO3- | 79,0 | 60 | 7 | 65,8 | 20 | PEP#M0,1,2,3 | |  |
| PEP (m+1:m+0) |  | 167,8 |  | 79,0 | 60 | 7 | 65,8 | 20 |  |  |  |
| PEP (m+2:m+0) |  | 168,8 |  | 79,0 | 60 | 7 | 65,8 | 20 |  |  |  |
| PEP (m+3:m+0) |  | 169,8 |  | 79,0 | 60 | 7 | 65,8 | 20 |  |  |  |
| PRPP (m+0:m+0) | C5H12O14P3- | 389,0 | P2O7H3- | 176,9 | 70 | 16 | 71,4 | 30 | PRPP#M0,1,2,3,4,5 | RX5P#M0,1,2,3,4,5 |  |
| PRPP (m+1:m+0) |  | 390,0 |  | 176,9 | 70 | 16 | 71,4 | 30 |  |  |  |
| PRPP (m+2:m+0) |  | 391,0 |  | 176,9 | 70 | 16 | 71,4 | 30 |  |  |  |
| PRPP (m+3:m+0) |  | 392,0 |  | 176,9 | 70 | 16 | 71,4 | 30 |  |  |  |
| PRPP (m+4:m+0) |  | 393,0 |  | 176,9 | 70 | 16 | 71,4 | 30 |  |  |  |
| PRPP (m+5:m+0) |  | 394,0 |  | 176,9 | 70 | 16 | 71,4 | 30 |  |  |  |
| TXP (m+0:m+0) | C3H6O6P- | 169,0 | H2PO4- | 97,0 | 60 | 3 | 33,0 | 30 | DHAP#M0,1,2,3 | |  |
| TXP (m+1:m+0) |  | 170,0 |  | 97,0 | 60 | 3 | 33,0 | 30 |  |  |  |
| TXP (m+2:m+0) |  | 171,0 |  | 97,0 | 60 | 3 | 33,0 | 30 |  |  |  |
| TXP (m+3:m+0) |  | 172,0 |  | 97,0 | 60 | 3 | 33,0 | 30 |  |  |  |

**Supplementary Table S.3.2.** Mass spectrometry settings for the GC-MS/MS MRM acquisition method and the model format of the measurements (last column). For details about measurement specification and 13CFLUX2 model-files see Weitzel et al. 2012. For short, carbons of the precursor ion and the product ion originating from the metabolite are given (numbering according to InChI code). When determining the identity of precursor ions and product ions for amino acid it was assumed for all metabolites except for *PHE* that fragmentation proceeds from the ester group rather than from the amide group of the derivate due to production of stable conjugated double bonds. Measurements for which the product ion could not be identified were formulated as MS-measurements instead of MS/MS-measurements. The information is not given for metabolites not detected in the extracts (*ASN*, *CYS*, *HIS*, *ICIT*, *LEU*, *MAL*, *OAA*, *ORN*, *SER* and *TRP*). For abbreviations see Supplementary Table S.1.5.

| **Measurement** | **MRM-transition (unit resolution)** | | | | | **Time segment** | **RT [min]** | **Dwell time [ms]** | **Collison energy [V]** | **Model format** | |
| --- | --- | --- | --- | --- | --- | --- | --- | --- | --- | --- | --- |
|  | **Main precursor ion**  **(m/z)** | | **Main product ion**  **(m/z)** | | |  |  |  |  |  |  |
| ALA (m+0:m+0) | C4H8N1O2+ | 101,8 | |  | 58,0 | 2 | 10,0 | 2 | 5 | ALA[1,2] #M0,1,2 | |
| ALA (m+1:m+0) |  | 102,8 | |  | 58,0 | 2 | 10,0 | 2 | 5 |  |  |
| ALA (m+1:m+1) |  | 102,8 | |  | 59,0 | 2 | 10,0 | 2 | 5 |  |  |
| ALA (m+2:m+0) |  | 103,8 | |  | 58,0 | 2 | 10,0 | 2 | 5 |  |  |
| ALA (m+2:m+1) |  | 103,8 | |  | 59,0 | 2 | 10,0 | 2 | 5 |  |  |
| ALA (m+2:m+2) |  | 103,8 | |  | 60,0 | 2 | 10,0 | 2 | 5 |  |  |
| ALA (m+3:m+1) |  | 104,8 | |  | 59,0 | 2 | 10,0 | 2 | 5 |  |  |
| ALA (m+3:m+2) |  | 104,8 | |  | 60,0 | 2 | 10,0 | 2 | 5 |  |  |
| ALA (m+4:m+2) |  | 105,8 | |  | 60,0 | 2 | 10,0 | 2 | 5 |  |  |
| ASP (m+0:m+0) | C6H10NO4+ | 159,8 | | C5H6NO3+ | 128,0 | 6 | 14,0 | 2 | 5 | ASP[1-3:1-3] | #M(0,0),(1,1),(2,2),(3,3) |
| ASP (m+1:m+0) |  | 160,8 | |  | 128,0 | 6 | 14,0 | 2 | 5 |  |  |
| ASP (m+1:m+1) |  | 160,8 | |  | 129,0 | 6 | 14,0 | 2 | 5 |  |  |
| ASP (m+2:m+1) |  | 161,8 | |  | 129,0 | 6 | 14,0 | 2 | 5 |  |  |
| ASP (m+2:m+2) |  | 161,8 | |  | 130,0 | 6 | 14,0 | 2 | 5 |  |  |
| ASP (m+3:m+2) |  | 162,8 | |  | 130,0 | 6 | 14,0 | 2 | 5 |  |  |
| ASP (m+3:m+3) |  | 162,8 | |  | 131,0 | 6 | 14,0 | 2 | 5 |  |  |
| ASP (m+4:m+3) |  | 163,8 | |  | 131,0 | 6 | 14,0 | 2 | 5 |  |  |
| ASP (m+4:m+4) |  | 163,8 | |  | 132,0 | 6 | 14,0 | 2 | 5 |  |  |
| ASP (m+5:m+4) |  | 164,8 | |  | 132,0 | 6 | 14,0 | 2 | 5 |  |  |
| ASP (m+5:m+5) |  | 164,8 | |  | 133,0 | 6 | 14,0 | 2 | 5 |  |  |
| ASP (m+6:m+5) |  | 165,8 | |  | 133,0 | 6 | 14,0 | 2 | 5 |  |  |
| GLU (m+0:m+0) | C9H16NO6+ | 233,8 | | C7H12NO4+ | 174,2 | 8 | 16,6 | 2 | 5 | GLU[1-5:1-4] | #M(0,0),(1,0),(1,1),(2,1),(2,2),(3,2),(3,3),(4,3),(4,4),(5,4) |
| GLU (m+1:m+0) |  | 234,8 | |  | 174,2 | 8 | 16,6 | 2 | 5 |  |  |
| GLU (m+1:m+1) |  | 234,8 | |  | 175,2 | 8 | 16,6 | 2 | 5 |  |  |
| GLU (m+2:m+0) |  | 235,8 | |  | 174,2 | 8 | 16,6 | 2 | 5 |  |  |
| GLU (m+2:m+1) |  | 235,8 | |  | 175,2 | 8 | 16,6 | 2 | 5 |  |  |
| GLU (m+2:m+2) |  | 235,8 | |  | 176,2 | 8 | 16,6 | 2 | 5 |  |  |
| GLU (m+3:m+1) |  | 236,8 | |  | 175,2 | 8 | 16,6 | 2 | 5 |  |  |
| GLU (m+3:m+2) |  | 236,8 | |  | 176,2 | 8 | 16,6 | 2 | 5 |  |  |
| GLU (m+3:m+3) |  | 236,8 | |  | 177,2 | 8 | 16,6 | 2 | 5 |  |  |
| GLU (m+4:m+2) |  | 237,8 | |  | 176,2 | 8 | 16,6 | 2 | 5 |  |  |
| GLU (m+4:m+3) |  | 237,8 | |  | 177,2 | 8 | 16,6 | 2 | 5 |  |  |
| GLU (m+4:m+4) |  | 237,8 | |  | 178,2 | 8 | 16,6 | 2 | 5 |  |  |
| GLU (m+5:m+3) |  | 238,8 | |  | 177,2 | 8 | 16,6 | 2 | 5 |  |  |
| GLU (m+5:m+4) |  | 238,8 | |  | 178,2 | 8 | 16,6 | 2 | 5 |  |  |
| GLU (m+5:m+5) |  | 238,8 | |  | 179,2 | 8 | 16,6 | 2 | 5 |  |  |
| GLU (m+6:m+4) |  | 239,8 | |  | 178,2 | 8 | 16,6 | 2 | 5 |  |  |
| GLU (m+6:m+5) |  | 239,8 | |  | 179,2 | 8 | 16,6 | 2 | 5 |  |  |
| GLU (m+6:m+6) |  | 239,8 | |  | 180,2 | 8 | 16,6 | 2 | 5 |  |  |
| GLU (m+7:m+5) |  | 240,8 | |  | 179,2 | 8 | 16,6 | 2 | 5 |  |  |
| GLU (m+7:m+6) |  | 240,8 | |  | 180,2 | 8 | 16,6 | 2 | 5 |  |  |
| GLU (m+7:m+7) |  | 240,8 | |  | 181,2 | 8 | 16,6 | 2 | 5 |  |  |
| GLU (m+8:m+6) |  | 241,8 | |  | 180,2 | 8 | 16,6 | 2 | 5 |  |  |
| GLU (m+8:m+7) |  | 241,8 | |  | 181,2 | 8 | 16,6 | 2 | 5 |  |  |
| GLU (m+9:m+7) |  | 242,8 | |  | 181,2 | 8 | 16,6 | 2 | 5 |  |  |
| GLX (m+0:m+0) | C5H9O3+ | 116,8 | |  | 45,0 | 1 | 8,1 | 2 | 5 | GLX  #M0,1,2 | |
| GLX (m+1:m+0) |  | 117,8 | |  | 45,0 | 1 | 8,1 | 2 | 5 |  |  |
| GLX (m+1:m+1) |  | 117,8 | |  | 46,0 | 1 | 8,1 | 2 | 5 |  |  |
| GLX (m+2:m+0) |  | 118,8 | |  | 45,0 | 1 | 8,1 | 2 | 5 |  | |
| GLX (m+2:m+1) |  | 118,8 | |  | 46,0 | 1 | 8,1 | 2 | 5 |  |  |
| GLX (m+2:m+2) |  | 118,8 | |  | 47,0 | 1 | 8,1 | 2 | 5 |  |  |
| GLX (m+3:m+0) |  | 119,8 | |  | 45,0 | 1 | 8,1 | 2 | 5 |  |  |
| GLX (m+3:m+1) |  | 119,8 | |  | 46,0 | 1 | 8,1 | 2 | 5 |  |  |
| GLX (m+3:m+2) |  | 119,8 | |  | 47,0 | 1 | 8,1 | 2 | 5 |  |  |
| GLX (m+4:m+1) |  | 120,8 | |  | 46,0 | 1 | 8,1 | 2 | 5 |  |  |
| GLX (m+4:m+2) |  | 120,8 | |  | 47,0 | 1 | 8,1 | 2 | 5 |  |  |
| GLX (m+5:m+2) |  | 121,8 | |  | 47,0 | 1 | 8,1 | 2 | 5 |  |  |
| GLY (m+0:m+0) | C3H6NO2+ | 87,8 | | C2H6N+ | 44,0 | 2 | 8,1 | 2 | 5 | GLY[1:1] | #M(0,0),(1,1) |
| GLY (m+1:m+0) |  | 88,8 | |  | 44,0 | 2 | 8,1 | 2 | 5 |  |  |
| GLY (m+1:m+1) |  | 88,8 | |  | 45,0 | 2 | 8,1 | 2 | 5 |  |  |
| GLY (m+2:m+1) |  | 89,8 | |  | 45,0 | 2 | 8,1 | 2 | 5 |  |  |
| GLY (m+2:m+2) |  | 89,8 | |  | 46,0 | 2 | 8,1 | 2 | 5 |  |  |
| GLY (m+3:m+2) |  | 90,8 | |  | 46,0 | 2 | 8,1 | 2 | 5 |  |  |
| ILE (m+0:m+0) | C9H18NO4+ | 203,9 | | C7H14NO2+ | 144,1 | 4 | 13,3 | 2 | 5 | ILE[1-6:1-5] | #M(0,0),(1,0),(1,1),(2,1),(2,2),(3,2),(3,3),(4,3),(4,4),(5,4),(5,5),(6,5) |
| ILE (m+1:m+0) |  | 204,9 | |  | 144,1 | 4 | 13,3 | 2 | 5 |  |  |
| ILE (m+1:m+1) |  | 204,9 | |  | 145,1 | 4 | 13,3 | 2 | 5 |  |  |
| ILE (m+2:m+0) |  | 205,9 | |  | 144,1 | 4 | 13,3 | 2 | 5 |  |  |
| ILE (m+2:m+1) |  | 205,9 | |  | 145,1 | 4 | 13,3 | 2 | 5 |  |  |
| ILE (m+2:m+2) |  | 205,9 | |  | 146,1 | 4 | 13,3 | 2 | 5 |  |  |
| ILE (m+3:m+1) |  | 206,9 | |  | 145,1 | 4 | 13,3 | 2 | 5 |  |  |
| ILE (m+3:m+2) |  | 206,9 | |  | 146,1 | 4 | 13,3 | 2 | 5 |  |  |
| ILE (m+3:m+3) |  | 206,9 | |  | 147,1 | 4 | 13,3 | 2 | 5 |  |  |
| ILE (m+4:m+2) |  | 207,9 | |  | 146,1 | 4 | 13,3 | 2 | 5 |  |  |
| ILE (m+4:m+3) |  | 207,9 | |  | 147,1 | 4 | 13,3 | 2 | 5 |  |  |
| ILE (m+4:m+4) |  | 207,9 | |  | 148,1 | 4 | 13,3 | 2 | 5 |  |  |
| ILE (m+5:m+3) |  | 208,9 | |  | 147,1 | 4 | 13,3 | 2 | 5 |  |  |
| ILE (m+5:m+4) |  | 208,9 | |  | 148,1 | 4 | 13,3 | 2 | 5 |  |  |
| ILE (m+5:m+5) |  | 208,9 | |  | 149,1 | 4 | 13,3 | 2 | 5 |  |  |
| ILE (m+6:m+4) |  | 209,9 | |  | 148,1 | 4 | 13,3 | 2 | 5 |  |  |
| ILE (m+6:m+5) |  | 209,9 | |  | 149,1 | 4 | 13,3 | 2 | 5 |  |  |
| ILE (m+6:m+6) |  | 209,9 | |  | 150,1 | 4 | 13,3 | 2 | 5 |  |  |
| ILE (m+7:m+5) |  | 210,9 | |  | 149,1 | 4 | 13,3 | 2 | 5 |  |  |
| ILE (m+7:m+6) |  | 210,9 | |  | 150,1 | 4 | 13,3 | 2 | 5 |  |  |
| ILE (m+7:m+7) |  | 210,9 | |  | 151,1 | 4 | 13,3 | 2 | 5 |  |  |
| ILE (m+8:m+6) |  | 211,9 | |  | 150,1 | 4 | 13,3 | 2 | 5 |  |  |
| ILE (m+8:m+7) |  | 211,9 | |  | 151,1 | 4 | 13,3 | 2 | 5 |  |  |
| ILE (m+9:m+7) |  | 212,9 | |  | 151,1 | 4 | 13,3 | 2 | 5 |  |  |
| LYS (m+0:m+0) | C10H17O5N2+ | 244,9 | |  | 142,1 | 11 | 21,5 | 2 | 5 | LYS#M0,1,2,3,4,5,6 | |
| LYS (m+1:m+0) |  | 245,9 | |  | 142,1 | 11 | 21,5 | 2 | 5 |  |  |
| LYS (m+1:m+1) |  | 245,9 | |  | 143,1 | 11 | 21,5 | 2 | 5 |  |  |
| LYS (m+2:m+0) |  | 246,9 | |  | 142,1 | 11 | 21,5 | 2 | 5 |  |  |
| LYS (m+2:m+1) |  | 246,9 | |  | 143,1 | 11 | 21,5 | 2 | 5 |  |  |
| LYS (m+2:m+2) |  | 246,9 | |  | 144,1 | 11 | 21,5 | 2 | 5 |  |  |
| LYS (m+3:m+0) |  | 247,9 | |  | 142,1 | 11 | 21,5 | 2 | 5 |  |  |
| LYS (m+3:m+1) |  | 247,9 | |  | 143,1 | 11 | 21,5 | 2 | 5 |  |  |
| LYS (m+3:m+2) |  | 247,9 | |  | 144,1 | 11 | 21,5 | 2 | 5 |  |  |
| LYS (m+3:m+3) |  | 247,9 | |  | 145,1 | 11 | 21,5 | 2 | 5 |  |  |
| LYS (m+4:m+1) |  | 248,9 | |  | 143,1 | 11 | 21,5 | 2 | 5 |  |  |
| LYS (m+4:m+2) |  | 248,9 | |  | 144,1 | 11 | 21,5 | 2 | 5 |  |  |
| LYS (m+4:m+3) |  | 248,9 | |  | 145,1 | 11 | 21,5 | 2 | 5 |  |  |
| LYS (m+4:m+4) |  | 248,9 | |  | 146,1 | 11 | 21,5 | 2 | 5 |  |  |
| LYS (m+5:m+2) |  | 249,9 | |  | 144,1 | 11 | 21,5 | 2 | 5 |  |  |
| LYS (m+5:m+3) |  | 249,9 | |  | 145,1 | 11 | 21,5 | 2 | 5 |  |  |
| LYS (m+5:m+4) |  | 249,9 | |  | 146,1 | 11 | 21,5 | 2 | 5 |  |  |
| LYS (m+5:m+5) |  | 249,9 | |  | 147,1 | 11 | 21,5 | 2 | 5 |  |  |
| LYS (m+6:m+3) |  | 250,9 | |  | 145,1 | 11 | 21,5 | 2 | 5 |  |  |
| LYS (m+6:m+4) |  | 250,9 | |  | 146,1 | 11 | 21,5 | 2 | 5 |  |  |
| LYS (m+6:m+5) |  | 250,9 | |  | 147,1 | 11 | 21,5 | 2 | 5 |  |  |
| LYS (m+6:m+6) |  | 250,9 | |  | 148,1 | 11 | 21,5 | 2 | 5 |  |  |
| LYS (m+7:m+4) |  | 251,9 | |  | 146,1 | 11 | 21,5 | 2 | 5 |  |  |
| LYS (m+7:m+5) |  | 251,9 | |  | 147,1 | 11 | 21,5 | 2 | 5 |  |  |
| LYS (m+7:m+6) |  | 251,9 | |  | 148,1 | 11 | 21,5 | 2 | 5 |  |  |
| LYS (m+7:m+7) |  | 251,9 | |  | 149,1 | 11 | 21,5 | 2 | 5 |  |  |
| LYS (m+8:m+5) |  | 252,9 | |  | 147,1 | 11 | 21,5 | 2 | 5 |  |  |
| LYS (m+8:m+6) |  | 252,9 | |  | 148,1 | 11 | 21,5 | 2 | 5 |  |  |
| LYS (m+8:m+7) |  | 252,9 | |  | 149,1 | 11 | 21,5 | 2 | 5 |  |  |
| LYS (m+9:m+6) |  | 253,9 | |  | 148,1 | 11 | 21,5 | 2 | 5 |  |  |
| LYS (m+9:m+7) |  | 253,9 | |  | 149,1 | 11 | 21,5 | 2 | 5 |  |  |
| LYS (m+10:m+7) |  | 254,9 | |  | 149,1 | 11 | 21,5 | 2 | 5 |  |  |
| MET (m+0:m+0) | C6H12NO2S+ | 161,8 | |  | 114,0 | 8 | 16,8 | 2 | 5 | MET[1-4:2-4] #M(0,0),(1,0), | (1,1),(2,1),(2,2),(3,2),(3,3),(4,3) |
| MET (m+1:m+0) |  | 162,8 | |  | 114,0 | 8 | 16,8 | 2 | 5 |  |  |
| MET (m+1:m+1) |  | 162,8 | |  | 115,0 | 8 | 16,8 | 2 | 5 |  |  |
| MET (m+2:m+1) |  | 163,8 | |  | 115,0 | 8 | 16,8 | 2 | 5 |  |  |
| MET (m+2:m+2) |  | 163,8 | |  | 116,0 | 8 | 16,8 | 2 | 5 |  |  |
| MET (m+3:m+2) |  | 164,8 | |  | 116,0 | 8 | 16,8 | 2 | 5 |  |  |
| MET (m+3:m+3) |  | 164,8 | |  | 117,0 | 8 | 16,8 | 2 | 5 |  |  |
| MET (m+4:m+3) |  | 165,8 | |  | 117,0 | 8 | 16,8 | 2 | 5 |  |  |
| MET (m+4:m+4) |  | 165,8 | |  | 118,0 | 8 | 16,8 | 2 | 5 |  |  |
| MET (m+5:m+4) |  | 166,8 | |  | 118,0 | 8 | 16,8 | 2 | 5 |  |  |
| MET (m+5:m+5) |  | 166,8 | |  | 119,0 | 8 | 16,8 | 2 | 5 |  |  |
| MET (m+6:m+5) |  | 167,8 | |  | 119,0 | 8 | 16,8 | 2 | 5 |  |  |
| OGA (m+0:m+0) | C6H7O4+ | 143,8 | |  | 115,0 | 3 | 12,1 | 2 | 5 | OGA#M0,1,2,3,4,5 | |
| OGA (m+1:m+0) |  | 143,8 | |  | 115,0 | 3 | 12,1 | 2 | 5 |  |  |
| OGA (m+1:m+1) |  | 143,8 | |  | 116,0 | 3 | 12,1 | 2 | 5 |  |  |
| OGA (m+2:m+1) |  | 144,8 | |  | 116,0 | 3 | 12,1 | 2 | 5 |  |  |
| OGA (m+2:m+2) |  | 144,8 | |  | 117,0 | 3 | 12,1 | 2 | 5 |  |  |
| OGA (m+3:m+2) |  | 145,8 | |  | 117,0 | 3 | 12,1 | 2 | 5 |  |  |
| OGA (m+3:m+3) |  | 145,8 | |  | 118,0 | 3 | 12,1 | 2 | 5 |  |  |
| OGA (m+4:m+3) |  | 146,8 | |  | 118,0 | 3 | 12,1 | 2 | 5 |  |  |
| OGA (m+4:m+4) |  | 146,8 | |  | 119,0 | 3 | 12,1 | 2 | 5 |  |  |
| OGA (m+5:m+4) |  | 147,8 | |  | 119,0 | 3 | 12,1 | 2 | 5 |  |  |
| OGA (m+5:m+5) |  | 147,8 | |  | 120,0 | 3 | 12,1 | 2 | 5 |  |  |
| OGA (m+6:m+5) |  | 148,8 | |  | 120,0 | 3 | 12,1 | 2 | 5 |  |  |
| PYR (m+0:m+0) | C5H9O3+ | 116,8 | | C3H5O+ | 57,0 | 1 | 7,2 | 2 | 10 | PYR[1-3:1-2] | #M(0,0),(1,0),(1,1),(2,1),(2,2),(3,2) |
| PYR (m+1:m+0) |  | 117,8 | |  | 57,0 | 1 | 7,2 | 2 | 10 |  |  |
| PYR (m+1:m+1) |  | 117,8 | |  | 58,0 | 1 | 7,2 | 2 | 10 |  |  |
| PYR (m+2:m+0) |  | 118,8 | |  | 57,0 | 1 | 7,2 | 2 | 10 |  |  |
| PYR (m+2:m+1) |  | 118,8 | |  | 58,0 | 1 | 7,2 | 2 | 10 |  |  |
| PYR (m+2:m+2) |  | 118,8 | |  | 59,0 | 1 | 7,2 | 2 | 10 |  |  |
| PYR (m+3:m+1) |  | 119,8 | |  | 58,0 | 1 | 7,2 | 2 | 10 |  |  |
| PYR (m+3:m+2) |  | 119,8 | |  | 59,0 | 1 | 7,2 | 2 | 10 |  |  |
| PYR (m+3:m+3) |  | 119,8 | |  | 60,0 | 1 | 7,2 | 2 | 10 |  |  |
| PYR (m+4:m+2) |  | 120,8 | |  | 59,0 | 1 | 7,2 | 2 | 10 |  |  |
| PYR (m+4:m+3) |  | 120,8 | |  | 60,0 | 1 | 7,2 | 2 | 10 |  |  |
| PYR (m+5:m+3) |  | 121,8 | |  | 60,0 | 1 | 7,2 | 2 | 10 |  |  |
| PHE (m+0:m+0) | C10H12O2N+ | 177,9 | | C9H8ON+ | 146,1 | 9 | 18,2 | 2 | 10 |  |  |
| PHE (m+1:m+0) |  | 178,9 | |  | 146,1 | 9 | 18,2 | 2 | 10 |  |  |
| PHE (m+1:m+1) |  | 178,9 | |  | 147,1 | 9 | 18,2 | 2 | 10 |  |  |
| PHE (m+2:m+1) |  | 179,9 | |  | 147,1 | 9 | 18,2 | 2 | 10 |  |  |
| PHE (m+2:m+2) |  | 179,9 | |  | 148,1 | 9 | 18,2 | 2 | 10 |  |  |
| PHE (m+3:m+2) |  | 180,9 | |  | 148,1 | 9 | 18,2 | 2 | 10 |  |  |
| PHE (m+3:m+3) |  | 180,9 | |  | 149,1 | 9 | 18,2 | 2 | 10 |  |  |
| PHE (m+4:m+3) |  | 181,9 | |  | 149,1 | 9 | 18,2 | 2 | 10 |  |  |
| PHE (m+4:m+4) |  | 181,9 | |  | 150,1 | 9 | 18,2 | 2 | 10 |  |  |
| PHE (m+3:m+2) |  | 180,9 | |  | 148,1 | 9 | 18,2 | 2 | 10 | PHE[1-9:1-9] #M(0,0),(1,1),(2,2), | (3,3),(4,4),(5,5),(6,6),(7,7),(8,8),(9,9) |
| PHE (m+3:m+3) |  | 180,9 | |  | 149,1 | 9 | 18,2 | 2 | 10 |  |  |
| PHE (m+4:m+3) |  | 181,9 | |  | 149,1 | 9 | 18,2 | 2 | 10 |  |  |
| PHE (m+4:m+4) |  | 181,9 | |  | 150,1 | 9 | 18,2 | 2 | 10 |  |  |
| PHE (m+5:m+4) |  | 182,9 | |  | 150,1 | 9 | 18,2 | 2 | 10 |  |  |
| PHE (m+5:m+5) |  | 182,9 | |  | 151,1 | 9 | 18,2 | 2 | 10 |  |  |
| PHE (m+6:m+5) |  | 183,9 | |  | 151,1 | 9 | 18,2 | 2 | 10 |  |  |
| PHE (m+6:m+6) |  | 183,9 | |  | 152,1 | 9 | 18,2 | 2 | 10 |  |  |
| PHE (m+7:m+6) |  | 184,9 | |  | 152,1 | 9 | 18,2 | 2 | 10 |  |  |
| PHE (m+7:m+7) |  | 184,9 | |  | 153,1 | 9 | 18,2 | 2 | 10 |  |  |
| PHE (m+8:m+7) |  | 185,9 | |  | 153,1 | 9 | 18,2 | 2 | 10 |  |  |
| PHE (m+8:m+8) |  | 185,9 | |  | 154,1 | 9 | 18,2 | 2 | 10 |  |  |
| PHE (m+9:m+8) |  | 186,9 | |  | 154,1 | 9 | 18,2 | 2 | 10 |  |  |
| PHE (m+9:m+9) |  | 186,9 | |  | 155,1 | 9 | 18,2 | 2 | 10 |  |  |
| PHE (m+10:m+9) |  | 187,9 | |  | 155,1 | 9 | 18,2 | 2 | 10 |  |  |
| PRO (m+0:m+0) | C8H14O4N+ | 187,9 | | C6H10O2N+ | 128,1 | 5 | 13,9 | 2 | 10 | PRO[1-5:1-4] | #M(0,0),(1,0),(1,1),(2,1),(2,2),(3,2),(3,3),(4,3),(4,4),(5,4) |
| PRO (m+1:m+0) |  | 188,9 | |  | 128,1 | 5 | 13,9 | 2 | 10 |  |  |
| PRO (m+1:m+1) |  | 188,9 | |  | 129,1 | 5 | 13,9 | 2 | 10 |  |  |
| PRO (m+2:m+0) |  | 189,9 | |  | 128,1 | 5 | 13,9 | 2 | 10 |  |  |
| PRO (m+2:m+1) |  | 189,9 | |  | 129,1 | 5 | 13,9 | 2 | 10 |  |  |
| PRO (m+2:m+2) |  | 189,9 | |  | 130,1 | 5 | 13,9 | 2 | 10 |  |  |
| PRO (m+3:m+1) |  | 190,9 | |  | 129,1 | 5 | 13,9 | 2 | 10 |  |  |
| PRO (m+3:m+2) |  | 190,9 | |  | 130,1 | 5 | 13,9 | 2 | 10 |  |  |
| PRO (m+3:m+3) |  | 190,9 | |  | 131,1 | 5 | 13,9 | 2 | 10 |  |  |
| PRO (m+4:m+2) |  | 191,9 | |  | 130,1 | 5 | 13,9 | 2 | 10 |  |  |
| PRO (m+4:m+3) |  | 191,9 | |  | 131,1 | 5 | 13,9 | 2 | 10 |  |  |
| PRO (m+4:m+4) |  | 191,9 | |  | 132,1 | 5 | 13,9 | 2 | 10 |  |  |
| PRO (m+5:m+3) |  | 192,9 | |  | 131,1 | 5 | 13,9 | 2 | 10 |  |  |
| PRO (m+5:m+4) |  | 192,9 | |  | 132,1 | 5 | 13,9 | 2 | 10 |  |  |
| PRO (m+5:m+5) |  | 192,9 | |  | 133,1 | 5 | 13,9 | 2 | 10 |  |  |
| PRO (m+6:m+4) |  | 193,9 | |  | 132,1 | 5 | 13,9 | 2 | 10 |  |  |
| PRO (m+6:m+5) |  | 193,9 | |  | 133,1 | 5 | 13,9 | 2 | 10 |  |  |
| PRO (m+6:m+6) |  | 193,9 | |  | 134,1 | 5 | 13,9 | 2 | 10 |  |  |
| PRO (m+7:m+5) |  | 194,9 | |  | 133,1 | 5 | 13,9 | 2 | 10 |  |  |
| PRO (m+7:m+6) |  | 194,9 | |  | 134,1 | 5 | 13,9 | 2 | 10 |  |  |
| PRO (m+8:m+6) |  | 195,9 | |  | 134,1 | 5 | 13,9 | 2 | 10 |  |  |
| TYR (m+0:m+0) | C12H14O5N+ | 251,8 | | C11H10O4N+ | 220,1 | 13 | 23,2 | 2 | 5 | TYR[1-9:1-9] | #M(0,0),(1,1),(2,2),(3,3),(4,4),(5,5),(6,6),(7,7),(8,8),(9,9) |
| TYR (m+1:m+0) |  | 252,8 | |  | 220,1 | 13 | 23,2 | 2 | 5 |  |  |
| TYR (m+1:m+1) |  | 252,8 | |  | 221,1 | 13 | 23,2 | 2 | 5 |  |  |
| TYR (m+2:m+1) |  | 253,8 | |  | 221,1 | 13 | 23,2 | 2 | 5 |  |  |
| TYR (m+2:m+2) |  | 253,8 | |  | 222,1 | 13 | 23,2 | 2 | 5 |  |  |
| TYR (m+3:m+2) |  | 254,8 | |  | 222,1 | 13 | 23,2 | 2 | 5 |  |  |
| TYR (m+3:m+3) |  | 254,8 | |  | 223,1 | 13 | 23,2 | 2 | 5 |  |  |
| TYR (m+4:m+3) |  | 255,8 | |  | 223,1 | 13 | 23,2 | 2 | 5 |  |  |
| TYR (m+4:m+4) |  | 255,8 | |  | 224,1 | 13 | 23,2 | 2 | 5 |  |  |
| TYR (m+5:m+4) |  | 256,8 | |  | 224,1 | 13 | 23,2 | 2 | 5 |  |  |
| TYR (m+5:m+5) |  | 256,8 | |  | 225,1 | 13 | 23,2 | 2 | 5 |  |  |
| TYR (m+6:m+5) |  | 257,8 | |  | 225,1 | 13 | 23,2 | 2 | 5 |  |  |
| TYR (m+6:m+6) |  | 257,8 | |  | 226,1 | 13 | 23,2 | 2 | 5 |  |  |
| TYR (m+7:m+6) |  | 258,8 | |  | 226,1 | 13 | 23,2 | 2 | 5 |  |  |
| TYR (m+7:m+7) |  | 258,8 | |  | 227,1 | 13 | 23,2 | 2 | 5 |  |  |
| TYR (m+8:m+7) |  | 259,8 | |  | 227,1 | 13 | 23,2 | 2 | 5 |  |  |
| TYR (m+8:m+8) |  | 259,8 | |  | 228,1 | 13 | 23,2 | 2 | 5 |  |  |
| TYR (m+9:m+8) |  | 260,8 | |  | 228,1 | 13 | 23,2 | 2 | 5 |  |  |
| TYR (m+9:m+9) |  | 260,8 | |  | 229,1 | 13 | 23,2 | 2 | 5 |  |  |
| TYR (m+10:m+9) |  | 261,8 | |  | 229,1 | 13 | 23,2 | 2 | 5 |  |  |
| TYR (m+10:m+10) |  | 261,8 | |  | 230,1 | 13 | 23,2 | 2 | 5 |  |  |
| TYR (m+11:m+10) |  | 262,8 | |  | 230,1 | 13 | 23,2 | 2 | 5 |  |  |
| TYR (m+11:m+11) |  | 262,8 | |  | 231,1 | 13 | 23,2 | 2 | 5 |  |  |
| TYR (m+12:m+11) |  | 263,8 | |  | 231,1 | 13 | 23,2 | 2 | 5 |  |  |
| VAL (m+0:m+0) | C6H12O2N+ | 130,1 | | C5H8ON+ | 98,0 | 3 | 12,0 | 2 | 5 | VAL[1-4:1-4] | #M(0,0),(1,1),(2,2),(3,3),(4,4) |
| VAL (m+1:m+0) |  | 131,1 | |  | 98,0 | 3 | 12,0 | 2 | 5 |  |  |
| VAL (m+1:m+1) |  | 131,1 | |  | 99,0 | 3 | 12,0 | 2 | 5 |  |  |
| VAL (m+2:m+1) |  | 132,1 | |  | 99,0 | 3 | 12,0 | 2 | 5 |  |  |
| VAL (m+2:m+2) |  | 132,1 | |  | 100,0 | 3 | 12,0 | 2 | 5 |  |  |
| VAL (m+3:m+2) |  | 133,1 | |  | 100,0 | 3 | 12,0 | 2 | 5 |  |  |
| VAL (m+3:m+3) |  | 133,1 | |  | 101,0 | 3 | 12,0 | 2 | 5 |  |  |
| VAL (m+4:m+3) |  | 134,1 | |  | 101,0 | 3 | 12,0 | 2 | 5 |  |  |
| VAL (m+4:m+4) |  | 134,1 | |  | 102,0 | 3 | 12,0 | 2 | 5 |  |  |
| VAL (m+5:m+4) |  | 135,1 | |  | 102,0 | 3 | 12,0 | 2 | 5 |  |  |
| VAL (m+5:m+5) |  | 135,1 | |  | 103,0 | 3 | 12,0 | 2 | 5 |  |  |
| VAL (m+6:m+5) |  | 136,1 | |  | 103,0 | 3 | 12,0 | 2 | 5 |  |  |
