# Supplementary material for: Fluxome study of Pseudomonas fluorescens reveals major reorganisation of carbon flux through central metabolic pathways in response to inactivation of the anti-sigma factor MucA
Source: BMC Syst Biol. 2015 Feb 18;9:6. doi: 10.1186/s12918-015-0148-0 (PMC4351692; doi:10.1186/s12918-015-0148-0)
Supplement: Additional file 5: — Mass isotopomer distributions detected by LC-MS/MS ( Table S.5.1 ) and GS-MS/MS ( Table S.5.2 ) for three P. fluorescens cultivations with labeled fructose isotopomer mixtures. [file 12918_2015_148_MOESM5_ESM.docx]

**Supplementary Table S.4.1.** Mass isotopomer distributions detected by LC-MS/MS for three *P. fluorescens* cultivations with labeled fructose isotopomer mixtures. Labeling fractions are not corrected for natural abundance because contributions were much smaller than measurement STDs. Calculations based on two samples for the pre-CLE and three samples for each of the two main CLEs. For the pre-CLE the measurements of *FBP*, *GOLP*, and *RXP* were excluded in flux estimation. For the main CLEs the measurements of *3PG*, *FBP*, and *GOLP* were excluded in case of both strains. Abbreviations: CLE: carbon labeling experiment; MS: mass spectrometry; STD: standard deviation and as in Supplementary Table S.1.5.

| Measurements | Experimental design CLE | | |  | Main CLEs | | | | | | | |
| --- | --- | --- | --- | --- | --- | --- | --- | --- | --- | --- | --- | --- |
|  | wild-type | | |  | wild-type | | |  | *mucA-* Δ*algC* | | | |
|  | Average | STD | STD [%] |  | Average | STD | STD [%] |  | Average | STD | STD [%] | |
| 6PG (m+0:m+0) | 30,07 | 1,52 | 5 |  | 7,81 | 1,06 | 14 |  | 9,40 | 0,92 | 10 | |
| 6PG (m+1:m+0) | 31,25 | 2,41 | 8 |  | 30,53 | 1,13 | 4 |  | 18,43 | 0,62 | 3 | |
| 6PG (m+2:m+0) | 10,38 | 1,32 | 13 |  | 9,26 | 1,13 | 12 |  | 15,11 | 1,06 | 7 | |
| 6PG (m+3:m+0) | 11,92 | 2,45 | 21 |  | 10,88 | 0,93 | 9 |  | 16,83 | 1,05 | 6 | |
| 6PG (m+4:m+0) | 7,08 | 0,04 | 1 |  | 13,51 | 1,24 | 9 |  | 15,78 | 0,95 | 6 | |
| 6PG (m+5:m+0) | 3,11 | 0,94 | 30 |  | 9,72 | 1,07 | 11 |  | 13,29 | 1,01 | 8 | |
| 6PG (m+6:m+0) | 6,20 | 1,05 | 17 |  | 18,27 | 2,03 | 11 |  | 11,15 | 1,11 | 10 | |
| F1P (m+0:m+0) | - | - | - |  | - | - | - |  | - | - | | - |
| F1P (m+1:m+0) | 58,77 | 5,32 | 9 |  | 44,58 | 2,17 | 5 |  | 44,57 | 2,24 | 5 | |
| F1P (m+2:m+0) | - | - | - |  | - | - | - |  | - | - | - | |
| F1P (m+3:m+0) | - | - | - |  | - | - | - |  | - | - | - | |
| F1P (m+4:m+0) | - | - | - |  | - | - | - |  | - | - | - | |
| F1P (m+5:m+0) | - | - | - |  | - | - | - |  | - | - | - | |
| F1P (m+6:m+0) | 41,23 | 5,32 | 13 |  | 55,42 | 2,17 | 4 |  | 55,43 | 2,24 | 4 | |
| G6P+ M6P+ F6P+ G1P (m+0:m+0) | 26,14 | 1,42 | 5 |  | 5,55 | 0,24 | 4 |  | 8,07 | 0,85 | 11 | |
| G6P+ M6P+ F6P+ G1P (m+1:m+0) | 36,32 | 2,07 | 6 |  | 31,37 | 0,42 | 1 |  | 18,97 | 0,39 | 2 | |
| G6P+ M6P+ F6P+ G1P (m+2:m+0) | 9,95 | 0,34 | 3 |  | 8,64 | 0,70 | 8 |  | 16,37 | 0,39 | 2 | |
| G6P+ M6P+ F6P+ G1P (m+3:m+0) | 7,77 | 1,12 | 14 |  | 8,80 | 0,30 | 3 |  | 17,69 | 0,53 | 3 | |
| G6P+ M6P+ F6P+ G1P (m+4:m+0) | 4,23 | 1,05 | 25 |  | 6,09 | 0,21 | 3 |  | 13,92 | 0,28 | 2 | |
| G6P+ M6P+ F6P+ G1P (m+5:m+0) | 2,16 | 0,43 | 20 |  | 5,55 | 0,10 | 2 |  | 9,73 | 0,36 | 4 | |
| G6P+ M6P+ F6P+ G1P (m+6:m+0) | 13,43 | 0,05 | 0 |  | 34,00 | 0,93 | 3 |  | 15,24 | 0,74 | 5 | |
| R5P (m+0:m+0) | 54,08 | 6,53 | 12 |  | 25,14 | 3,32 | 13 |  | 15,54 | 1,95 | 13 | |
| R5P (m+1:m+0) | 35,49 | 7,05 | 20 |  | 22,01 | 1,58 | 7 |  | 23,20 | 1,02 | 4 | |
| R5P (m+2:m+0) | - | - | - |  | 13,39 | 2,32 | 17 |  | 19,74 | 0,54 | 3 | |
| R5P (m+3:m+0) | 10,42 | 0,52 | 5 |  | 11,21 | 2,77 | 25 |  | 13,81 | 3,36 | 24 | |
| R5P (m+4:m+0) | - | - | - |  | 12,46 | 0,93 | 7 |  | 15,26 | 1,39 | 9 | |
| R5P (m+5:m+0) | - | - | - |  | 15,78 | 0,73 | 5 |  | 12,45 | 0,44 | 4 | |
| Ru5P+ X5P (m+0:m+0) | 46,71 | 0,43 | 1 |  | 21,15 | 3,35 | 16 |  | 15,17 | 1,02 | 7 | |
| Ru5P+ X5P (m+1:m+0) | 26,65 | 1,48 | 6 |  | 18,77 | 1,11 | 6 |  | 21,55 | 0,42 | 2 | |
| Ru5P+ X5P (m+2:m+0) | 14,39 | 1,04 | 7 |  | 20,45 | 1,99 | 10 |  | 18,82 | 0,64 | 3 | |
| Ru5P+ X5P (m+3:m+0) | 12,25 | 0,02 | 0 |  | 11,55 | 0,85 | 7 |  | 16,87 | 0,98 | 6 | |
| Ru5P+ X5P (m+4:m+0) | - | - | - |  | 12,45 | 1,48 | 12 |  | 15,60 | 0,62 | 4 | |
| Ru5P+ X5P (m+5:m+0) | - | - | - |  | 15,63 | 2,71 | 17 |  | 12,00 | 1,46 | 12 | |
| PEP (m+0:m+0) | 100,00 | - | - |  | 78,04 | 7,95 | 10 |  | 48,69 | 4,16 | 9 | |
| PEP (m+1:m+0) | - | - | - |  | - | - | - |  | 23,53 | 0,63 | 3 | |
| PEP (m+2:m+0) | - | - | - |  | - | - | - |  | 9,75 | 0,85 | 9 | |
| PEP (m+3:m+0) | - | - | - |  | 21,96 | 7,95 | 36 |  | 18,03 | 2,88 | 16 | |
| PRPP (m+0:m+0) | 82,40 | 6,26 | 8 |  | 22,49 | 2,03 | 9 |  | 22,61 | 3,85 | 17 | |
| PRPP (m+1:m+0) | 17,60 | 6,26 | 36 |  | 21,60 | 3,86 | 18 |  | 19,69 | 4,68 | 24 | |
| PRPP (m+2:m+0) | - | - | - |  | 18,80 | 2,67 | 14 |  | 17,14 | 1,61 | 9 | |
| PRPP (m+3:m+0) | - | - | - |  | 12,30 | 1,66 | 13 |  | 15,62 | 1,28 | 8 | |
| PRPP (m+4:m+0) | - | - | - |  | 15,76 | 1,28 | 8 |  | 17,04 | 2,88 | 17 | |
| PRPP (m+5:m+0) | - | - | - |  | 9,05 | 0,59 | 7 |  | 7,91 | 0,64 | 8 | |
| DHAP (m+0:m+0) | 88,03 | 3,68 | 4 |  | 60,74 | 1,44 | 2 |  | 48,34 | 2,03 | 4 | |
| DHAP (m+1:m+0) | 6,02 | 1,26 | 21 |  | 8,55 | 1,10 | 13 |  | 17,62 | 2,42 | 14 | |
| DHAP (m+2:m+0) | - | - | - |  | - | - | - |  | - | - | - | |
| DHAP (m+3:m+0) | 5,95 | 2,42 | 41 |  | 30,71 | 2,46 | 8 |  | 34,05 | 0,47 | 1 | |

**Supplementary Table S.4.2.** Mass isotopomer distributions detected by GC-MS/MS for three *P. fluorescens* cultivations with labeled fructose isotopomer mixtures. Calculations based on two samples for the pre-CLE and three samples for each of the two main CLEs. Labeling fractions are not corrected for natural abundance introduced by derivatization reagents because contributions are much smaller than the measurement STDs. For the pre-CLE the measurements of *FUM*, *MET*, and *THR* were excluded in flux estimation. Likewise, for the main CLEs the measurements of *CIT*, *FUM*, *GLN*, *MET*, *OGA*, *SUC*, *THR*, and *TYR* were excluded for the wild-type and measurements of *CIT*, *FUM*, *GLN*, *SUC*, and *THR* for the *mucA-*Δ*algC* stain, respectively. Abbreviations: CLE: carbon labeling experiment; MS: mass spectrometry; STD: standard deviation and as in Supplementary Table S.1.5.

| Measurement | Experimental design CLE | | |  | Main CLEs | | | | | | |
| --- | --- | --- | --- | --- | --- | --- | --- | --- | --- | --- | --- |
|  | wild-type | | |  | wild-type | | |  | *mucA-* Δ*algC* | | |
|  | Average | STD | STD [%] |  | Average | STD | STD [%] |  | Average | STD | STD [%] |
| ALA (m+0:m+0) | 65,55 | 1,57 | 2 |  | 50,04 | 1,63 | 3 |  | 42,12 | 1,19 | 3 |
| ALA (m+1:m+0) | - | - | - |  | 0,62 | 0,06 | 10 |  | 0,66 | 0,02 | 3 |
| ALA (m+1:m+1) | 20,23 | 0,94 | 5 |  | 20,22 | 1,17 | 6 |  | 26,84 | 1,00 | 4 |
| ALA (m+2:m+0) | - | - | - |  | 1,09 | 0,10 | 9 |  | 1,04 | 0,05 | 5 |
| ALA (m+2:m+1) | 1,30 | 0,03 | 2 |  | 3,33 | 0,34 | 10 |  | 3,10 | 0,03 | 1 |
| ALA (m+2:m+2) | 12,92 | 1,83 | 14 |  | 24,45 | 0,43 | 2 |  | 25,87 | 0,73 | 3 |
| ALA (m+3:m+1) | - | - | - |  | - | - | - |  | - | - | - |
| ALA (m+3:m+2) | - | - | - |  | 0,25 | 0,02 | 8 |  | 0,31 | 0,01 | 4 |
| ALA (m+4:m+2) | - | - | - |  | - | - | - |  | 0,05 | 0,01 | 25 |
| ASP (m+0:m+0) | 52,69 | 1,50 | 3 |  | 43,35 | 4,70 | 11 |  | 24,54 | 1,17 | 5 |
| ASP (m+1:m+0) | 0,74 | 0,05 | 6 |  | - | - | - |  | 0,55 | 0,01 | 2 |
| ASP (m+1:m+1) | 29,92 | 1,41 | 5 |  | 27,25 | 2,04 | 7 |  | 33,46 | 0,34 | 1 |
| ASP (m+2:m+1) | 0,33 | 0,02 | 7 |  | - | - | - |  | 0,57 | 0,02 | 3 |
| ASP (m+2:m+2) | 13,31 | 0,42 | 3 |  | 21,14 | 2,41 | 11 |  | 27,49 | 0,60 | 2 |
| ASP (m+3:m+2) | - | - | - |  | - | - | - |  | 0,38 | 0,02 | 4 |
| ASP (m+3:m+3) | 3,02 | 0,24 | 8 |  | 8,26 | 0,32 | 4 |  | 12,41 | 0,29 | 2 |
| ASP (m+4:m+3) | - | - | - |  | - | - | - |  | 0,13 | 0,01 | 7 |
| ASP (m+4:m+4) | - | - | - |  | - | - | - |  | 0,41 | 0,04 | 10 |
| ASP (m+5:m+4) | - | - | - |  | - | - | - |  | - | - | - |
| ASP (m+5:m+5) | - | - | - |  | - | - | - |  | 0,06 | 0,00 | 3 |
| ASP (m+6:m+5) | - | - | - |  | - | - | - |  | - | - | - |
| GLU (m+0:m+0) | 31,38 | 0,66 | 2 |  | 11,64 | 0,14 | 1 |  | 10,72 | 1,24 | 12 |
| GLU (m+1:m+0) | 8,42 | 0,44 | 5 |  | 6,15 | 0,15 | 2 |  | 5,71 | 0,11 | 2 |
| GLU (m+1:m+1) | 23,95 | 0,53 | 2 |  | 15,76 | 0,20 | 1 |  | 15,34 | 0,19 | 1 |
| GLU (m+2:m+0) | 0,22 | 0,04 | 18 |  | 0,25 | 0,02 | 8 |  | 0,29 | 0,00 | 1 |
| GLU (m+2:m+1) | 8,59 | 0,62 | 7 |  | 10,92 | 0,03 | 0 |  | 11,00 | 0,26 | 2 |
| GLU (m+2:m+2) | 14,84 | 0,28 | 2 |  | 16,90 | 0,27 | 2 |  | 16,99 | 0,14 | 1 |
| GLU (m+3:m+1) | - | - | - |  | 0,38 | 0,02 | 7 |  | 0,41 | 0,00 | 1 |
| GLU (m+3:m+2) | 5,67 | 0,26 | 5 |  | 13,74 | 0,13 | 1 |  | 13,77 | 0,25 | 2 |
| GLU (m+3:m+3) | 4,07 | 0,34 | 8 |  | 8,82 | 0,03 | 0 |  | 9,30 | 0,37 | 4 |
| GLU (m+4:m+2) | - | - | - |  | 0,32 | 0,03 | 10 |  | 0,36 | 0,00 | 1 |
| GLU (m+4:m+3) | 1,73 | 0,16 | 9 |  | 8,06 | 0,27 | 3 |  | 8,54 | 0,29 | 3 |
| GLU (m+4:m+4) | 0,73 | 0,13 | 17 |  | 3,08 | 0,09 | 3 |  | 3,28 | 0,01 | 0 |
| GLU (m+5:m+3) | - | - | - |  | 0,14 | 0,04 | 25 |  | 0,18 | 0,00 | 2 |
| GLU (m+5:m+4) | 0,40 | 0,08 | 20 |  | 3,56 | 0,02 | 1 |  | 3,70 | 0,04 | 1 |
| GLU (m+5:m+5) | - | - | - |  | 0,16 | 0,02 | 15 |  | 0,16 | 0,00 | 2 |
| GLU (m+6:m+4) | - | - | - |  | - | - | - |  | 0,06 | 0,00 | 5 |
| GLU (m+6:m+5) | - | - | - |  | 0,13 | 0,01 | 9 |  | 0,16 | 0,01 | 4 |
| GLU (m+6:m+6) | - | - | - |  | - | - | - |  | 0,02 | 0,00 | 9 |
| GLU (m+7:m+5) | - | - | - |  | - | - | - |  | - | - | - |
| GLU (m+7:m+6) | - | - | - |  | - | - | - |  | 0,02 | 0,01 | 23 |
| GLU (m+7:m+7) | - | - | - |  | - | - | - |  | - | - | - |
| GLU (m+8:m+6) | - | - | - |  | - | - | - |  | - | - | - |
| GLU (m+8:m+7) | - | - | - |  | - | - | - |  | - | - | - |
| GLU (m+9:m+7) | - | - | - |  | - | - | - |  | - | - | - |
| GLX (m+0:m+0) | 57,59 | 2,99 | 5 |  | 38,07 | 4,71 | 12 |  | 48,35 | 19,72 | 41 |
| GLX (m+1:m+0) | 1,70 | 0,12 | 7 |  | 1,62 | 0,10 | 6 |  | 4,13 | 1,69 | 41 |
| GLX (m+1:m+1) | 27,91 | 1,84 | 7 |  | 31,39 | 2,32 | 7 |  | 23,47 | 10,33 | 44 |
| GLX (m+2:m+0) | 0,43 | 0,06 | 14 |  | 0,85 | 0,05 | 6 |  | 1,85 | 0,48 | 26 |
| GLX (m+2:m+1) | 12,27 | 1,04 | 8 |  | 26,95 | 2,42 | 9 |  | 21,29 | 10,86 | 51 |
| GLX (m+2:m+2) | - | - | - |  | 0,33 | 0,09 | 26 |  | 0,25 | 0,18 | 70 |
| GLX (m+3:m+0) | - | - | - |  | 0,01 | 0,01 | 71 |  | 0,02 | 0,01 | 59 |
| GLX (m+3:m+1) | 0,11 | 0,03 | 24 |  | 0,42 | 0,06 | 14 |  | 0,34 | 0,24 | 70 |
| GLX (m+3:m+2) | - | - | - |  | 0,27 | 0,02 | 8 |  | 0,19 | 0,19 | 98 |
| GLX (m+4:m+1) | - | - | - |  | 0,09 | 0,03 | 33 |  | 0,09 | 0,08 | 87 |
| GLX (m+4:m+2) | - | - | - |  | - | - | - |  | - | - | - |
| GLX (m+5:m+2) | - | - | - |  | - | - | - |  | - | - | - |
| GLY (m+0:m+0) | - | - | - |  | 76,18 | 3,39 | 4 |  | 64,00 | 0,98 | 2 |
| GLY (m+1:m+0) | - | - | - |  | - | - | - |  | 0,90 | 0,09 | 10 |
| GLY (m+1:m+1) | - | - | - |  | 23,82 | 3,39 | 14 |  | 35,10 | 0,98 | 3 |
| GLY (m+2:m+1) | - | - | - |  | - | - | - |  | - | - | - |
| GLY (m+2:m+2) | - | - | - |  | - | - | - |  | - | - | - |
| GLY (m+3:m+2) | - | - | - |  | - | - | - |  | - | - | - |
| ILE (m+0:m+0) | 35,90 | 0,48 | 1 |  | 14,41 | 1,73 | 12 |  | 11,03 | 1,12 | 10 |
| ILE (m+1:m+0) | 2,79 | 0,33 | 12 |  | 1,83 | 0,43 | 23 |  | 2,10 | 0,19 | 9 |
| ILE (m+1:m+1) | 21,32 | 1,33 | 6 |  | 12,40 | 0,72 | 6 |  | 12,90 | 0,60 | 5 |
| ILE (m+2:m+0) | - | - | - |  | - | - | - |  | - | - | - |
| ILE (m+2:m+1) | 8,57 | 0,59 | 7 |  | 9,26 | 1,02 | 11 |  | 8,45 | 0,68 | 8 |
| ILE (m+2:m+2) | 16,53 | 1,20 | 7 |  | 19,40 | 1,68 | 9 |  | 17,41 | 1,43 | 8 |
| ILE (m+3:m+1) | - | - | - |  | - | - | - |  | - | - | - |
| ILE (m+3:m+2) | 5,31 | 0,65 | 12 |  | 9,50 | 0,57 | 6 |  | 9,91 | 0,64 | 6 |
| ILE (m+3:m+3) | 4,87 | 0,49 | 10 |  | 10,73 | 1,30 | 12 |  | 11,82 | 1,25 | 11 |
| ILE (m+4:m+2) | - | - | - |  | - | - | - |  | 0,14 | 0,08 | 56 |
| ILE (m+4:m+3) | 3,01 | 0,41 | 13 |  | 11,39 | 1,77 | 16 |  | 11,27 | 0,51 | 5 |
| ILE (m+4:m+4) | 1,71 | 0,34 | 20 |  | 4,67 | 1,40 | 30 |  | 6,14 | 0,27 | 4 |
| ILE (m+5:m+3) | - | - | - |  | - | - | - |  | - | - | - |
| ILE (m+5:m+4) | - | - | - |  | 4,49 | 0,16 | 4 |  | 5,06 | 0,63 | 12 |
| ILE (m+5:m+5) | - | - | - |  | - | - | - |  | 1,45 | 0,07 | 5 |
| ILE (m+6:m+4) | - | - | - |  | - | - | - |  | - | - | - |
| ILE (m+6:m+5) | - | - | - |  | 1,94 | 0,75 | 39 |  | 2,31 | 0,23 | 10 |
| ILE (m+6:m+6) | - | - | - |  | - | - | - |  | - | - | - |
| ILE (m+7:m+5) | - | - | - |  | - | - | - |  | - | - | - |
| ILE (m+7:m+6) | - | - | - |  | - | - | - |  | - | - | - |
| ILE (m+7:m+7) | - | - | - |  | - | - | - |  | - | - | - |
| ILE (m+8:m+6) | - | - | - |  | - | - | - |  | - | - | - |
| ILE (m+8:m+7) | - | - | - |  | - | - | - |  | - | - | - |
| ILE (m+9:m+7) | - | - | - |  | - | - | - |  | - | - | - |
| LYS (m+00:m+00) | 33,62 | 1,77 | 5 |  | - | - | - |  | 5,61 | 4,87 | 87 |
| LYS (m+01:m+00) | 8,60 | 2,48 | 29 |  | - | - | - |  | 3,56 | 0,38 | 11 |
| LYS (m+01:m+01) | 24,90 | 0,59 | 2 |  | - | - | - |  | 15,90 | 3,04 | 19 |
| LYS (m+02:m+00) | - | - | - |  | - | - | - |  | - | - | - |
| LYS (m+02:m+01) | 8,02 | 1,61 | 20 |  | - | - | - |  | 10,33 | 2,02 | 20 |
| LYS (m+02:m+02) | 12,05 | 0,53 | 4 |  | - | - | - |  | 16,25 | 2,04 | 13 |
| LYS (m+03:m+00) | - | - | - |  | - | - | - |  | - | - | - |
| LYS (m+03:m+01) | - | - | - |  | - | - | - |  | - | - | - |
| LYS (m+03:m+02) | 9,65 | 1,29 | 13 |  | - | - | - |  | 14,96 | 2,37 | 16 |
| LYS (m+03:m+03) | 3,16 | 0,94 | 30 |  | - | - | - |  | 9,88 | 1,79 | 18 |
| LYS (m+04:m+01) | - | - | - |  | - | - | - |  | - | - | - |
| LYS (m+04:m+02) | - | - | - |  | - | - | - |  | - | - | - |
| LYS (m+04:m+03) | - | - | - |  | - | - | - |  | 14,73 | 1,14 | 8 |
| LYS (m+04:m+04) | - | - | - |  | - | - | - |  | 1,84 | 1,66 | 90 |
| LYS (m+05:m+02) | - | - | - |  | - | - | - |  | - | - | - |
| LYS (m+05:m+03) | - | - | - |  | - | - | - |  | - | - | - |
| LYS (m+05:m+04) | - | - | - |  | - | - | - |  | 5,61 | 2,57 | 46 |
| LYS (m+05:m+05) | - | - | - |  | - | - | - |  | - | - | - |
| LYS (m+06:m+03) | - | - | - |  | - | - | - |  | - | - | - |
| LYS (m+06:m+04) | - | - | - |  | - | - | - |  | - | - | - |
| LYS (m+06:m+05) | - | - | - |  | - | - | - |  | 1,33 | 1,56 | 117 |
| LYS (m+06:m+06) | - | - | - |  | - | - | - |  | - | - | - |
| LYS (m+07:m+04) | - | - | - |  | - | - | - |  | - | - | - |
| LYS (m+07:m+05) | - | - | - |  | - | - | - |  | - | - | - |
| LYS (m+07:m+06) | - | - | - |  | - | - | - |  | - | - | - |
| LYS (m+07:m+07) | - | - | - |  | - | - | - |  | - | - | - |
| LYS (m+08:m+05) | - | - | - |  | - | - | - |  | - | - | - |
| LYS (m+08:m+06) | - | - | - |  | - | - | - |  | - | - | - |
| LYS (m+08:m+07) | - | - | - |  | - | - | - |  | - | - | - |
| LYS (m+09:m+06) | - | - | - |  | - | - | - |  | - | - | - |
| LYS (m+09:m+07) | - | - | - |  | - | - | - |  | - | - | - |
| LYS (m+10:m+07) | - | - | - |  | - | - | - |  | - | - | - |
| MET (m+0:m+0) | 60,19 | 2,57 | 4 |  | 61,02 | 1,54 | 3 |  | 17,25 | 0,93 | 5 |
| MET (m+1:m+0) | 13,89 | 2,50 | 18 |  | - | - | - |  | 11,96 | 0,72 | 6 |
| MET (m+1:m+1) | 25,92 | 5,00 | 19 |  | 38,98 | 1,54 | 4 |  | 17,44 | 0,28 | 2 |
| MET (m+2:m+1) | - | - | - |  | - | - | - |  | 15,86 | 0,39 | 2 |
| MET (m+2:m+2) | - | - | - |  | - | - | - |  | 13,31 | 0,43 | 3 |
| MET (m+3:m+2) | - | - | - |  | - | - | - |  | 12,98 | 0,44 | 3 |
| MET (m+3:m+3) | - | - | - |  | - | - | - |  | 5,61 | 0,43 | 8 |
| MET (m+4:m+3) | - | - | - |  | - | - | - |  | 5,58 | 0,23 | 4 |
| MET (m+4:m+4) | - | - | - |  | - | - | - |  | - | - | - |
| MET (m+5:m+4) | - | - | - |  | - | - | - |  | - | - | - |
| MET (m+5:m+5) | - | - | - |  | - | - | - |  | - | - | - |
| MET (m+6:m+5) | - | - | - |  | - | - | - |  | - | - | - |
| OGA (m+0:m+0) | 61,80 | 8,46 | 14 |  | 34,41 | 17,43 | 51 |  | 17,04 | 5,72 | 34 |
| OGA (m+1:m+0) | 2,24 | 0,15 | 7 |  | 2,34 | 0,43 | 18 |  | 2,71 | 0,95 | 35 |
| OGA (m+1:m+1) | 12,31 | 0,82 | 7 |  | 8,21 | 1,49 | 18 |  | 8,62 | 3,89 | 45 |
| OGA (m+2:m+1) | 1,53 | 0,51 | 33 |  | 3,39 | 1,26 | 37 |  | 5,00 | 2,04 | 41 |
| OGA (m+2:m+2) | 17,36 | 6,64 | 38 |  | 15,12 | 3,17 | 21 |  | 15,91 | 0,37 | 2 |
| OGA (m+3:m+2) | - | - | - |  | 6,36 | 2,46 | 39 |  | 8,58 | 0,88 | 10 |
| OGA (m+3:m+3) | 4,75 | 1,41 | 30 |  | 7,74 | 2,33 | 30 |  | 10,84 | 1,55 | 14 |
| OGA (m+4:m+3) | - | - | - |  | 4,87 | 1,77 | 36 |  | 7,74 | 0,99 | 13 |
| OGA (m+4:m+4) | - | - | - |  | 8,73 | 3,06 | 35 |  | 9,81 | 4,07 | 41 |
| OGA (m+5:m+4) | - | - | - |  | 4,90 | 1,47 | 30 |  | 6,90 | 2,45 | 36 |
| OGA (m+5:m+5) | - | - | - |  | 2,43 | 0,87 | 36 |  | 3,88 | 2,57 | 66 |
| OGA (m+6:m+5) | - | - | - |  | 1,48 | 0,41 | 28 |  | 2,96 | 1,72 | 58 |
| PYR (m+0:m+0) | 100,00 | - | - |  | 37,49 | 3,19 | 8 |  | 36,97 | 1,52 | 4 |
| PYR (m+1:m+0) | - | - | - |  | 13,33 | 0,65 | 5 |  | 11,99 | 2,04 | 17 |
| PYR (m+1:m+1) | - | - | - |  | 10,31 | 1,49 | 14 |  | 14,12 | 1,90 | 13 |
| PYR (m+2:m+0) | - | - | - |  | - | - | - |  | - | - | - |
| PYR (m+2:m+1) | - | - | - |  | 7,71 | 1,07 | 14 |  | 13,39 | 3,11 | 23 |
| PYR (m+2:m+2) | - | - | - |  | - | - | - |  | 3,07 | 0,75 | 24 |
| PYR (m+3:m+1) | - | - | - |  | - | - | - |  | - | - | - |
| PYR (m+3:m+2) | - | - | - |  | 31,16 | 1,34 | 4 |  | 20,46 | 3,24 | 16 |
| PYR (m+3:m+3) | - | - | - |  | - | - | - |  | - | - | - |
| PYR (m+4:m+2) | - | - | - |  | - | - | - |  | - | - | - |
| PYR (m+4:m+3) | - | - | - |  | - | - | - |  | - | - | - |
| PYR (m+5:m+3) | - | - | - |  | - | - | - |  | - | - | - |
| PHE (m+00:m+00) | 36,15 | 1,67 | 5 |  | 17,68 | 0,80 | 5 |  | 8,72 | 0,96 | 11 |
| PHE (m+01:m+00) | - | - | - |  | 0,18 | 0,05 | 26 |  | 0,19 | 0,02 | 9 |
| PHE (m+01:m+01) | 18,64 | 0,69 | 4 |  | 9,61 | 0,29 | 3 |  | 10,26 | 0,15 | 1 |
| PHE (m+02:m+01) | - | - | - |  | 0,17 | 0,06 | 33 |  | 0,25 | 0,00 | 0 |
| PHE (m+02:m+02) | 18,82 | 0,50 | 3 |  | 16,31 | 0,27 | 2 |  | 15,77 | 0,24 | 2 |
| PHE (m+03:m+02) | - | - | - |  | 0,20 | 0,06 | 30 |  | 0,32 | 0,02 | 7 |
| PHE (m+03:m+03) | 12,00 | 0,27 | 2 |  | 13,41 | 0,27 | 2 |  | 17,18 | 0,19 | 1 |
| PHE (m+04:m+03) | - | - | - |  | 0,17 | 0,03 | 19 |  | 0,32 | 0,03 | 10 |
| PHE (m+04:m+04) | 8,10 | 0,44 | 5 |  | 14,46 | 0,17 | 1 |  | 16,04 | 0,31 | 2 |
| PHE (m+05:m+04) | - | - | - |  | 0,21 | 0,02 | 9 |  | 0,25 | 0,06 | 24 |
| PHE (m+05:m+05) | 4,19 | 0,40 | 10 |  | 11,33 | 1,40 | 12 |  | 13,42 | 0,21 | 2 |
| PHE (m+06:m+05) | - | - | - |  | - | - | - |  | 0,22 | 0,03 | 12 |
| PHE (m+06:m+06) | 2,10 | 0,11 | 5 |  | 9,86 | 0,22 | 2 |  | 9,75 | 0,27 | 3 |
| PHE (m+07:m+06) | - | - | - |  | - | - | - |  | 0,12 | 0,01 | 7 |
| PHE (m+07:m+07) | - | - | - |  | 3,60 | 0,06 | 2 |  | 4,68 | 0,10 | 2 |
| PHE (m+08:m+07) | - | - | - |  | - | - | - |  | 0,06 | 0,01 | 20 |
| PHE (m+08:m+08) | - | - | - |  | 2,79 | 0,24 | 9 |  | 2,12 | 0,23 | 11 |
| PHE (m+09:m+08) | - | - | - |  | - | - | - |  | - | - | - |
| PHE (m+09:m+09) | - | - | - |  | - | - | - |  | 0,32 | 0,10 | 30 |
| PHE (m+10:m+09) | - | - | - |  | - | - | - |  | - | - | - |
| PRO (m+0:m+0) | 38,67 | 1,79 | 5 |  | 18,02 | 0,53 | 3 |  | 16,80 | 0,71 | 4 |
| PRO (m+1:m+0) | 7,73 | 1,59 | 21 |  | 7,84 | 0,14 | 2 |  | 7,68 | 0,43 | 6 |
| PRO (m+1:m+1) | 24,46 | 1,34 | 5 |  | 14,95 | 0,62 | 4 |  | 14,57 | 0,28 | 2 |
| PRO (m+2:m+0) | - | - | - |  | - | - | - |  | 0,21 | 0,03 | 15 |
| PRO (m+2:m+1) | 7,20 | 0,36 | 5 |  | 11,07 | 0,48 | 4 |  | 10,85 | 0,50 | 5 |
| PRO (m+2:m+2) | 13,64 | 1,14 | 8 |  | 15,72 | 0,23 | 1 |  | 15,45 | 0,33 | 2 |
| PRO (m+3:m+1) | - | - | - |  | - | - | - |  | 0,27 | 0,03 | 10 |
| PRO (m+3:m+2) | 4,60 | 0,23 | 5 |  | 12,74 | 0,43 | 3 |  | 13,09 | 0,35 | 3 |
| PRO (m+3:m+3) | 2,52 | 0,71 | 28 |  | 7,52 | 0,28 | 4 |  | 7,71 | 0,17 | 2 |
| PRO (m+4:m+2) | - | - | - |  | - | - | - |  | 0,21 | 0,04 | 17 |
| PRO (m+4:m+3) | 1,18 | 0,37 | 31 |  | 6,69 | 0,37 | 6 |  | 7,27 | 0,11 | 1 |
| PRO (m+4:m+4) | - | - | - |  | 2,45 | 0,10 | 4 |  | 2,70 | 0,10 | 4 |
| PRO (m+5:m+3) | - | - | - |  | - | - | - |  | 0,09 | 0,02 | 23 |
| PRO (m+5:m+4) | - | - | - |  | 3,00 | 0,27 | 9 |  | 3,12 | 0,06 | 2 |
| PRO (m+5:m+5) | - | - | - |  | - | - | - |  | - | - | - |
| PRO (m+6:m+4) | - | - | - |  | - | - | - |  | - | - | - |
| PRO (m+6:m+5) | - | - | - |  | - | - | - |  | - | - | - |
| PRO (m+6:m+6) | - | - | - |  | - | - | - |  | - | - | - |
| PRO (m+7:m+5) | - | - | - |  | - | - | - |  | - | - | - |
| PRO (m+7:m+6) | - | - | - |  | - | - | - |  | - | - | - |
| PRO (m+8:m+6) | - | - | - |  | - | - | - |  | - | - | - |
| TYR (m+00:m+00) | 37,99 | 1,44 | 4 |  | 27,75 | 5,39 | 19 |  | 8,44 | 0,37 | 4 |
| TYR (m+01:m+00) | - | - | - |  | - | - | - |  | - | - | - |
| TYR (m+01:m+01) | 21,67 | 0,86 | 4 |  | - | - | - |  | 10,47 | 1,07 | 10 |
| TYR (m+02:m+01) | - | - | - |  | - | - | - |  | - | - | - |
| TYR (m+02:m+02) | 20,26 | 1,54 | 8 |  | 31,22 | 5,87 | 19 |  | 15,87 | 0,93 | 6 |
| TYR (m+03:m+02) | - | - | - |  | - | - | - |  | - | - | - |
| TYR (m+03:m+03) | 11,32 | 0,45 | 4 |  | 20,15 | 1,39 | 7 |  | 18,08 | 1,47 | 8 |
| TYR (m+04:m+03) | - | - | - |  | - | - | - |  | - | - | - |
| TYR (m+04:m+04) | 8,76 | 0,92 | 11 |  | 20,88 | 5,74 | 28 |  | 15,84 | 0,35 | 2 |
| TYR (m+05:m+04) | - | - | - |  | - | - | - |  | - | - | - |
| TYR (m+05:m+05) | - | - | - |  | - | - | - |  | 12,99 | 0,48 | 4 |
| TYR (m+06:m+05) | - | - | - |  | - | - | - |  | - | - | - |
| TYR (m+06:m+06) | - | - | - |  | - | - | - |  | 8,88 | 0,43 | 5 |
| TYR (m+07:m+06) | - | - | - |  | - | - | - |  | - | - | - |
| TYR (m+07:m+07) | - | - | - |  | - | - | - |  | 4,49 | 0,14 | 3 |
| TYR (m+08:m+07) | - | - | - |  | - | - | - |  | - | - | - |
| TYR (m+08:m+08) | - | - | - |  | - | - | - |  | 2,74 | 0,23 | 8 |
| TYR (m+09:m+08) | - | - | - |  | - | - | - |  | - | - | - |
| TYR (m+09:m+09) | - | - | - |  | - | - | - |  | 0,89 | 0,29 | 32 |
| TYR (m+10:m+09) | - | - | - |  | - | - | - |  | - | - | - |
| TYR (m+10:m+10) | - | - | - |  | - | - | - |  | 0,76 | 0,09 | 12 |
| TYR (m+11:m+10) | - | - | - |  | - | - | - |  | - | - | - |
| TYR (m+11:m+11) | - | - | - |  | - | - | - |  | 0,55 | 0,14 | 25 |
| TYR (m+12:m+11) | - | - | - |  | - | - | - |  | - | - | - |
| VAL (m+0:m+0) | 47,63 | 1,24 | 3 |  | 23,94 | 0,41 | 2 |  | 20,74 | 0,25 | 1 |
| VAL (m+1:m+0) | 0,36 | 0,02 | 6 |  | 0,37 | 0,04 | 11 |  | 0,41 | 0,01 | 3 |
| VAL (m+1:m+1) | 24,06 | 0,36 | 2 |  | 21,13 | 0,13 | 1 |  | 22,20 | 0,26 | 1 |
| VAL (m+2:m+1) | - | - | - |  | 0,49 | 0,05 | 11 |  | 0,52 | 0,01 | 3 |
| VAL (m+2:m+2) | 23,80 | 0,94 | 4 |  | 31,13 | 0,85 | 3 |  | 31,84 | 0,27 | 1 |
| VAL (m+3:m+2) | - | - | - |  | 0,41 | 0,04 | 9 |  | 0,45 | 0,01 | 2 |
| VAL (m+3:m+3) | 4,15 | 0,52 | 12 |  | 13,24 | 0,55 | 4 |  | 14,91 | 0,43 | 3 |
| VAL (m+4:m+3) | - | - | - |  | 0,19 | 0,01 | 4 |  | 0,22 | 0,01 | 6 |
| VAL (m+4:m+4) | - | - | - |  | 8,93 | 0,28 | 3 |  | 8,47 | 0,27 | 3 |
| VAL (m+5:m+4) | - | - | - |  | - | - | - |  | 0,07 | 0,01 | 9 |
| VAL (m+5:m+5) | - | - | - |  | 0,17 | 0,02 | 13 |  | 0,16 | 0,01 | 7 |
| VAL (m+6:m+5) | - | - | - |  | - | - | - |  | - | - | - |
