# Supplementary material for: Fluxome study of Pseudomonas fluorescens reveals major reorganisation of carbon flux through central metabolic pathways in response to inactivation of the anti-sigma factor MucA
Source: BMC Syst Biol. 2015 Feb 18;9:6. doi: 10.1186/s12918-015-0148-0 (PMC4351692; doi:10.1186/s12918-015-0148-0)
Supplement: Additional file 6: — Table S.6.1 presents values of simulated measurements for the main CLEs. [file 12918_2015_148_MOESM6_ESM.docx]

| **Supplementary Table S.5.1.** Values of simulated measurements for the main CLEs. Model format of measurements given in the syntax of 13CFLUX2 fml-files. ND: not detected; RM: removed measurement. | | |
| --- | --- | --- |
| **Simulated measurement** | **Strain** | |
| **(model format)** | **wild-type** | **mucA- ΔalgC** |
| Fructose uptake | 1,9626 | 1,2313 |
| Biomass production | 0,0404 | 0,0389 |
| ALA[1-2]#M0,1,2 | 0,4872 | 0,4539 |
|  | 0,2007 | 0,2544 |
|  | 0,3121 | 0,2917 |
| LYS#M0,1,2,3,4,5,6 | ND | 0,0829 |
|  | ND | 0,1727 |
|  | ND | 0,2448 |
|  | ND | 0,2302 |
|  | ND | 0,1659 |
|  | ND | 0,0762 |
|  | ND | 0,0274 |
| DHAP#M0,1,2,3 | 0,5347 | 0,4678 |
|  | 0,0652 | 0,1297 |
|  | 0,0115 | 0,0354 |
|  | 0,3886 | 0,3671 |
| F1P#M0,1,2,3,4,5,6 | 0,0080 | 0,0168 |
|  | 0,5605 | 0,5444 |
|  | 0,0299 | 0,0299 |
|  | 0,0039 | 0,0172 |
|  | 0,0004 | 0,0026 |
|  | 0,0116 | 0,0119 |
|  | 0,3856 | 0,3773 |
| PEP#M0,1,2,3 | 0,5757 | 0,4019 |
|  | 0,0251 | 0,1920 |
|  | 0,0110 | 0,1277 |
|  | 0,3882 | 0,2783 |
| PRPP#M0,1,2,3,4,5 | 0,2314 | 0,1947 |
|  | 0,2064 | 0,2202 |
|  | 0,1595 | 0,1763 |
|  | 0,1178 | 0,1587 |
|  | 0,1431 | 0,1448 |
|  | 0,1418 | 0,1053 |
| R5P#M0,1,2,3,4,5 | 0,2314 | 0,1947 |
|  | 0,2064 | 0,2202 |
|  | 0,1595 | 0,1763 |
|  | 0,1178 | 0,1587 |
|  | 0,1431 | 0,1448 |
|  | 0,1418 | 0,1053 |
| F6P#M0,1,2,3,4,5,6 | 0,0845 | 0,0645 |
|  | 0,3649 | 0,2402 |
|  | 0,1018 | 0,1619 |
|  | 0,0867 | 0,1809 |
|  | 0,0617 | 0,1709 |
|  | 0,0616 | 0,0952 |
|  | 0,2387 | 0,0864 |
| RX5P#M0,1,2,3,4,5 | 0,2248 | 0,1680 |
|  | 0,1867 | 0,2316 |
|  | 0,1851 | 0,1884 |
|  | 0,1302 | 0,1465 |
|  | 0,1244 | 0,1559 |
|  | 0,1488 | 0,1097 |
| OGA#M0,1,2,3,4,5 | RM | 0,1157 |
|  | RM | 0,2197 |
|  | RM | 0,2816 |
|  | RM | 0,2299 |
|  | RM | 0,1127 |
|  | RM | 0,0404 |
| GLX#M0,1,2 | 0,4034 | 0,3945 |
|  | 0,3435 | 0,3435 |
|  | 0,2531 | 0,2620 |
| 6PG#M0,1,2,3,4,5,6 | 0,0845 | 0,0645 |
|  | 0,3649 | 0,2402 |
|  | 0,1018 | 0,1619 |
|  | 0,0867 | 0,1809 |
|  | 0,0617 | 0,1709 |
|  | 0,0616 | 0,0952 |
|  | 0,2387 | 0,0864 |
| ASP[1-3:1-3]#M(0,0),(1,1),(2,2),(3,3) | 0,2593 | 0,2549 |
|  | 0,3476 | 0,3411 |
|  | 0,2647 | 0,2655 |
|  | 0,1284 | 0,1385 |
| PHE[1-9:1-9]#M(0,0),(1,1),(2,2),(3,3),(4,4),(5,5),(6,6),(7,7),(8,8),(9,9) | 0,1472 | 0,0587 |
|  | 0,0653 | 0,1040 |
|  | 0,1095 | 0,1258 |
|  | 0,1822 | 0,1767 |
|  | 0,1403 | 0,1690 |
|  | 0,1077 | 0,1421 |
|  | 0,1165 | 0,1122 |
|  | 0,0675 | 0,0621 |
|  | 0,0227 | 0,0351 |
|  | 0,0413 | 0,0142 |
| PRO[1-5:1-4]#M(0,0),(1,0),(1,1),(2,1),(2,2),(3,2),(3,3),(4,3),(4,4),(5,4) | 0,1263 | 0,1157 |
|  | 0,0629 | 0,0564 |
|  | 0,1584 | 0,1633 |
|  | 0,1129 | 0,1145 |
|  | 0,1668 | 0,1672 |
|  | 0,1387 | 0,1455 |
|  | 0,0855 | 0,0844 |
|  | 0,0815 | 0,0885 |
|  | 0,0269 | 0,0242 |
|  | 0,0401 | 0,0404 |
| TYR[1-9:1-9]#M(0,0),(1,1),(2,2),(3,3),(4,4),(5,5),(6,6),(7,7),(8,8),(9,9) | RM | 0,0587 |
|  | RM | 0,1040 |
|  | RM | 0,1258 |
|  | RM | 0,1767 |
|  | RM | 0,1690 |
|  | RM | 0,1421 |
|  | RM | 0,1122 |
|  | RM | 0,0621 |
|  | RM | 0,0351 |
|  | RM | 0,0142 |
| VAL[1-4:1-4]#M(0,0),(1,1),(2,2),(3,3),(4,4) | 0,2373 | 0,2060 |
|  | 0,1956 | 0,2309 |
|  | 0,3444 | 0,3295 |
|  | 0,1253 | 0,1484 |
|  | 0,0974 | 0,0851 |
| GLU[1-5:1-4]#M(0,0),(1,0),(1,1),(2,1),(2,2),(3,2),(3,3),(4,3),(4,4),(5,4) | 0,1263 | 0,1157 |
|  | 0,0629 | 0,0564 |
|  | 0,1584 | 0,1633 |
|  | 0,1129 | 0,1145 |
|  | 0,1668 | 0,1672 |
|  | 0,1387 | 0,1455 |
|  | 0,0855 | 0,0844 |
|  | 0,0815 | 0,0885 |
|  | 0,0269 | 0,0242 |
|  | 0,0401 | 0,0404 |
| GLY[1:1]#M(0,0),(1,1) | 0,5963 | 0,5944 |
|  | 0,4037 | 0,4056 |
| ILE[1-6:1-5]#M(0,0),(1,0),(1,1),(2,1),(2,2),(3,2),(3,3),(4,3),(4,4),(5,4),(5,5),(6,5) | 0,0936 | 0,0829 |
|  | 0,0327 | 0,0328 |
|  | 0,1412 | 0,1398 |
|  | 0,0801 | 0,0798 |
|  | 0,1632 | 0,1650 |
|  | 0,1165 | 0,1167 |
|  | 0,1084 | 0,1135 |
|  | 0,1157 | 0,1164 |
|  | 0,0463 | 0,0495 |
|  | 0,0621 | 0,0632 |
|  | 0,0113 | 0,0130 |
|  | 0,0288 | 0,0274 |
| MET[1-4:2-4]#M(0,0),(1,0),(1,1),(2,1),(2,2),(3,2),(3,3),(4,3) | RM | 0,1465 |
|  | RM | 0,1084 |
|  | RM | 0,1961 |
|  | RM | 0,1450 |
|  | RM | 0,1526 |
|  | RM | 0,1129 |
|  | RM | 0,0796 |
|  | RM | 0,0589 |
| PYR[1-3:1-2]#M(0,0),(1,0),(1,1),(2,1),(2,2),(3,2) | 0,2994 | 0,3346 |
|  | 0,1878 | 0,1193 |
|  | 0,0953 | 0,1305 |
|  | 0,1054 | 0,1239 |
|  | 0,0210 | 0,0357 |
|  | 0,2912 | 0,2560 |
